# Phenotype screens of murine pancreatic cancer identify a Tgf-**α**-Ccl2-paxillin axis driving human-like neural invasion

**DOI:** 10.1172/JCI166333

**Published:** 2023-08-22

**Authors:** Xiaobo Wang, Rouzanna Istvanffy, Linhan Ye, Steffen Teller, Melanie Laschinger, Kalliope N. Diakopoulos, Kıvanç Görgülü, Qiaolin Li, Lei Ren, Carsten Jäger, Katja Steiger, Alexander Muckenhuber, Baiba Vilne, Kaan Çifcibaşı, Carmen Mota Reyes, Ümmügülsüm Yurteri, Maximilian Kießler, Ibrahim Halil Gürçınar, Maya Sugden, Saliha Elif Yıldızhan, Osman Uğur Sezerman, Sümeyye Çilingir, Güldal Süyen, Maximilian Reichert, Roland M. Schmid, Stefanie Bärthel, Rupert Oellinger, Achim Krüger, Roland Rad, Dieter Saur, Hana Algül, Helmut Friess, Marina Lesina, Güralp Onur Ceyhan, Ihsan Ekin Demir

**Affiliations:** 1 Department of Surgery, Klinikum rechts der Isar, Technical University of Munich, School of Medicine, Munich, Germany.; 2 CRC 1321 Modelling and Targeting Pancreatic Cancer, Klinikum rechts der Isar, Technical University of Munich, Munich, Germany.; 3 Department of General Surgery, The Second Xiangya Hospital, Central South University, Changsha, China.; 4 Neural Influences in Cancer (NIC) International Research Consortium.; 5 Pain Clinic, Department of Anesthesiology, First Affiliated Hospital of USTC (Anhui Provincial Hospital), Division of Life Sciences and Medicine, University of Science and Technology of China (USTC), Hefei, China.; 6 Comprehensive Cancer Center München, Chair for Tumor Metabolism, Klinikum rechts der Isar, Technical University of Munich, Munich, Bavaria, Germany.; 7 Charité - Universitätsmedizin Berlin, Berlin, Germany.; 8 Comparative Experimental Pathology and Institute of Pathology, Technical University of Munich, School of Medicine, Munich, Germany.; 9 Bioinformatics laboratory, Riga Stradins University, Riga, Latvia.; 10 Department of Bioinformatics and Medical Informatics; 11 Department of Physiology, Acibadem Mehmet Ali Aydinlar University, School of Medicine, Istanbul, Turkey.; 12 German Cancer Consortium (DKTK), Partner Site Munich, Munich, Germany.; 13 Department of Internal Medicine II, Klinikum rechts der Isar, Technical University of Munich, School of Medicine, Munich, Germany.; 14 Institute of Translational Cancer Research (TranslaTUM) and Experimental Cancer Therapy; 15 Institute of Molecular Oncology and Functional Genomics; 16 Institute of Experimental Oncology and Therapy Research, School of Medicine, Technical University Munich, Munich, Germany.; 17 Department of General Surgery, HPB-Unit, School of Medicine, Acibadem Mehmet Ali Aydinlar University, Istanbul, Turkey.; 18 Else Kröner Clinician Scientist Professor for Translational Pancreatic Surgery, Technical University of Munich, Munich, Germany.

**Keywords:** Oncology, Cancer, Innervation

## Abstract

Solid cancers like pancreatic ductal adenocarcinoma (PDAC), a type of pancreatic cancer, frequently exploit nerves for rapid dissemination. This neural invasion (NI) is an independent prognostic factor in PDAC, but insufficiently modeled in genetically engineered mouse models (GEMM) of PDAC. Here, we systematically screened for human-like NI in Europe’s largest repository of GEMM of PDAC, comprising 295 different genotypes. This phenotype screen uncovered 2 GEMMs of PDAC with human-like NI, which are both characterized by pancreas-specific overexpression of transforming growth factor α (TGF-α) and conditional depletion of p53. Mechanistically, cancer-cell-derived TGF-α upregulated CCL2 secretion from sensory neurons, which induced hyperphosphorylation of the cytoskeletal protein paxillin via CCR4 on cancer cells. This activated the cancer migration machinery and filopodia formation toward neurons. Disrupting CCR4 or paxillin activity limited NI and dampened tumor size and tumor innervation. In human PDAC, phospho-paxillin and TGF-α–expression constituted strong prognostic factors. Therefore, we believe that the TGF-α-CCL2-CCR4-p-paxillin axis is a clinically actionable target for constraining NI and tumor progression in PDAC.

## Graphical abstract

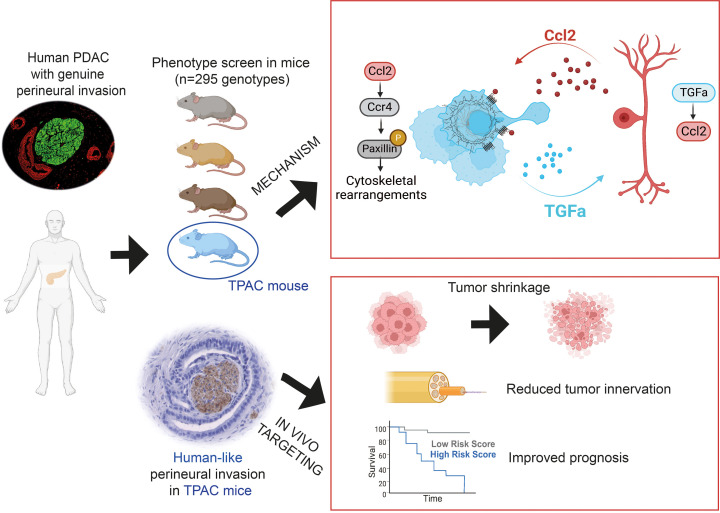

## Introduction

Pancreatic ductal adenocarcinoma (PDAC) is currently the third leading cause of cancer-associated death worldwide and is projected to become the second by 2030 (1). Remarkable progress has been made in the last 5 years in disentangling the complex genetic and molecular drivers and subtypes of PDAC. Oncogenic *Kras*-driven, genetically engineered mouse models (GEMMs) of PDAC have uncovered several aspects of the coevolution of cancer lesions and tumor microenvironment during carcinogenesis (2). As faithful models, they are powerful in modeling the molecular events that lead to metastasis, intratumoral heterogeneity, and several defining features of the tumor microenvironment, such as local immunosuppression (3).

**Figure 1:**
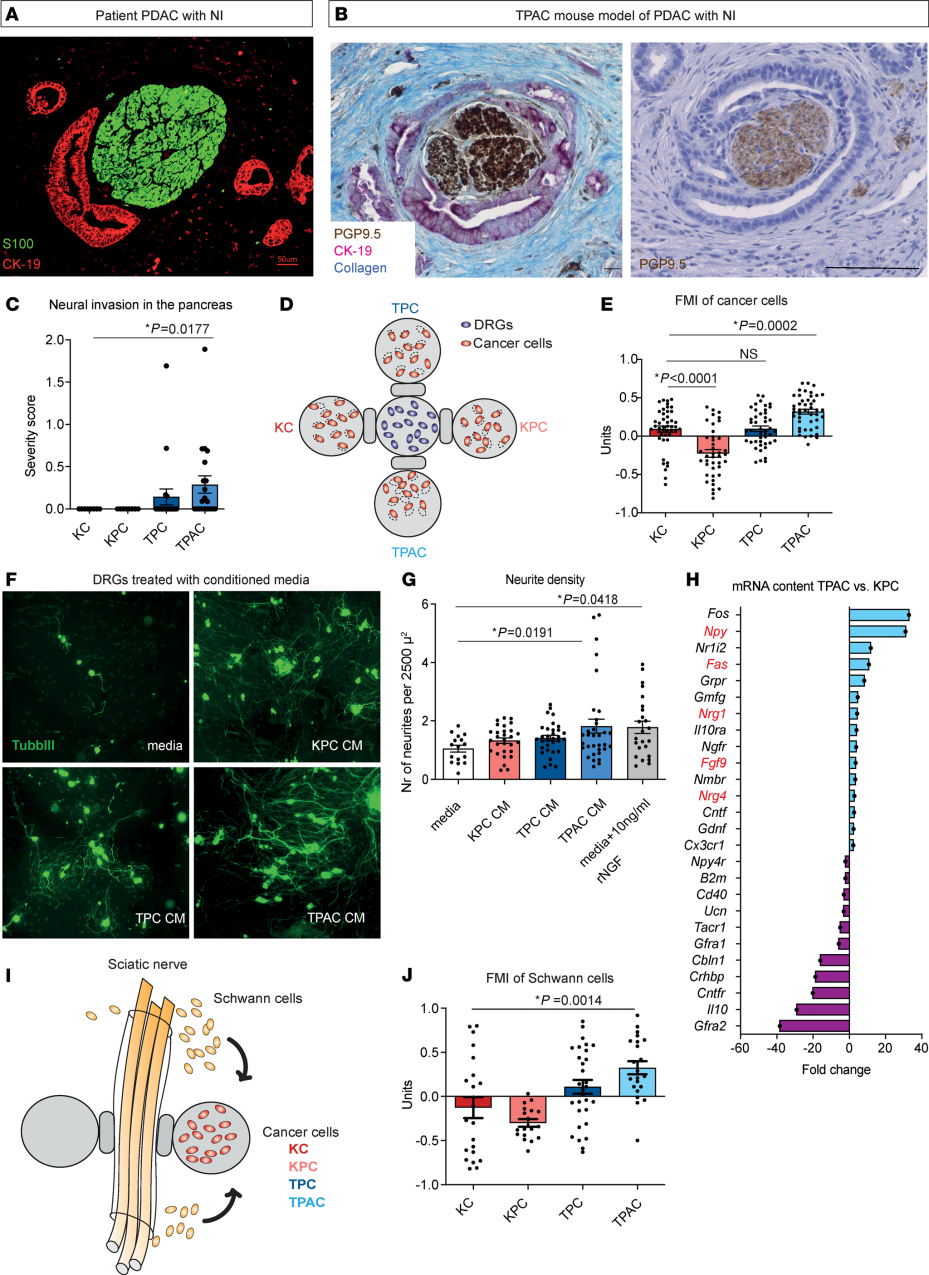
Mutant TPAC mice represent a model for human-like PNI. Representative images of genuine PNI in PDAC resection specimens (**A**) and in TPAC mouse mutants (**B**, please also see Supplemental Figure 1E for a broader view of the tumor area with PNI); pan-neural marker: PGP9.5 (brown), cancer cell marker: CK-19 (pink), and Aniline blue (for collagen). (**C**) Graphs showing the severity of NI in GEMMs of PDAC. (**D**) Scheme of migration assay of cancer cells and neurons from dorsal root ganglia (DRGs). (**E**) Graphs show the FMI of primary cancer cells isolated from KC, KPC, TPC, and TPAC mice in the 3D migration assay. (**F**) Representative images of DRG neurons treated with conditioned medium from KPC, TPC, and TPAC cancer cells, stained with neuronal marker β3 Tubulin (Tubβ3) antibody. (**G**) Results of the in vitro neuroplasticity assay of DRG neurons treated with supernatants of the primary cancer cells from the analyzed mouse genotypes. (**H**) Transcriptome analysis of TPAC- versus KPC-derived primary cancer cells. (**I**) Schematic representation of the 3D SC outgrowth assay. Sciatic nerves isolated from WT mice were placed between bridges connected to ECM gel drops containing primary murine cancer cells (KC, KPC, TPC, and TPAC) and empty ECM gel drops. The test was performed for 72 hours in culture media with CO_2_ supply. (**J**) Graphs showing the FMI of SCs. All results in the graphs are shown as mean ± SEM. For statistical analyses, Mann-Whitney U test (**C** and **E**), ordinary 1-way ANOVA (**G**), Kruskal-Wallis test, Dunnett’s test for multiple comparisons (**G** and **H**) and Shapiro-Wilk normality test (all panels) for distribution were used.

Human PDAC also exhibits a yet-unparalleled high frequency of neural invasion (NI) and neuroplastic alterations in the pancreas and in the central nervous system (4–6). NI was shown to be present in up to 100% of genuine ductal adenocarcinomas of the pancreas (7). NI in human PDAC typically manifests as perineural invasion (PNI), which implies the circular alignment of cancer cells along the epineural sheaths (7, 8) (Figure 1A). Importantly, in PDAC, the severity of NI, i.e., the penetration depth of cancer cells into the intrapancreatic nerves, is an independent prognostic factor for overall and disease-free survival, as well as local recurrence (9). Indeed, nerve-invading cancer cells use these as highways for rapid spread, which results in massive local tumor invasion, surgical irresectability, and severe pain (4, 10–12). In human PDAC, NI, starts, however, at the earliest stages of cancer, e.g. in T1 tumors (13). In fact, Schwann cells (SCs) of peripheral nerves were shown to emerge already around the precursor lesions of PDAC, i.e. pancreatic intraepithelial neoplasias (PanINs), which suggests that it is nerves — not cancer cells — that first migrate to initiate NI (14). Neural and extrapancreatic tumor invasion toward the spinal cord have been previously reported to be present in the oncogenic Kras-driven *p48-Cre;LSL-Kras^G12D^;p53^lox/–^* (KPC) GEMM of PDAC (15, 16). However, a direct comparison of NI in mouse PDAC to the morphology and molecular drivers of NI in human PDAC, has not yet been performed.

Considering this gap and the cardinal importance of NI for the course of human PDAC, we performed a systematic investigation of NI and neuromorphology in a large cohort of PDAC GEMMs comprising nearly 300 different allele combinations of oncogenes and tumor suppressors. Here, we uncover PDAC genotypes with human-like NI and point out the underlying molecular mechanisms that promote these human-like neurophenotypes. These models will serve as valuable tools for preclinical trials that aim to target NI in PDAC.

## Results

### Phenotype screens of GEMMs of PDAC reveal human-like neuro-invasive models.

The Collaborative Research Centre 1321 (CRC1321) in Munich has generated, cataloged, and characterized 295 different allele combinations of oncogenes and tumor suppressors in GEMMs of PDACs in the past 4 years. These invaluable tools, which enable a profound mechanistic understanding of the complex genetics and molecular drivers of PDAC, have served here as a unique platform for a phenotype screen for NI. The mouse alleles in this source comprise, among others, mutants of key genes and signaling pathways involved in pancreatic carcinogenesis, such as tumor suppressors (e.g., *p16^lox/lox^, Ink4a^lox/lox^,* and *p53^lox/lox^*), regulators of epithelial-mesenchymal transition (EMT) (*LSL-Snail^+/–^, Cdh1^lox/lox^,* and *Tnc^lox/lox^*), TGF-β signaling (*Tgfbr2^lox/lox^* and *Smad4^lox/lox^*), genetic reporters (*R26dTo^lox/lox^* and *dsRED-eGFP^lox/lox^*), viral targeting systems (*Tva* transgene), drivers of endodermal lineage specification (*Hnf4a^lox/lox^*), cell ablation alleles (DTA), and dual recombinase system alleles (*Pdx-Flp, FSF-Kras^G12D^, FSF-Rosa26-Cre^ER^*, *trp53*^frt/frt^, and *LSL-Kras^G12D^*). From each, we screened 2 H&E-stained slides, as well as 2 immunostained slides against the neuronal marker protein-gene-product 9.5 (PGP9.5) per mouse in each of a total of 295 different genotypes in the GEMMs from CRC1321. All genotypes were analyzed at an age at which they had developed overt invasive cancer (for a list of the analyzed age ranges and the number of mice per genotype, please refer to the Supplemental Tables 1 and 2; supplemental material available online with this article; https://doi.org/10.1172/JCI166333DS1)

In our screen, we detected a remarkably low frequency of human-like PNI in the primary tumor, which is defined as perineural alignment of cancer cells along the neural sheath (Figure 1A). In fact, the widely used oncogenic *Kras*-driven GEMMs of PDAC such as KC (*p48-Cre*; *LSL-Kras^G12D^*) or KPC (*p48-Cre*; *LSL-Kras^G12D^; trp53^lox/lox^*) did not exhibit PNI, although they harbored several enlarged nerves that were diffusely scattered in the tumor-infiltrated stroma. The lack of a “targeted” invasion of nerves by cancer cells in these important GEMMs of PDAC motivated us to further search for genotypes with exact human-like appearance of PNI. In our screen, we detected 2 genotypes with overt PDAC that fulfilled this criterion. Interestingly, both were driven by a pancreas-specific, transgenic overexpression of the transforming growth factor α (TGF-α) under the control of the elastase-1 promoter (*Ela1-TGF-α*): (a) *Ela1-TGF-α; p48-Cre; trp53^lox/lox^* (herein termed “TPC”) and (b) *Ela1-TGF-α; p48-Cre; trp53^lox/lox^*; *RelA^lox/lox^* (herein termed “TPAC”). We previously generated the latter phenotype to study (https://mediatum.ub.tum.de/?id=1271675) the influence of canonical NF-kB signaling on the growth dynamics of PDAC.

In the TPC genotype, we detected long-distance perineural contact between cancer cells and the enlarged pancreatic nerves, yet the cancer cells did not fully encircle the nerves, as is seen in human PDAC (Supplemental Figure 1E). In the TPAC genotype, however, the nerves were fully engulfed by cancer cells, which represents the typical appearance of PNI of human PDAC (Figure 1B and Supplemental Figure 1E). Accordingly, the histological severity of NI, assessed by a clinically established scoring system (9, 17, 18) (0, the tumor cells in no contact with nerves; 1, the tumor cells perineurally touch the nerves, and 2, the tumor cells invade inside the nerve, Supplemental Figure 1D), was markedly higher in TPAC and TPC mice compared with the *Kras*-driven KC and KPC mutants (TPAC: 0.26 ± 0.10, TPC: 0.14 ± 0.09, KPC: 0.0 ± 0.0, KC: 0.0 ± 0.0) (Figure 1C and Supplemental Figure 1D). Interestingly, the liver and lung metastases of TPAC mice did not exhibit NI (Supplemental Figure 2). To our knowledge, NI has also not yet been described in the metastases of human PDAC.

As the TPAC genotype exhibited a rather higher severity of NI and a more pronounced PNI than the TPC genotype, we also considered the possibility that the conditional/pancreas-specific loss of RelA/p65 (*p65^lox/lox^*) and the entailing suppression of the canonical NFkB signaling is linked to PNI. Therefore, we also analyzed the nerve morphology in another genotype with pancreas-specific loss of RelA/p65 (*p65^lox/lox^*) together with conditional activation of the oncogenic *Kras* and biallelic loss of p53 (*p48-Cre; LSL-Kras^G12D^; trp53^lox/lox^*; *RelA*^lox/lox,^ herein termed “KPAC”). We found that the KPAC mice did not harbor PNI and therefore displayed much lower NI severity scores (data not shown). Hence, this observation suggested that it is the TGF-α hypersignaling, rather than the loss of the canonical NFkB signaling, that promotes human-like NI in the TPAC and TPC models. We also dissected the pathways that are differentially regulated in the TPAC- versus TPC-derived cancer cells with the aim to uncover signaling events that may result in the more severe and human-like NI of TPAC versus TPC mice. In this transcriptome analysis, we found that the overwhelming majority, i.e., 50% of all differentially regulated pathways, were related to metabolic events, such as the fructose/mannose, histidine, sphingolipid, pentose phosphate pathway (Supplemental Figure 3). This suggests that metabolic alterations can further aggravate the human-like perineural invasive phenotype of murine pancreatic cancer.

Both genotypes were associated with extensive desmoplasia/fibrosis, more pronounced than in KPC mice. The TPC and TPAC tumors displayed a ductal-like phenotype and, accordingly, strongly expressed the ductal cancer cell marker cytokeratin 19 (CK-19) (Supplemental Figure 4A), yet the TPAC genotype with loss of *RelA/p65* and *p53* had an even more ductal-like appearance (Supplemental Figure 4, A and C), more desmoplasia (Supplemental Figure 4C), and a lower cancer cell proliferation rate (Supplemental Figure 4B) than the TPC mice. TGF-α is a known driver of acinar-to-ductal metaplasia (ADM). Importantly, the rate of ADM in the TPAC genotype was more pronounced both in vivo and in vitro than in TPC mice (not shown). Overall, these data suggested a more ductal, fibrotic, and slowly growing tumor phenotype in TPAC mice than in TPC or KPC mice. Accordingly, the overall survival of analyzed TPAC mice was significantly longer than that of TPC mice. TPAC mice had a median survival of 370 days compared to 297 days in TPC cohort (*P* < 0.0001, Supplemental Figure 1B). To further study the biological consequences of RelA loss in TPC mouse model, 46 TPC or TPAC tumors were analyzed. A remarkably high incidence of PDAC was observed in TPAC compared with TPC mice (75% versus 9.1%). In addition, RelA deficiency in TPC model resulted in a significant decrease in metastasis rate (31.8% in TPC versus 8.3% in TPAC, Supplemental Figure 4D).

These survival times clearly surpassed the lifespan of KPC mice (median 61 days), but were comparable to the lifespan of KC mice, which do not harbor a priori loss of p53 in carcinogenesis (Supplemental Figure 1A). However, KC mice, despite their slower tumorigenesis, did not exhibit human-like PNI. Interestingly, neural hypertrophy was present in all analyzed genotypes, independent of NI (Supplemental Figure 1F). To exclude the presence of mutant *Kras* in the TPAC mouse model, we performed targeted sequencing of the *Kras* locus in the spleen of the TPAC mice as well as in cancer cells isolated from the TPAC mice. As predicted, the tissues and cells from TPAC mice did not harbor *Kras* mutations and were thus *Kras* WT (Supplemental Table 3). Despite the absence of the oncogenic Kras mutation, we explored whether the TPAC cancer cells also exhibited elevated Ras activity. In Ras activity assays, we detected the Ras activity to be even higher in the isolated TPAC cancer cells than in KPC cancer cells, which confirms the strong Ras-activating capacity of TGF-α hypersignaling. (Supplemental Figure 4E). Altogether, these data strongly suggested that TGF-α signaling, without the need for mutant *Kras*, gives rise to slowly growing, highly fibrotic, ductal pancreatic cancer, which seem to be necessary for the emergence of human-like PNI in murine PDAC.

### TGF-α–associated PNI in murine PDAC genotypes correlates with neuroinvasiveness and SC chemoattraction ex vivo.

For functional analyses on neural invasion, we employed a series of ex vivo 3D culture setups. We first explored whether the enhanced neuroinvasiveness of the TPAC genotype is due to a superior chemoattraction of cancer cells of this genotype toward DRG neurons, which are the main source of the sensory innervation in the murine pancreas. For this, we placed primary cancer cells of each genotype in ECM droplets connected via bridges to droplets containing neurons isolated from dorsal root ganglia of P1-P3 C57Bl/6J mice and analyzed cell migration through time-lapse microscopy (Figure 1D). We did not identify any differences in the velocity of migrating cancer cells of all genotypes toward neurons (Supplemental Figure 1G). However, the forward migration index (FMI), which indicates directional chemotaxis, was much higher in TPAC cancer cells compared to the primary cancer cells of all other studied genotypes (TPAC: 0.32 ± 0.03, KC: 0.09 ± 0.04, KPC: –0.22 ± 0.05, TPC: 0.09 ± 0.03) (Figure 1E). We then analyzed whether the cancer cells of these genotypes also differ with regard to their neurotrophic attributes. Interestingly, conditioned medi from cultured primary cancer cells from TPAC mice augmented neurite outgrowth similarly to positive control media supplemented with 10 ng/mL nerve growth factor (NGF) (TPAC: 1.822; SFM: 1.05 neurites per 2,500 μm^2^) (Figure 1, F and G), whereas the number of neurites in cultures with conditioned media from the remaining genotypes did not differ from negative control (KPC: 1.235, TPC: 1.411 neurites per 2,500 μm^2^) (Figure 1, F and G). To explore molecular factors that drive a genotype-specific induction of neurite formation, we performed transcriptome arrays with the primary cancer cells from the different genotypes. Here, we found prominent upregulation of several neurotrophic and neurogenic factors in the TPAC cancer cells compared with KPC cancer cells. For example, *Fos* (33-fold upregulation), which is required for neurite elongation and crucial for neuronal differentiation (19, 20), and *neuropeptide Y/Npy* (31-fold upregulation), which plays an important role in axonogenesis (21), were the 2 most prominently upregulated neurotrophic genes (Figure 1H). In addition, TPAC cancer cells overexpressed *Fgf9*, which promotes proliferation of neuronal precursors (22), *Nrg1*, which has been shown to drive proliferation and/or induce myelin differentiation (23), and *Nrg4*, which is involved in the establishment of early sensory innervation in the skin (24) (Figure 1H).

Another recently discovered aspect of nerve-cancer interactions is the appearance of SCs around the premalignant lesions of pancreatic cancer, which, contrary to the traditional assumption, implies that the nerves and not the cancer cells migrate first during NI (14). Therefore, we analyzed the cancer tropism of SCs toward primary cancer cells isolated from KC, KPC, TPC, and TPAC genotypes in our SC outgrowth assay (14). For this purpose, we placed freshly isolated sciatic nerves from C57BL/6J mice and connected them via bridges to droplets containing cancer cells of the respective genotype on one side and empty ECM droplets on the other side. (Figure 1I). The velocity and distance of migrated SCs was unchanged toward the 4 analyzed mutated cancer cells (velocity: KC, 0.44 ± 0.05 μm/min; KPC, 0.47 ± 0.07 μm/min; TPC, 0.41 ± 0.04; TPAC 0.54 ± 0.07 μm/min; distance: KC, 104.6 ± 14.48 μm, KPC, 67.11 ± 9.16 μm; TPC, 96.36 ± 10.33 μm; TPAC, 106.6 ± 15.75 μm) (Supplemental Figure 1G). However, the FMI of SCs was increased toward TPAC cells (0.33 ± 0.07) when compared to KPC and KC cells with negative FMI (KPC: –0.3 ± 0.04; KC: –0.13 ± 0.12), indicating that there was not the same extent of directional migration of SC toward the KC and KPC cells compared to TPAC cells (Figure 1J).

To further explore the temporal relationship between TGF-α overexpression and NI, we also compared the expression of TGF-α in whole tissue lysates derived from KC mice with preinvasive PanIN lesions, compared with KPC, TPC and TPAC mice with over cancer. Here, we found that KC cells indeed had a lower expression level of TGF-α compared with KPC and TPAC cells (KC: 100.0% ± 53.7%, KPC: 256.5% ± 53.1%, TPC: 389.6% ± 49.5%, TPAC: 413.1% ± 56.5%, Supplemental Figure 4F). At the protein level, TPAC tissues had higher TGF-α levels than KPC tissues (KPC: 432.3 ± 22.1 ng/mL, TPC: 648.4 ± 75.2 ng/mL, TPAC: 830.5 ± 27.5 ng/mL), which suggests the gradual increase of TGF-α during perineurally invasive pancreatic carcinogenesis. We also analyzed the expression of TGF-α in human pancreatic lysates and found that in human chronic pancreatitis (CP), which is a potential precursor of human PDAC, the tissue levels of TGF-α were markedly lower than in human PDAC tissues (ΔΔ Ct/2^–ΔΔCt^ of CP: 0.14 ± 0.06 versus PDAC: 0.62 ± 0.18, p=0.017, Mann Whitney U test, Supplemental Figure 4F).

### Cancer-derived TGF-α upregulates CCL2 expression in pancreatic nerves.

To confirm the relevance of cancer-derived TGF-α in human PDAC, we analyzed 3 different publicly available single-cell RNA-Seq data sets of human PDAC and searched for the prime source of TGF-α in the human PDAC tissue. In line with our expectations, we detected cancer cells as the main source of TGF-α in human PDAC (Supplemental Figure 5), expressing 19.7-times higher amounts of TGF-α when compared with other cells in the tumor microenvironment, which had negligible amounts of TGF-α expression (Supplemental Figure 5).

**Figure 2:**
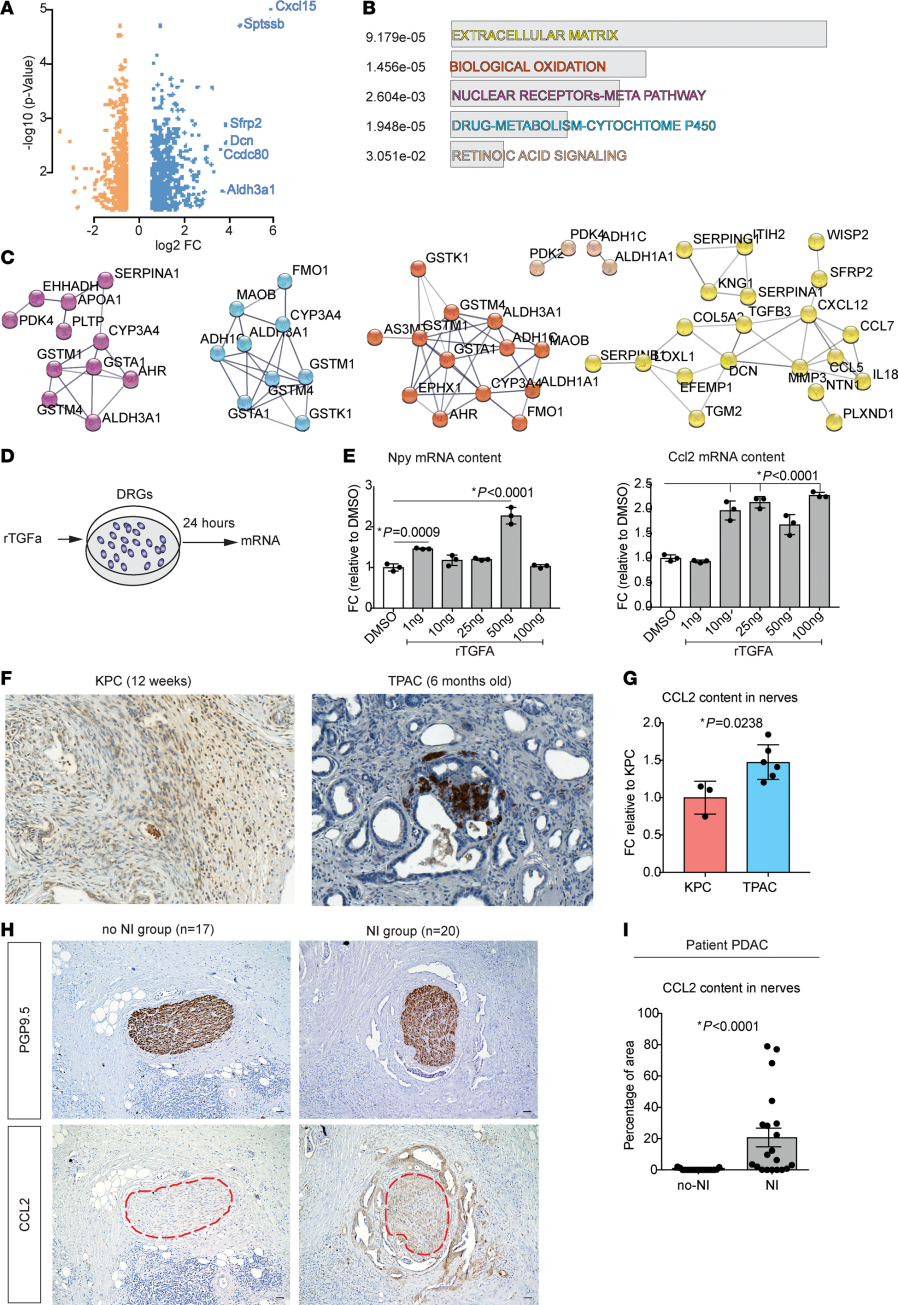
CCL2 induced by TGF-α is enriched in cocultures of cancer cells and DRG neurons, TPAC neurons, and in patient-derived PDAC samples with neural invasion. (**A**) Volcano plot of differentially expressed genes (DEGs) for the comparison of murine cancer cells derived from TPAC versus KPC mice. (**B**) Bar plot displaying a selected set of over-represented pathways among the DEGs for the comparison groups TPAC versus KPC. (**C**) Pathway-based interaction networks of DEGs for the comparison groups TPAC versus KPC, where network line thickness indicates the confidence of the interaction. (**D**) Experimental set-up: DRGs from neonatal mice were cultured for 2 days, and rTGF-α was added at concentrations of 1, 10, 25, 50, and 100 ng/mL for 24 hours. After the treatment, cells were used for RT-qPCR. (**E**) Graphs representing *Ccl2* and *Npy* mRNA content in DRGs after treatment with rTGF-α. (**F**) Representative images of pancreatic tumors from TPAC and KPC mice stained with PGP9.5 and CCL2 (both in brown). (**G**) Plots showing the colorimetric CCL2 content in nerves from TPAC and KPC tumors. (**H**) Representative images of consecutive sections of PDAC patient samples stained with CCL2 and S100 (both in brown) and counterstained with H&E. (**I**) Plots showing the colorimetric CCL2 content in the nerves of patient samples measured with the QuPath software. Scale bars: 50 μm. All results in the graphs are shown as mean ± SEM. For statistical analyses, ordinary 1-way ANOVA (**E**), Kruskal-Wallis test (**E**), Dunnett’s test for multiple comparisons (**E**), Mann-Whitney U test (**G** and **I**), and Shapiro-Wilk normality test for distribution (all panels) were used.

In the next step, we analyzed the transcriptome of pancreatic cancer cells from KPC and TPAC mutants using the Affymetrix Mouse Gene ST1.0 array with subsequent bioinformatics analysis at the GSEA platform. We identified 211 up-regulated and 55 down-regulated genes with a log_2_ fold change above 0.58 and below –0.58 and an FDR-adjusted *P* value (q value) under or equal to 0.05 within 28,941 genes by comparing TPAC with KPC cancer cells (Figure 2, A and B). Among the top 10 upregulated genes, we identified the *Cxcl15* chemokine involved in lung-specific neutrophil trafficking under normal and inflammatory conditions (25); serine palmitoyltransferase (*Sptssb*), which catalyses the first step of sphingolipid biosynthesis; secreted frizzled-related protein 2 (*Sfrp2*), a member of WNT signaling that promotes the neuronal differentiation potential of apical papilla stem cells (26); Decorin (*Dcn*), a small leucine-rich proteoglycan that plays a role in the assembly of collagen fibrils; coiled-coil domain-containing protein 80 (*Ccdc80*), which promotes melanoma cell migration via the FAK/E-cadherin pathway (27); and aldehyde dehydrogenase 3 family member A1 (Aldh3a1), which, together with ALDH1A1 is a biomarker for stem cell formation in pancreatic cancer (Figure 2A). Many of the upregulated genes in the TPAC cancer cells are involved in extracellular matrix formation (ECM) and related intracellular pathways. Interestingly, we also found strong upregulation of *Cxcl15*, *Sfrp2*, *Dcn,* and *Ccdc80* genes when we compared TPC with KPC cancer cells (Supplemental Figure 6A). In addition, the expression of genes that support neuritogenesis, namely *Npy* and *Fos,* was also increased in TPC- compared with KPC-derived cancer cells (Supplemental Figure 6B). We also found an enrichment of genes encoding the extracellular matrix — including ECM glycoproteins, collagens, and proteoglycans involved in biological oxidation and nuclear receptors and proteins encoding drug metabolism via cytochrome P450 and retinoic acid signaling — and depicted their numerous pathway interactions (Figure 2, B and C). Overall, these analyses showed that the TGF-α–driven PDAC GEMMs TPAC and TPC showed enhanced expression of neurotrophic and neurogenic factors compared with the KPC genotype-derived cancer cells.

Next, we performed a literature search for soluble factors that are known to be overexpressed under the influence of TGF-α. We found that in primary chondrocytes, TGF-α was shown to upregulate secretion of the chemokine CCL2 (28). Furthermore, CCL2 released by DRGs was shown to facilitate PNI of prostate cancer cells in vitro (29). In order to explore a possible link between TGF-α and CCL2 in our neuro-cancer interaction setups, we treated murine DRG cultures with recombinant TGF-α (rTGF-α). Here, we detected increased expression of the *Ccl2* gene within the DRG. In addition, *Npy* mRNA content was also upregulated (Figure 2, D and E). Accordingly, we found increased CCL2 protein in the supernatant of cocultured neurons and human cancer cell lines SU.86.86, T3M4, and DLD-1 (Supplemental Figure 3, C and D). Thus, CCL2 emerged here as a secreted molecule, which was induced by TGF-α, and potentially involved in the interactions between neurons and cancer cells.

To find out the potential role of CCL2 in NI in our GEMMs, we analyzed CCL2 content in the nerves within primary pancreatic tumors from KPC and TPAC mice (Figure 2, F and G). Here, we detected increased CCL2 protein immunoreactivity in the nerves of pancreatic tumors of TPAC mice compared with KPC. Similarly, we detected a more pronounced increase in CCL2 content in nerves in patient-derived PDAC samples with NI (NI group) compared with patient-derived samples without NI (No-NI group) (NI group: 20.78%; No-NI group: 0.3067%) (Figure 2, H and I).

To verify the DRG neurons as a major source of CCL2 in the PDAC context, we analyzed *Ccl2* expression levels in available single-cell RNA-Seq data sets of mouse DRG (30) (Supplemental Figure 7). Here, we detected DRG neurons, especially *CGRP-*α*^+^* and *nonpeptidergic nociceptor subtype* neurons, as the main source of CCL2 in DRG ganglia compared to lower expression of CCL2 in other cells in the ganglion (Supplemental Figure 7D). Next, we analyzed the CCL2 content in the PDAC tissue by semiquantitative scoring analysis of CCL2 immunoreactivity in 14 patients with PDAC (Supplemental Figure 7, A–C). We found that the CCL2 content was highest in nerves, which scored much higher when compared with acinus cells (*P* ˂ 0.0001), tumor cells (*P* ˂ 0.0001), immune cells (*P* ˂ 0.0001), and fibroblasts (*P* ˂ 0.001). In parallel, CCL2 immunostaining scoring in 10 TPAC mice showed that the nerves scored again highest when compared with acinus (*P* ˂ 0.0001), tumor (*P* ˂ 0.0001), immune cells (*P* ˂ 0.0001), and fibroblasts (*P* ˂ 0.0001) (Supplemental Figure 7, A–C).

In addition, we sorted out murine neurons out of DRG of TPAC mice and other cell types (cancer cells, myeloid cells, and lymphoid cells) from the primary tumors of TPAC mice and KPC and cross compared the expression levels of CCL2 (Supplemental Figure 7E). In line with above, we again detected the highest expression in the DRG neurons, which was much higher in TPAC-derived DRG neurons (610.9% ± 157.8% of KPC-derived neurons, *P* = 0.03), compared to KPC-derived DRG neurons (Supplemental Figure 7E).

We also quantified the mRNA content of the Ccl2 gene in granulocytes, monocytes, endothelial cells, mesenchymal cells, and cancer cells isolated from KPC and TPAC pancreatic tumors (n=3). Populations were identified as shown in the Supplemental Figure 8, A and B, including granulocytes as Cd45^+^Gr1^+^Cd11b^+^, monocytes as Cd45^+^Gr1medCd11b^+^, endothelial cells as Cd45^–^CD31^+^Cd326^–^, and cancer cells as Cd45-Cd31^+^CD326^+^ cells. With the exception of a lower proportion of monocytes in the pancreatic tumors of TPAC mice compared to KPC mice, other sorted cell populations were unchanged (Supplemental Figure 8C). Therefore, all these data underlined the DRG neurons and nerves as the leading and most dynamic source of CCL2 in PDAC.

The upregulation of CCL2 in DRG neurons upon exposition to TGF-α derived from cancer cells would necessitate the presence of EGFR receptor on DRG neurons. To prove the presence of this receptor on DRG neurons, we employed the spatial transcriptome technology (Nanostring GeoMx DSP) platform to quantify and to compare the expression of the whole mouse transcriptome in the DRG of our TPAC model in simultaneous comparison with oncogenic Kras-based mouse models of pancreatic cancer and with WT mice. This analysis allowed us to capture the expression of the whole transcriptome at single cell and spatial resolution in DRG neurons of different genotypes. Using this technology, we indeed detected EGFR receptor expression on the DRG neurons of the mice, and EGFR expression was significantly greater in the DRG neurons of TPAC mice (133.2% ± 14.0%), when compared to the oncogenic Kras-based KC mice (p48-Cre;LSL-KrasG12D; 100% ± 15.8%), which might explain the receptivity of TPAC-derived DRG fro TGF-α from cancer cells (Supplemental Figure 9, A and B). We complemented these analyses on murine DRG with the spatial transcriptome analysis of nerves within human pancreatic cancer specimens to explore whether we can detect the EGFR also in human nerves at the periphery that would bind TGF-α (Supplemental Figure 9, C and D). In the Nanostring GeoMx DSP spatial transcriptome-based analysis of EGFR expression, we found that the levels of EGFR expression in nerves with versus without NI did not vary, but EGFR was detectable in the nerves in all analyzed human PDAC specimens (Supplemental Figure 9, C and D). We confirmed the expression of EGFR in FACS-sorted DRG of TPAC, KPC, and WT mice (Supplemental Figure 9E), in nerves of samples derived from patients with PDAC (Supplemental Figure 9F) and in the DRG of TPAC mice (Supplemental Figure 9G) also via immunostaining. Therefore, these 3 lines of evidence further strengthened the notion of TGF-α responsiveness of DRG neurons in neuroinvasive PDAC.

### CCL2 activates the cancer cell cytoskeleton and enhances the migratory properties of pancreatic cancer cells via CCR4.

NI was originally believed to follow along paths of lowest physical resistance in the perineural area. However, numerous recent studies have convincingly shown that cancer cells, neurites, and SCs are chemoattracted very early during carcinogenesis to each other, which is orchestrated by soluble factors such as chemokines and neurotrophic factors (11, 31). Reorganization of the cytoskeleton is essential for the acquisition of migratory and invasive properties of cancer cells (32). One of the central molecules in the regulation of cytoskeletal reorganization is the multidomain scaffold protein paxillin (33). In this regard, phosphorylation of paxillin at tyrosine residues 31 (Tyr-31) and 118 (Tyr-118) by the nonreceptor protein tyrosine kinase SRC and focal adhesion kinase 1 (FAK1) is essential for cytoskeleton assembly and cancer cell migration (34–37).

**Figure 3:**
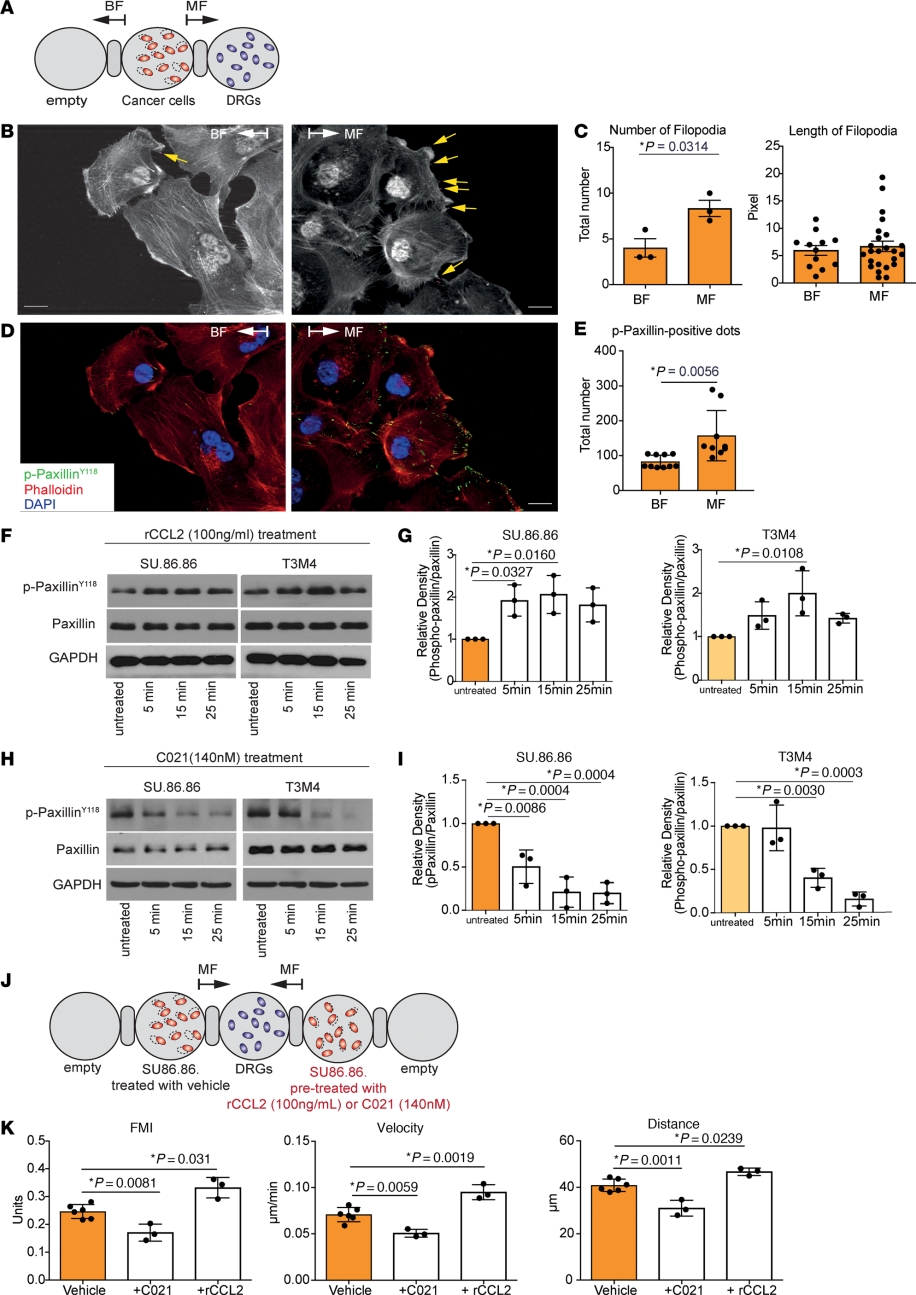
The migration behavior of cancer cells triggered by neurons is regulated by the CCL2/CCR4 axis. (**A**) Schematic representation of the 3D migration assay. Arrows indicate the direction of migrating cells. (**B**) Representative images of migrating SU.86.86 pancreatic cancer cells in the MF (toward DRGs) and BF (opposite to DRGs) analyzed by confocal microscopy and labeled filopodia (pink lines). (**C**) Diagrams showing the number and length of filopodia in the cancer cells in BF and MF, quantified with the FiloQuant software. (**D**) Representative images of SU.86.86 cancer cells from the migration assay, stained with phalloidin (red) and phospho-paxillinY118 (green), and counterstained with DAPI (blue). (**E**) Diagram showing the number of p-paxillin-positive points per ×10 magnification. (**F**) Representative Western blots of SU.86.86 and T3M4 cancer cells treated with recombinant CCL2 (100 ng/mL). (**G**) Graphs with relative content of proteins identified by Western blot measured with the ImageJ software (n=3 biological replicates). (**H**) Western blots of SU.86.86 and T3M4 cancer cells treated with CCR4 inhibitor C021 (140 nM). (**I**) Graphs with relative content of proteins identified by Western blot measured with the ImageJ software. (**J**) Scheme of experiment: cancer cells were pretreated with rCCL2 or C021 for 15 minutes and placed into the 3D migration assay with DRG neurons. As control, cells pretreated with vehicle were used. (**K**) Graphs indicating FMI, velocity, and migrated distance of cancer cells in the 3D migration assay. All results in graphs are shown as a mean ± SEM. For statistical analyses we used unpaired *t* test (**B** and **E**), 1-way ANOVA (**G**, **H**, and **K**), Dunnett’s multiple comparisons test (**G**, **I**, and **K**), and for the distribution, Shapiro-Wilk normality test (all panels). The *P* value ˂ 0.05 was considered to have significance. Scale bars: 20 μm.

To elucidate the cytoskeletal adaptation in cancer cells that are confronted with neurons, we investigated the morphology of filopodia and lamellipodia of cancer cells in the 3D migration assays with neurons. We quantified filopodia formation during migration toward DRGs using the FiloQuant software (Figure 3, A and B). Interestingly, cancer cells at the migration front (MF) showed a significant increase in filopodia number and length (Figure 3, B and C). In addition, we detected an increased number of p-paxillin-positive spots in the cytoskeleton of cancer cells in the MF toward DRGs compared with the back front (BF) that faces the empty ECM gel (Figure 3, D and E). These results suggested that cytoskeleton reorganization and microfilament formation in cancer cells are triggered by neurons.

Next, we asked whether CCL2, secreted by neural cells, affects the cytoskeletal mediators of migration in cancer cells. To this end, we treated human pancreatic cancer cells SU.86.86 and T3M4 for 15 and 25 minutes with conditioned media of murine DRG neurons (Supplemental Figure 6E). After 25 minutes of treatment, we observed increased phosphorylation of paxillin at Y118 in both cell lines, suggesting that DRG neurons secrete molecules that induce phosphorylation of paxillin. We next tested whether CCL2 directly induces phosphorylation of paxillin and treated SU.86.86 and T3M4 pancreatic cancer cells with 100 ng/mL of rCCL2 for 5, 15, and 25 minutes. Indeed, treatment of both patient-derived cancer cell lines with rCCL2 significantly enhanced the phosphorylation of paxillin already after 15 minutes of treatment (Figure 3, F and G), as well as of its downstream mediators Src and Erk (Supplemental Figure 6, G–J).

In cancer bone metastasis, CCL2 signaling is activated via binding of CCL2 ligand to the CCR2 and CCR4 receptors (38). In prostate cancer, CCL2-CCR2 is associated with PNI (29). However, in our experiments, the CCR2 antagonist RS-504393 had no effect on paxillin phosphorylation in either of tested pancreatic cancer cell lines (Supplemental Figure 6F). Remarkably, though, treatment of both cancer cell lines SU.86.86 and T3M4 with the CCR4 antagonist (C021) decreased paxillin phosphorylation in a time-dependent manner (Figure 3, H and I, and Supplemental Figure 6, H and I).

To test a functional effect of the CCL2-CCR4 pathway on migration, we pretreated cancer cells with rCCL2 or C021 for 15 minutes and used them in the migration assay in the presence of DRG neurons (Figure 3J). Vehicle-pretreated cancer cells served as a negative control. Here, treatment with rCCL2 increased cancer cell motility compared to vehicle-treated cells with a corresponding elevation in FMI (vehicle: 0.25 versus rCCL2 treatment: 0.33), higher velocity (vehicle: 0.075 μm/min versus rCCL2 treatment: 0.095 μm/min), and greater distance (vehicle: 41.61 μm versus rCCL2 treatment: 46.71 μm) (Figure 3K). Accordingly, CCR4 blockade with its inhibitor C021 resulted in decreased FMI (vehicle: 0.25 versus C021 treatment: 017), decreased velocity (vehicle: 0.067 μm/min versus C021 treatment: 0.051 μm/min), and shorter distance compared with vehicle control (vehicle: 40.12 μm versus C021 treatment: 31.03 μm) (Figure 3K). These results suggested that the CCL2-CCR4 cytokine signaling pathway plays an essential role in the affinity of pancreatic cancer cells toward neurons.

### P-paxillin is a prognostic factor in PDAC.

**Figure 4:**
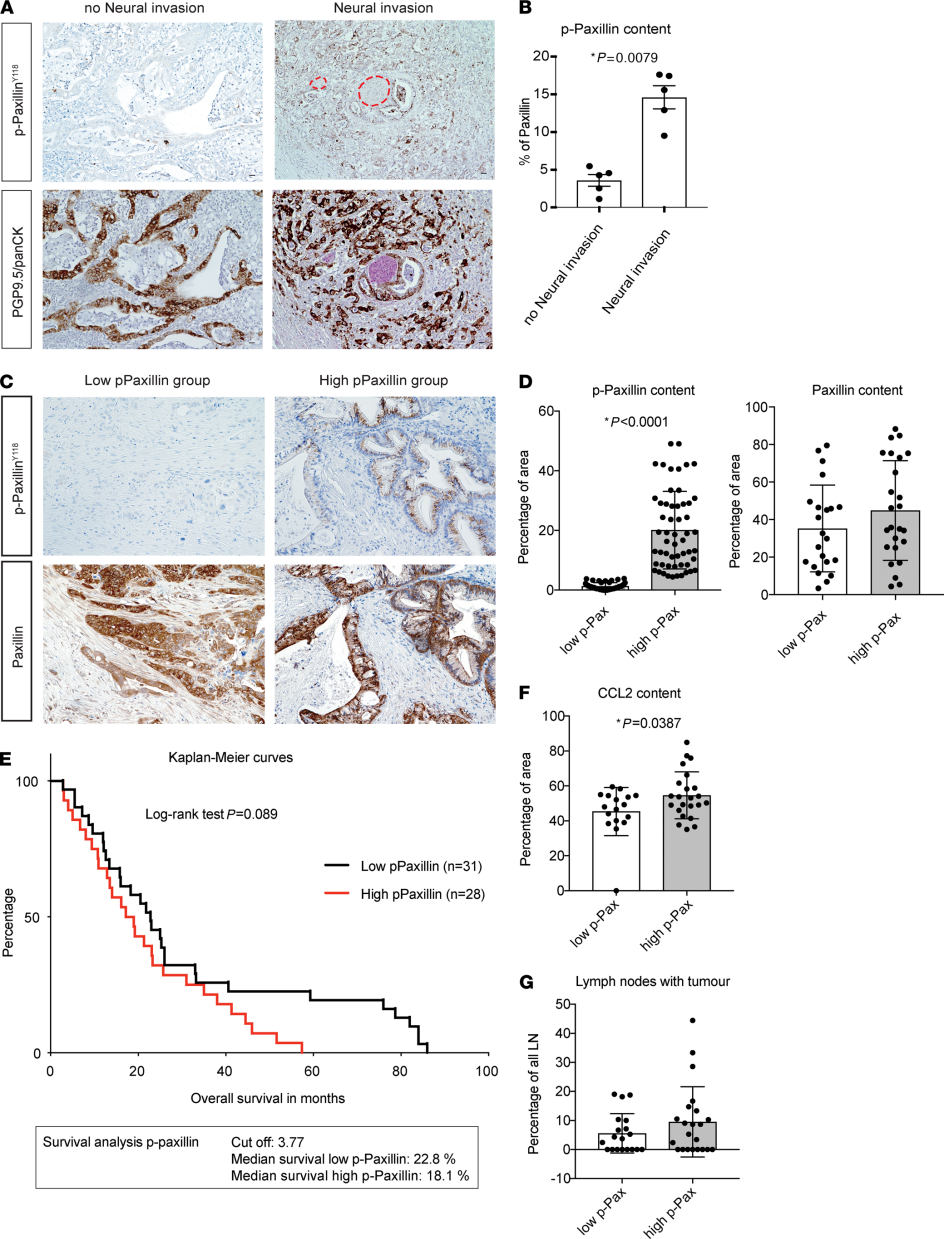
Paxillin phosphorylation in cancer cells is associated with poorer survival in patients with PDAC. (**A**) Representative images of consecutive sections from human PDAC resection specimens stained for p-paxillin (brown), the neural marker PGP9.5 (pink), cancer cell marker pan-CK (brown) and counterstained with haematoxylin. (**B**) Graphs showing the percentage of p-paxillin content in cancer cells located distal and proximal to nerves. (**C**) Representative images of consecutive sections from patient-derived PDAC samples stained with paxillin and p-paxillin (brown) and counterstained with hematoxylin. (**D**) Graphs indicating relative content of p-paxillin to paxillin in low-p-paxillin and high p-paxillin groups of patients with PDAC. (**E**) Kaplan-Meier curves showing percentage survival of patients with low p-paxillin content (black line) and high p-paxillin content (red line). The cut-off value for p-paxillin content was set at 4% stained cells in all analyzed areas. (**F**) Graphs showing the percentage of CCL2 content in low-p-paxillin and high p-paxillin groups of patients with PDAC. (**G**) Graphs showing the percentage of lymph nodes infiltrated with tumor cells to all lymph nodes analyzed in low-p-paxillin and high p-paxillin groups of patients with PDAC. Scale bars: 20 μm. All results in graphs are shown as a mean value ± SEM. For the statistical analyses we used Mann-Whitney test (**B**, **D**, and **F**), and the Mantel-Cox test (**E**). The *P* value ˂ 0.05 was considered to have significance.

We next interrogated whether paxillin phosphorylation is relevantly increased in human pancreatic cancer cells that are physically adjacent to nerves in the pancreas. To this end, we stained tumor paraffin sections from patients with PDAC with NI (NI group) and without NI (no NI group) with PGP9.5, pan-CK (for labeling cancer cells) and p-paxillin. We found that cancer cells located close to nerves (within 200 μm diameter) expressed more p-paxillin (15.57% of total tissue area) than cancer cells located in the pancreas distant from nerves (4.23% of total tissue area) (Figure 4, A and B). As NI is a strong and independent prognostic factor for overall survival in PDAC (39), we then analyzed whether p-paxillin content also relates to patient survival. First, we divided the patient samples into low p-paxillin (n=31) and high p-paxillin (n=23) groups based on a cut-off value of 3.77%, which corresponded to the median of the p-paxilin/paxillin ratio (Figure 4B). Here, the mean staining intensity value of the high p-paxillin group (16.72%) was significantly increased compared with the low p-paxillin group (0.62%) (Figure 4, C and D). In contrast, the total paxillin concentration in the tumors of both groups were comparable (Figure 4, C and D). As shown on the Kaplan-Meier curves, the survival time of patients from the low p-paxillin group (n=31; median survival rate: 22.8%) tended to be longer than that of patients from the high p-paxillin group (n=28; median survival rate: 18.1%) (Figure 4E). We also found that CCL2 immunoreactivity was increased in the high p-paxillin expression group (Figure 4F), suggesting that CCL2 overexpression is indeed linked to paxillin phosphorylation in human PDAC tissue. Analyzing the clinical parameters, we found that the high p-paxillin group had a higher percentage of patients with large tumors (T4>6 cm) than the low p-paxillin group (χ^2^: *P* = 0.01, Supplemental Figure 10, A and C). In addition, the percentage of moderately differentiated tumors (moderately differentiated (G2): 76%) was increased, whereas differentiated (G1) tumors were not detected in the high p-paxillin group compared with the low p-paxillin group (G2: 63%, G1: 9%) (χ^2^: *P* = 0.006, Supplemental Figure 10, A and C). We also observed a marked, but not significant, increase in the proportion of affected lymph nodes with tumour cells (Figure 4G). These data point out that the tumor size and higher tumor grading were indeed more prevalent in the high-paxillin group, which suggest that the survival difference of high versus low-paxillin groups might be indirect due to other clinicopathological parameters. Overall, these results also suggested that p-paxillin is a prognostic factor and may predict poor prognosis and aggressive tumor biology in human PDAC.

### Targeting of the CCL2-CCR4 axis in vivo limits NI and tumor innervation.

**Figure 5:**
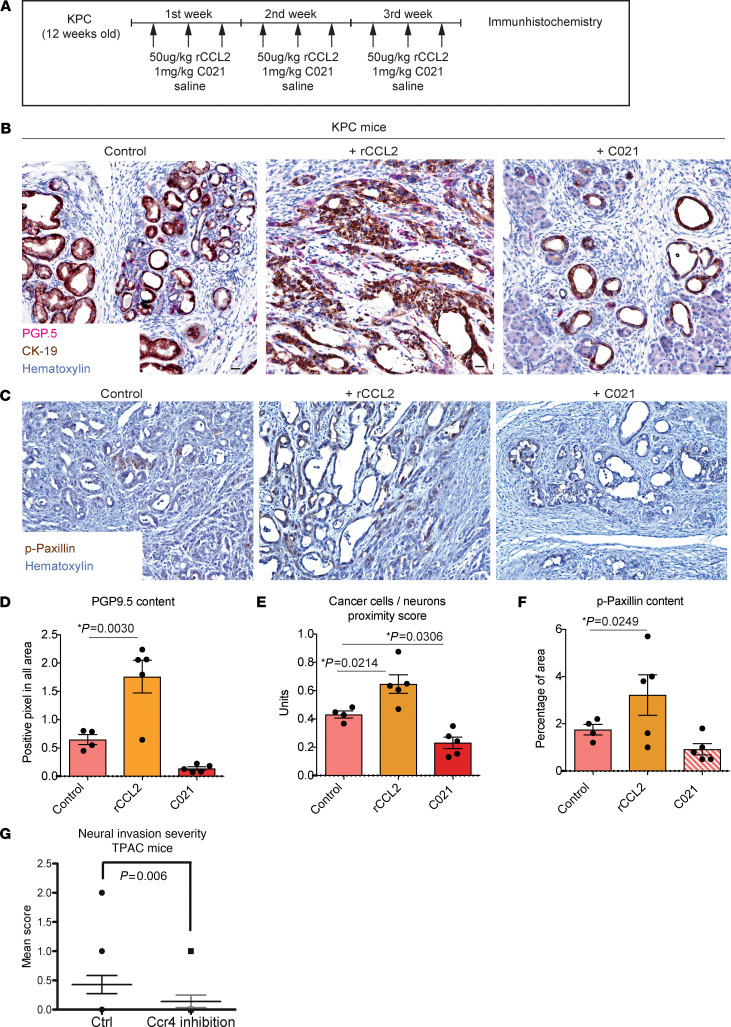
CCL2/CCR4 axis regulates paxillin phosphorylation and innervation in KPC mice. (**A**) Experimental design of rCCL2 and C021 inhibitor treatment: 12-week-old KPC mice (*n* = 5) were injected i.p. with rCCL2 or C021 every other day for 3 weeks with normal saline as control. (**B** and **C**) Representative images of consecutive sections from PDAC samples of KPC mice treated with rCCL2, C021, and control groups stained with the neural marker PGP9.5 (pink), cancer cell marker CK-19 (brown), p-paxillin (brown), and counterstained with haematoxylin. Plots show (**D**) PGP9.5 content, (**E**) score of cancer cell proximity to neurons, and (**F**) p-paxillin content in PDAC sections from treated KPC mice. (**G**) The NI score in the pancreatic tumors of TPAC mice treated with the CCR4 inhibitor versus control (solvent) substance. All results in graphs are shown as a mean value ± SEM. For the statistical analyses, we used ordinary 1-way ANOVA (**D**–**F**), Dunnett’s multiple comparisons test (**D**–**F**) and for the distribution, a *t* test (**G**) and Shapiro-Wilk normality test (all panels). The *P* value ˂ 0.05 was considered to have significance. Scale bars: 20 μm.

To understand the role of the CCL2-CCR4 axis in an organismal context, we modulated CCL2-CCR4 signaling in vivo by i.p. administration of recombinant CCL2 protein (50 μg/kg i.p.) or of the CCR4 receptor antagonist C021 (1 mg/kg), starting with KPC mice that exhibit hyperinnervation (Supplemental Figure 1F) without PNI (Figure 5A). No metastases were detected in the brain, lung, heart, liver, kidney, jejunum, and colon in the studied groups (Supplemental Figure 11). Here, the amounts of nerves as detected through intratumoral PGP9.5 immunostaining in the pancreas of KPC mice was increased after treatment with rCCL2 (mean 1.76) and dramatically decreased after treatment with C021 (mean 0.137) compared with KPC control animals (mean 0.647) (Figure 5, B and D). Although these mice lack human-like NI, we scrutinized whether this treatment resulted in a change in the physical proximity between nerves and cancer cells in the primary tumor. Indeed, the index for proximity between cancer and nerves was significantly increased after treatment with rCCL2 and decreased after CCR4 inhibition compared with control mice (mean index: rCLL2: 0.64, C021: 0.2, control: 0.43; mean scores: rCCL2: 28.2, C021: 8.8, control: 16.00) (Figure 5, B and E). Moreover, treatment with rCCL2 increased paxillin phosphorylation in tumor cells (mean: 3.13), while CCR4 inhibition decreased p-paxillin amounts (mean: 1.06) (Figure 5, C and F). Taken together, these results, in agreement with our in vitro observations, suggest that the CCL2-CCR4 pathway affects paxillin phosphorylation in cancer cells and the proximity of the cancer to neural cells, even in the absence of perineural lining of cancer cells.

To further confirm the in vivo relevance of CCR4 signaling on NI in the herein described TPAC model, we treated TPAC mice (aged 42 weeks) with the CCR4 inhibitor C021 3 times a week for 3 weeks and subsequently analyzed the severity of NI in the primary tumor via histology. Accordingly, the CCR4 inhibition remarkably reduced the NI severity in the TPAC mice (mean NI score of Control/ctrl solvent-treated mice: 0.4 ± 0.08 versus CCR4 inhibitor-treated: 0.1 ± 0.06), as assesses by the NI severity score (Figure 5G). CCR4 is known to exhibit relatively high expression levels also in T lymphocytes and macrophages (The Protein Atlas database: https://www.proteinatlas.org/ENSG00000183813-CCR4/single+cell+type). We therefore also explored the possibility that the amounts of these 2 immune cell subsets in the TPAC mice might be affected when the TPAC mice are treated with the CCR4 inhibitor. Here, we found that CCR4 inhibition did not alter the amounts of CD4^+^ T lymphocytes in the TPAC tumors (Control: 1.11 ± 0.28 versus CCR4-inhibited: 0.68 ± 0.20 cells/mm^2^) but, interestingly, also reduced the number of F4/80^+^ macrophages (Control: 7.26 ± 0.48 versus CCR4-inhibited: 2.56 ± 0.35 cells/mm^2^, Supplemental Figure 14, A and B), underlining an additional role for macrophages in the modulation of NI in murine PDAC.

### Inhibition of paxillin-Src-Erk signalosome reduces NI, innervation, and tumor size.

**Figure 6:**
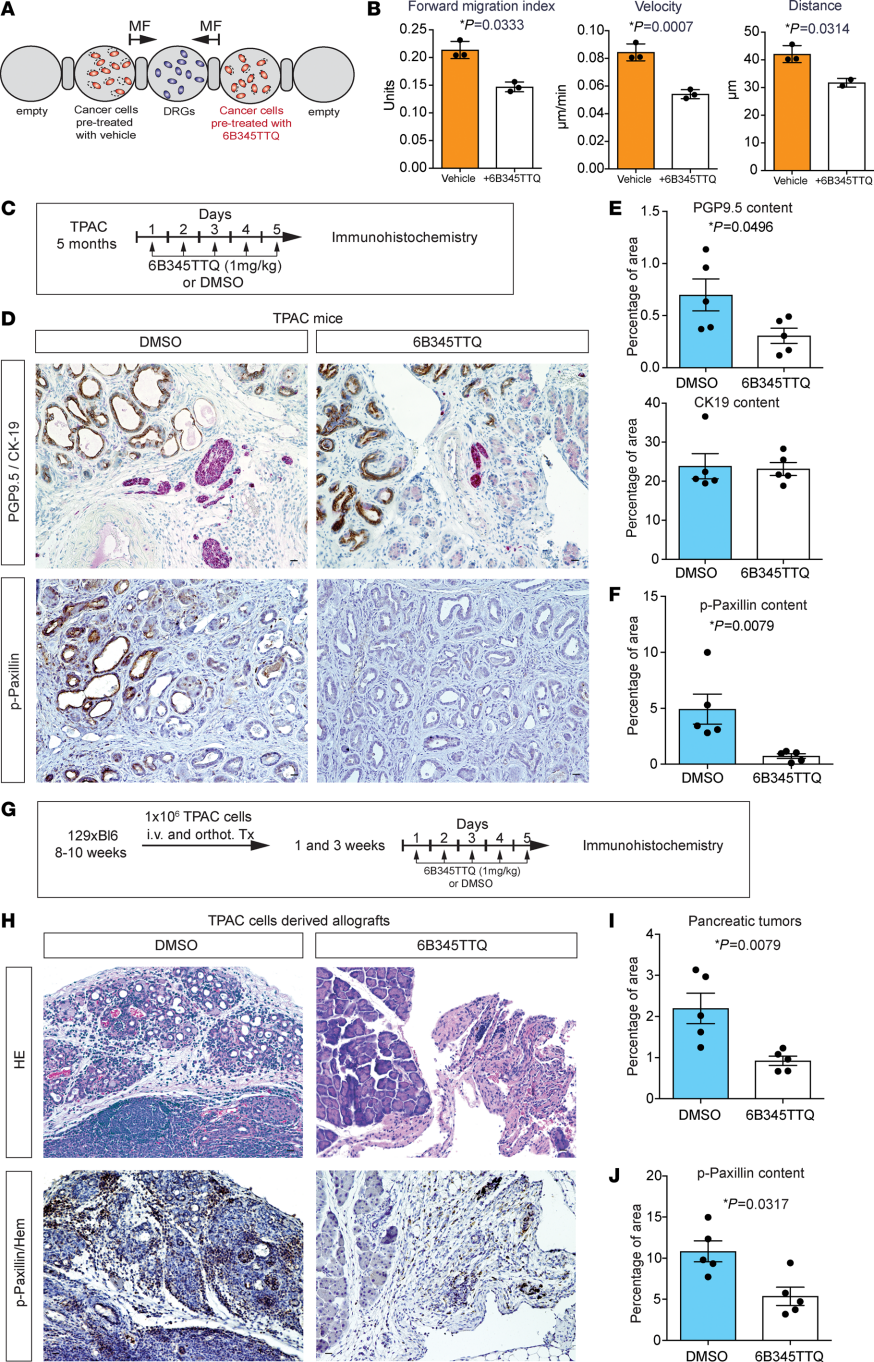
Inhibition of Paxillin-Src-Erk signalosome in vitro and in vivo. (**A**) Experimental design: SU86.86 cancer cells pretreated with the paxillin phosphorylation inhibitor 6-B345TTQ for 1 hour were used for a migration assay with murine DRG neurons. Cells pretreated with vehicle were used as control. (**B**) Graphs showing the FMI, speed, and distance of cells migrating to DRGs. (**C**) Scheme of in vivo treatment with 6-B345TTQ. 5-month-old TPAC mice were treated with 1 mg/kg of the inhibitor daily for 5 days for a total of 4 weeks. The control group was treated with DMSO. (**D**) Representative images of pancreatic tumors from TPAC mice treated with 6-B345TTQ and the control group, stained with the neural marker PGP9.5 (pink), cancer cell marker CK-19 (brown), p-paxillin (brown), and counterstained with haematoxilin (blue). The tumor injection bed is marked by the yellow border lines. Graphs show PGP9.5 and CK-19 content (**E**) and p-paxillin (**F**) as percentage of positively stained cells stained in the total area. (**G**) Scheme of treatment in mice allografted with TPAC cancer cells: 1 × 10^6^ cultured primary TPAC cancer cells were orthotopically transplanted into the pancreas of 129xC57Bl6 mice. 3 weeks after transplantation, the mice were treated according to the regimen. (**H**) Representative images of pancreatic tumors of transplanted TPAC mice treated according to the regimen, stained with H&E (blue/pink), p-paxillin (brown) and counterstained with haematoxilin (blue). (**I**) Diagrams showing the percentage of tumor area out of the total analyzed area of transplanted mice after treatment. (**J**) Diagrams showing the p-paxillin content as percentage of positively stained cells in relation to the total area. All results in graphs are shown as a mean value ± SEM. For the statistical analyses we used Mann-Whitney U test (**E**, **F**, **I**, and **J**), *t* test (**B** and **E**), and for the distribution, Shapiro-Wilk normality test (all panels). The *P* value ˂ 0.05 was considered to have significance. Scale bars: 20 μm.

In a next step, we investigated whether inhibition of the paxillin-Src-Erk signalosome has effects on cancer cell migration toward neurons and tumor severity. We used the small molecule inhibitor of paxillin protein disruptor, 6-B345TTQ, which inhibits the binding of paxillin to integrin4-α and regulates cell migration (40–42). As expected, we found a significant decrease in paxillin phosphorylation at Y118 in SU.86.86 and T3M4 cells treated with 6-B345TTQ by Western blots (Supplemental Figure 12, A and B). Ex vivo, we found that cells pretreated with 6-B345TTQ performed poorly in the migration assay toward DRG for every parameter examined: FMI (vehicle: 0.2136; 6-B345TTQ: 0.1469), speed (vehicle: 0.08442; 6-B345TTQ: 0.05425), and distance (vehicle: 42.08; 6-B345TTQ: 31.73) (Figure 6, A and B). We observed similar effects after pretreatment of cancer cells with the ERK1/2 phosphorylation inhibitor AZD8330 for 1 hour (Supplemental Figure 12D). The decreased phosphorylation of ERK1/2 in treated cells was verified by immunoblotting (Supplemental Figure 12, C and D).

Next, we tested the effects of paxillin inhibition with 6-B345TTQ on pancreatic cancer progression in vivo. To this end, we treated 5-month-old TPAC animals with 6-B345TTQ (1 mg/kg) 5 days per week for 4 weeks and analyzed the pancreas for tumor growth (Figure 6C). All animals developed tumors with comparable pancreatic weights in both groups (Supplemental Figure 13A). Remarkably, the tumor innervation, as measured through the PGP9.5 content, was decreased in the tumors of TPAC mice treated with 6-B345TTQ (mean 0.31% of total area) compared with control TPAC mice treated with DMSO (mean 0.64% of total area) (Figure 6, D and E). Moreover, a reduction in p-paxillin was detected in the pancreas of the treated TPAC mice (mean 4.72% of total area) compared with the DMSO controls (mean 9.79% of total area) (Figure 6, D and F). These results strongly suggested that blockade of paxillin phosphorylation leads to a rapid decrease in tumor innervation.

In addition, we tested the activity of highly proliferating, preselected in vitro clones from TPAC tumors by implanting them orthotopically into the pancreas of 8–10 week old WT mice (129xC57BL/6) (Figure 6G). We treated transplanted mice for 4 weeks with 6-B345TTQ (1 mg/kg) by applying it for 5 days weekly and treated the control animals with DMSO (Figure 6G). We did not detect any changes in body weight of recipient animals transplanted with TPAC cancer cells (Supplemental Figure 13B). Interestingly, pancreatic weight of recipients implanted with TPAC cells decreased after treatment with 6B345TTQ (0.138 g) compared with DMSO (0.168 g) (Supplemental Figure 13C) due to strong reduction of the primary tumor area in mice treated with 6B345TTQ (0.95% of total area) compared with DMSO-treated controls (3.43% of total area) (Figure 6, H and I). Furthermore, we identified a strong reduction of paxillin phosphorylation in cancer cells of recipients treated with 6B345TTQ (4.72% of total area) compared with control mice treated with DMSO (9.79% of total area) (Figure 6, H and J). These results confirmed paxillin phosphorylation as a key actor in tumor progression and a promising target for therapeutic approaches.

Finally, we interrogated whether TGF-α, which is the prime driver of carcinogenesis in the neuro-invasive TPAC and TPC genotypes, is also prognostically relevant in human PDAC. Analysis of survival data from the TCGA database uncovered TGF-α as a very strong prognostic factor in human PDAC. Here, PDAC patients with a low level of TGF-α expression had a remarkably high 5-year survival rate of 60%, whereas the 5-year survival rate dropped to 18% for patients with high intratumoral TGF-α content (The Protein Atlas database: https://www.proteinatlas.org/ENSG00000163235-TGFA/pathology/pancreatic+cancer). We also explored whether the tissue expression levels of *Tgf-α* correlate with the emergence of NI in human PDAC. For this purpose, we scrutinized the TCGA for information on the PNI status of the patients with PDAC. The metadata and data repository of the TCGA-PDAC database includes digitized pathology reports that included the information on the PNI status for 159 samples, 134 of which had been reported to show PNI, and 25 were described as non-PNI. This way, we could compare expression of TGF-α in the PNI versus non-PNI groups and discovered a significantly higher expression of TGF-α in the PNI group, compared to the non-PNI group (Supplemental Figure 14C). There was no correlation between the PNI status and the tumor tissue bulk RNA expression levels of *CCl2, CCR4,* or *paxillin* (Supplemental Figure 14C).

## Discussion

NI is an extremely frequent mode of tumor spread in human PDAC, and its severity is a clinching determinant of prognosis (9, 15, 16). However, NI is not sufficiently modeled by the commonly used, oncogenic Kras-driven GEMMs of PDAC, as they do not exhibit the extent of the typical PNI of human PDAC. In this study, we performed what is, to our knowledge, the largest phenotypic screen of GEMMs of PDAC to date for detecting models with genuine PNI. This way, we found that mutants that overexpressed TGF-α, i.e., the TPAC and TPC GEMMs, harbored human-like PNI. In the Kras-based mutants, on the other hand, the cancer cells were present in close proximity to the nerves, but this occurred more or less sporadically. Functionally, cancer cells isolated from the primary tumors of TGF-α–driven GEMM mice showed higher neuroaffinity and induced stronger neuritogenesis than oncogenic Kras-based models, possibly due to increased secretion of neurogenic/neurotrophic factors. We also show that TGF-α promotes the secretion of CCL2 from DRG neurons, which, in turn, activates the cancer cell cytoskeleton through CCR4, paxillin phosphorylation, and cell protrusion formation. Targeting of the CCR4-phospho-paxillin axis was sufficient to reduce tumor progression and neuro-affinity of cancer cells in vivo.

Our study has several implications. First, we provide examples for GEMM of PDAC that are more similar to human PDAC with respect to PNI. Although oncogenic Kras-driven PDAC also exhibits a rich innervation and close spatial relation between nerves and cancer cells in the primary tumor, the typical perineural lining of cancer cells, as seen in human PDAC, was not as prominent and frequent as in the TPAC model. We believe that GEMM like TPAC can serve as valuable tools for future studies that aim to analyze mechanisms of NI in PDAC and for preclinical trials that aim to target NI. One of first events in the emergence of NI in PDAC is SC activation by preinvasive lesions, i.e., PanIN lesions (14, 43, 44). On the other hand, once fully transformed, invasive cancer cells seem to upregulate TGF-α, which augments NI over induction of CCL2 secretion from neurons, which acts upon CCR4 on cancer cells. The gradual increase of TGF-α levels from KC to the KPC, TPC, and TPAC mice hence supports the notion that TGF-α aggravates the perineurally invasive phenotype of pancreatic cancer.

Second, our observations imply that, despite anatomic differences between the murine and human pancreas, neuropathic phenomena like NI are reproducible in GEMMs of PDAC. The human pancreas is a compact organ in the retroperitoneum, whereas the murine pancreas is less demarcated, quite diffusely distributed in the mesentery, and is therefore termed an intraperitoneal, mesenteric type pancreas (45). Furthermore, nerve trunks in the human pancreas are widely distributed over the parenchyma and present both within and around the lobes (45). In contrast, the normal mouse pancreas contains nerve trunks only around extralobular vessels and in the vicinity of lymphoid sites (45). In the light of this knowledge, it was interesting for us to detect enlarged nerves and PNI in the tumor core that was embedded in the lobes of the pancreas. Hence, the genetic features of GEMMs are sufficient to overcome the anatomic differences between the human and murine pancreas and to generate human-like neuro-phenotypes.

Third, our study points out a mechanism that generates human-like NI. NI is believed to be initiated and orchestrated by chemotactic factors released from neurons that chemoattract cancer cells (31, 46). In our study, we screened for chemokines that were increasingly secreted from DRG neurons when confronted with cancer cells and detected increased production of CCL2. As the TGF-α–based GEMMs harbored human-like neural invasion, we explored and found that TGF-α can induce CCL2 expression in DRG neurons. In studies with prostate cancer, which is another prime example for a neuroinvasive cancer, He et al. demonstrated that DRG neurons express CCL2, which mediated the nerve-derived migration capacity of cancer cells (29). NI was significantly hampered in migration assays using DRG neurons isolated from CCL2^–/–^ mice (29). Moreover, we show a previously unknown link between TGF-α, Ccl2, and NI in pancreatic cancer, using the herein reported, human-like neuroinvasive TPAC model and human data. Here, we also show that invaded nerves in human PDAC contain visibly higher amounts of CCL2 than noninvaded nerves. Although chemokines and neurotrophic factors with chemoattractive properties such as NGF, GDNF, artemin, or neurturin have long been studied in the context of NI in PDAC (31, 47–50), the cytoskeletal adaptations of cancer cells during their nerve-directed migration have not been in the focus. We detected a 4.03-fold overexpression of the high-affinity NGF receptor TrkA (*Ngfr*). In addition, we found a 2.53-fold overexpression of *Gdnf* in the TPAC cancer cells compared to KPC cancer cells. It should be noted that, in the present study, the expression of neurotrophic and neurogenic factors (like *Gdnf*) was compared between the TPAC and KPC genotypes. Neurotrophic factors that we found to be overexpressed (like GDNF) are thus likely to be even more prominently upregulated when compared with the healthy normal pancreas. In addition to this classical neurotrophic molecule, we demonstrated that CCL2 derived from DRG neurons activates the cancer cell cytoskeleton through the CCR4 receptor and the entailing paxillin phosphorylation. Paxillin is one of the major components of focal adhesions and is involved in the transmission of signals, regulation of cell morphology, and control of cell spreading and migration (51) Recent studies have shown that phosphorylation of paxillin by FAK or SRC at Tyr118 and Ser178 is necessary for the stimulation of cancer cell migration. (52) Hence, we provide a mechanistic explanation for the enhanced motility of cancer cells at the cytoskeletal level when chemoattracted by neuron-derived chemokines. Cytoskeletal adaptations in pancreatic cancer have recently also been shown to be induced by SCs, which, by applying forces on cancer cells, were shown to alter the cancer cell cytoskeleton and promote the migration capacity of cancer cells (53).

Interestingly, administration of rCCL2 resulted in increased nerve density in the murine pancreas, which was reversible upon blockade of the CCR4 receptor. This suggests that the CCL2/CCR4 signaling pathway affects not only the chemoattraction and the migration machinery of cancer cells, but additionally modulates the neural ingrowth and innervation of cancer.

Our findings also underline some clinically relevant aspects of the identified molecular players. We found TGF-α content of the primary tumor, and p-paxillin in cancer cells within samples from patients with PDAC to be unfavorable prognostic markers for overall survival. Furthermore, inhibition of paxillin phosphorylation with the inhibitor 6-B345TTQ significantly reduced nerve density and tumor size in TPAC mice and cancer-nerve affinity in KPC mice. We propose that interference with the TGF-α-CCL2-paxillin axis holds potential for prognostic improvement and should be considered in upcoming clinical trials.

Our analyses of single cell RNA-Seq expression data of human PDAC from publicly available sources showed cancer cells as the most prominent source of TGF-α in human PDAC tissues. Other cell types like myeloid cells or endocrine cells also turned out to exhibit TGF-α expression that was, however, much lower when compared to the TGF-α expression levels in cancer cells. Therefore, although we cannot exclude a similar effect of TGF-α derived from such noncancerous cells, cancer cell–derived TGF-α is most likely to generate the observed effects.

We also questioned the genetic similarity of the TPAC model to human PDAC when we saw the presence of nerve invasion but the lack of any Kras mutation in the TPAC model. However, based on our results, TGF-α overexpression seems to be a typical feature of human PDAC, as shown in the TCGA database, although we cannot know for sure how relevant this overexpression is in the earliest phases of human pancreatic cancer. We believe that the solution to this seeming discrepancy lies in the fact that the TGF-α-EGFR axis activates those pathways that have been repeatedly shown to be overrepresented and hyperactive in RNA-Seq studies of human PDAC, such as Ras-Raf-MAPK and PI3K-Akt-mTOR signaling. Indeed, we could show here a higher Ras activity in TPAC cancer cells compared to that in KPC cancer cells. As such, TGF-α overexpression imitates the signaling events induced by mutant (oncogenic) Kras, as known from human PDAC as well as from the KPC model. This is the most likely reason why the *Ela1-TGF-α* mouse model was one of the earliest autochthonous murine transgenic models of PDAC (54).

It should be noted that in our study, not all TPAC or TPC mice exhibited PNI (PNI: 60% of TPAC mice), which suggests the presence of tumor heterogeneity in animals of identical genotype. Genomic sequencing of outlier animals of the same genotype may here provide insight into further genetic alterations that are necessary for a human-like neuroinvasive phenotype.

In conclusion, the present study demonstrates the power of large-scale phenotype screens for identifying GEMMs of PDAC with human-like neuropathic characteristics. Our TPAC mouse model of NI in PDAC represents a fundamental tool for studying the molecular mechanisms behind the cancer-neuron cross talk. Moreover, the TGF-α-CCL2-CCR4-p-paxillin axis represents an NI-inducing, actionable axis that deserves further testing in preclinical and clinical settings for prognostic improvement.

## Methods

For further details on methods, please refer to the Supplemental Methods.

### Mice.

We used GEMM that are available in the repository of the CRC1321 in Munich, “Modeling and Targeting Pancreatic Cancer”. The CRC1321 has generated, cataloged, and characterized 295 different allele combinations of oncogenes and tumor suppressors in GEMMs of PDACs over the past 4 years. For a full list of the genotypes, please refer to Supplemental Table 1. For the list of the analyzed age ranges and the number of mice per genotype, please refer to Supplemental Table 2. The following abbreviations have been used for the genotypes of particular interest for this study: TPAC (*Ela1-Tgf-*α*; p48-Cre; trp53^lox/lox^*; *RelA^lox/lox^*), TPC (*Ela1-Tgf-*α*; p48-Cre; trp53^lox/lox^*), KPC (*p48-Cre*; *LSL-Kras^G12D^; trp53^lox/lox^*), and KC (*p48-Cre*; *LSL-Kras^G12D^*). For orthotopic and i.v. transplantations, we used 129X57BL/6 and C57BL/6N WT mice. All animals were housed in IVC cages under SOPF conditions at ZPF, Klinikum rechts der Isar, Technical University of Munich.

### Patient samples.

Tumor samples were collected from 59 patients with pancreatic cancer who underwent resection at the Department of Surgery at the Technical University of Munich. Tissues were fixed in 4% paraformaldehyde in 1 × PBS overnight and embedded in paraffin. Thin cuts of 2 mm were prepared using a rotary microtome (HM355S, Thermo Fisher Scientific). Patients were grouped into p-paxillin low-expression group (p-paxillin expression/paxillin expression) and p-paxillin high expression group (p-paxillin expression/paxillin expression) based on immunostaining density. The survival data of the patients in The Cancer Genome Atlas (TCGA) was extracted from the following link: https://www.proteinatlas.org/ENSG00000163235-TGFA/pathology/pancreatic+cancer For comparison of the survival of the high versus low expression groups, the median expression was chosen as cutoff based on the following information provided by the TCGA: the survival data in the TCGA database were retrieved using the median expression based on the FKPM (number Fragments Per Kilobase of exon per Million reads) value calculated based on the gene expression data from all patients in the data set.

### Statistics.

All results in graphs are shown as mean ± SEM. For the statistical analyses, we used 2-tailed *t* tests, Mann-Whitney tests, ordinary 1-way ANOVAs, Dunnett’s multiple comparisons tests and, for the distribution, Shapiro-Wilk normality tests using GraphPad Prism 5. *P* ˂ 0.05 was considered significant.

### Study approval.

All animal experiments were approved by the governmental commission for animal protection of the Government of Upper Bavaria (Regierung von Oberbayern, no. 55.2-2532-Vet-02-16-165). The study was approved by the ethics committee of the Technical University of Munich (589/19s).

### Data availability.

The microarray data are publicly available at the Gene Expression Omnibus (GEO) with the accession number GSE201994 (https://www.ncbi.nlm.nih.gov/geo/query/acc.cgi?acc=GSE201994). The spatial transcriptomics data are publicly available at the Gene Expression Omnibus (GEO) with the accession number GSE245126 (https://www.ncbi.nlm.nih.gov/geo/query/acc.cgi?acc=GSE245126). All primary data are available as Supplemental Material online as uploaded on the journal website. The full, uncut gels of the immunoblots have been provided in the online Supplemental Material.

## Author contributions

IED, GOC, and RI designed the study. XW, LY, ST, RI, LR, RÖ, CJ, KÇ, CMR, ÜY, IHG, QL, MS, SEY, OUS, SÇ, and GS performed the experiments and/or analyzed the data. ML supported the IF and analysis and provided substantial intellectual input regarding the 3D-Migration assay. KND, KG, ML, and HA supported the transplantation experiments and generated the TPAC model; BV and MK performed bioinformatical analysis of RNA-Seq data; KS and AM assisted in the histopathological analyses; XW, RI, and IED wrote manuscript. MR, AK, and RR critically read and reviewed the manuscript. DS and SB provided the majority of the mouse models. HF, RMS, GOC, and IED supervised the study. All authors have approved the final version of the manuscript. The order of shared first authorship is based on equal contribution and based on temporal sequence of contribution.

## Supplementary Material

Supplemental data

Supporting data values

## Version 1 08/22/2023

In-Press Preview

## Version 2 11/01/2023

Electronic publication

## Version 3 11/13/2023

change en-dash to hyphen in phrase TGF-a-CCL2-paxillin, page 15

## Version 4 04/30/2024

Corrected affiliation no. 6
