# Supplementary material for: Phenotype screens of murine pancreatic cancer identify a Tgf-α-Ccl2-paxillin axis driving human-like neural invasion
Source: J Clin Invest. 2023 Aug 22;133(21):e166333. doi: 10.1172/JCI166333 (PMC10617783; doi:10.1172/JCI166333)
Supplement: Supplemental data [file jci-133-166333-s007.pdf]

Figure S1

A

Line	Genotype	Nr	Average life span	Disease
KC	Kras$^{+/LSLG12D}$;Ptf1$^{+/CRE}$	10	275 days	PanIN lesions at 4-5 moths
KPC	Kras$^{+/LSLG12D}$;Ptf1$^{+/CRE}$; Trp53$^{+/fl}$	10	61.5 days	PDAC
KPAC	Kras$^{+/LSLG12D}$;Ptf1$^{+/CRE}$; Trp53$^{+/fl}$;p65$^{fl/fl}$	10	82 days	PDAC
TPC	Ela-TGFa^{tg};Ptf1$^{+/CRE}$; Trp53$^{fl/fl}$	22	297 days	9.1 % with PDAC
TPAC	Ela-TGFa^{tg};Ptf1$^{+/CRE}$; Trp53$^{fl/fl}$; p65$^{fl/fl}$	24	370 days	75 % with PDAC

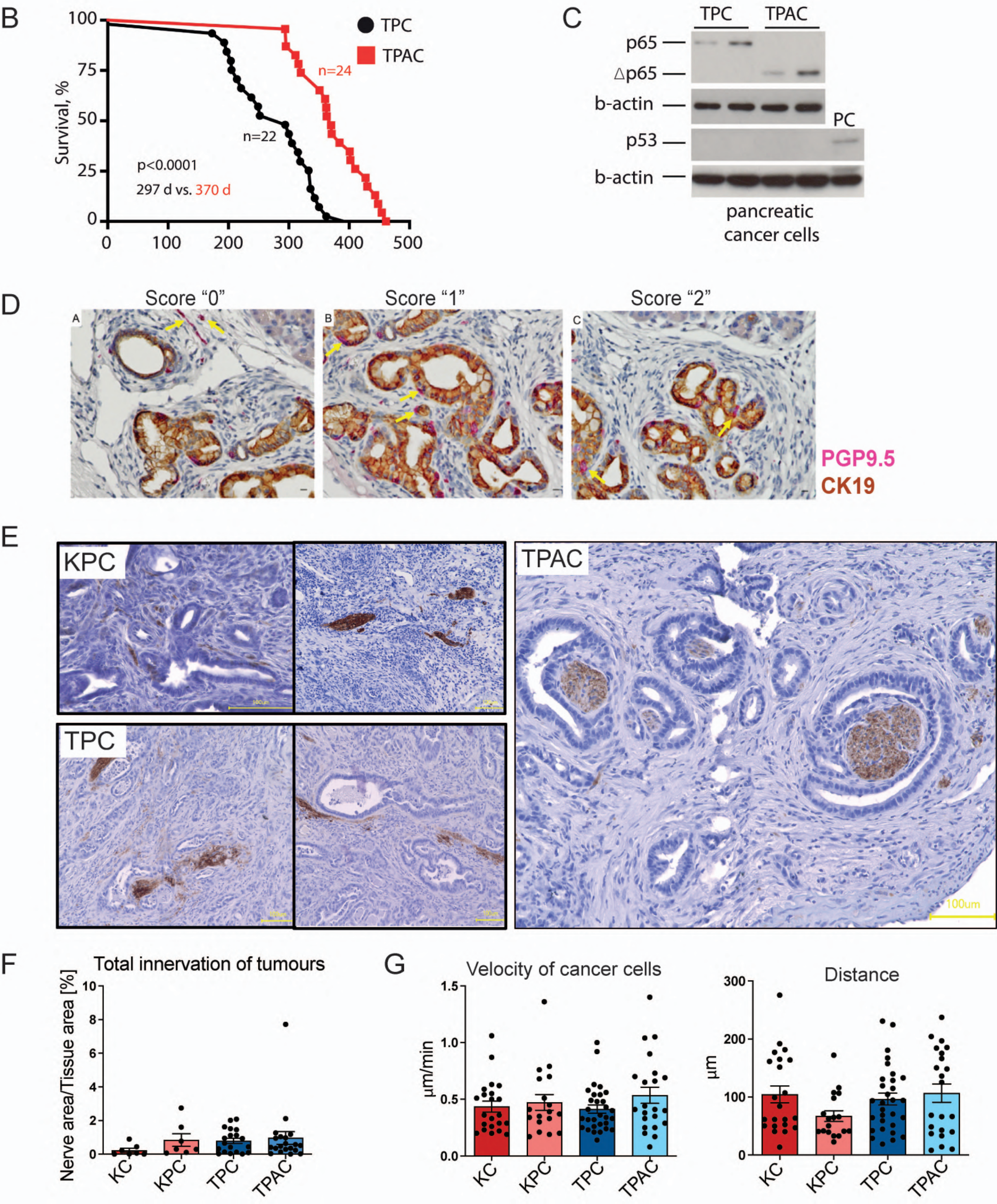

Figure S2

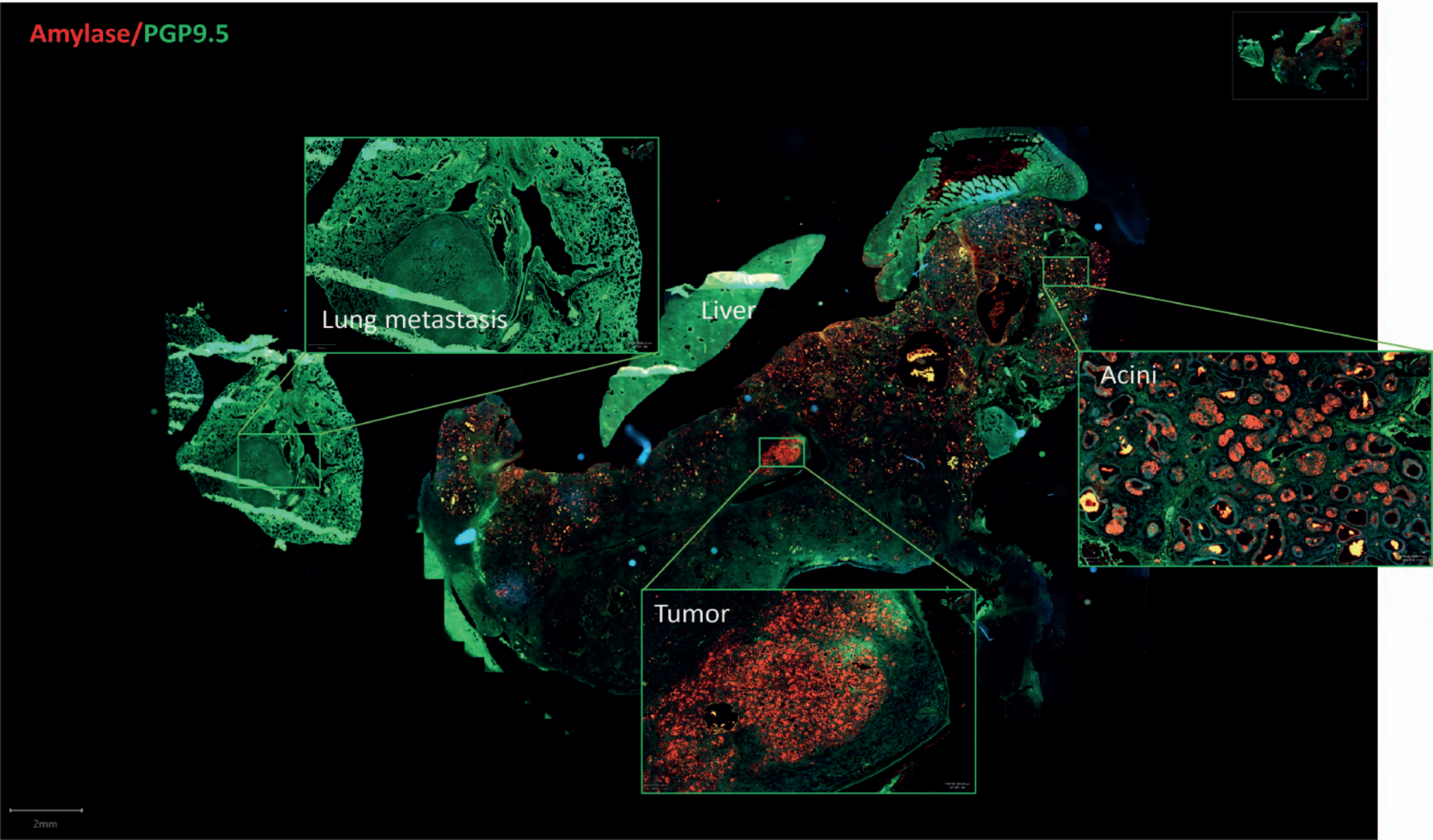

Figure S3

TPAC vs. TPC

A

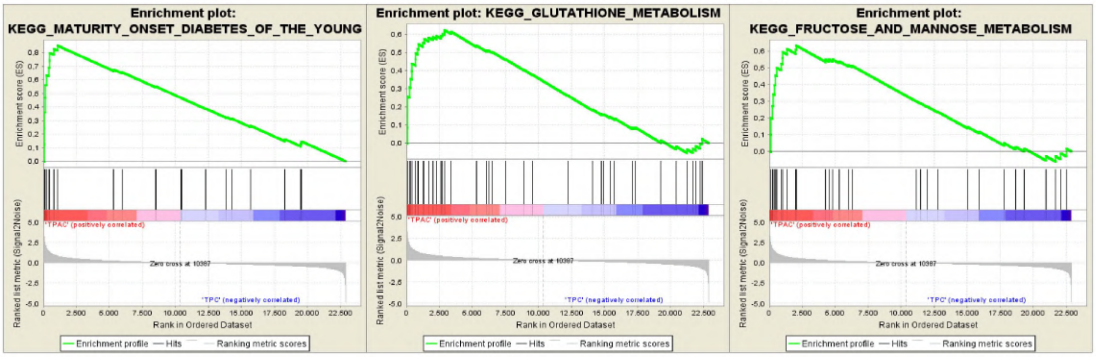

B

	NAME	SIZE	ES	NES	NOM p-val	FDR q-val	FWER p-val	RANK AT MAX
1	KEGG_MATURITY_ONSET_DIABETES_OF_THE_YOUNG	24	0.85073555	2.4092388	0	0	0	1051
2	KEGG_GLUTATHIONE_METABOLISM	44	0.62253064	2.0181694	0	0.003468067	0.007	2901
3	KEGG_FRUCTOSE_AND_MANNOSE_METABOLISM	32	0.63197446	1.8948294	0.001858736	0.02153301	0.061	2046
4	KEGG_HISTIDINE_METABOLISM	26	0.64063203	1.8274286	0	0.03441649	0.128	2921
5	KEGG_SPHINGOLIPID_METABOLISM	38	0.57190394	1.8074318	0.001788909	0.034419093	0.157	3525
6	KEGG_STEROID_HORMONE_BIOSYNTHESIS	25	0.6440711	1.8046125	0.007130125	0.02985083	0.164	3018
7	KEGG_HEMATOPOIETIC_CELL_LINEAGE	65	0.5197438	1.7720928	0.001683502	0.037837602	0.238	1310
8	KEGG_METABOLISM_OF_XENOBIOTICS_BY_CYTOCHROME_P450	29	0.59025455	1.7363237	0.008849558	0.048785776	0.324	3251
9	KEGG_PENTOSE_PHOSPHATE_PATHWAY	24	0.6145124	1.7222588	0.005309735	0.051345617	0.373	2644
10	KEGG_TRYPTOPHAN_METABOLISM	35	0.5676878	1.7196183	0.003521127	0.04690276	0.378	3647
11	KEGG_LEUKOCYTE_TRANSENDOTHELIAL_MIGRATION	108	0.45528454	1.6962656	0	0.053437743	0.447	2150
12	KEGG_TIGHT_JUNCTION	115	0.4466766	1.6917872	0.00166113	0.051120717	0.462	1477
13	KEGG_CELL_ADHESION_MOLECULES_CAMS	105	0.4465153	1.6709884	0.003149606	0.057317026	0.533	1239
14	KEGG_O_GLYCAN_BIOSYNTHESIS	28	0.5693942	1.664826	0.009469697	0.056478947	0.552	1030
15	KEGG_DRUG_METABOLISM_CYTOCHROME_P450	33	0.535267	1.6389766	0.003442341	0.065786235	0.635	2729
16	KEGG_GLYCOLYSIS_GLUCONEOGENESIS	52	0.4945406	1.6271468	0.010186757	0.06751881	0.668	1523
17	KEGG_RIBOSOME	68	0.4578669	1.6036876	0.007130125	0.07976694	0.743	6875
18	KEGG_NICOTINATE_AND_NICOTINAMIDE_METABOLISM	23	0.57636243	1.6033872	0.014260249	0.07538688	0.744	2064
19	KEGG_INOSITOL_PHOSPHATE_METABOLISM	52	0.47702157	1.5561275	0.010016695	0.10421144	0.848	3441
20	KEGG_TERPENOID_BACKBONE_BIOSYNTHESIS	15	0.60388243	1.5507737	0.03250478	0.10315168	0.862	3466
21	KEGG_PEROXISOME	70	0.4472005	1.5393016	0.016638935	0.107746236	0.885	2823
22	KEGG_PATHOGENIC_ESCHERICHIA_COLI_INFECTION	43	0.4784825	1.5223991	0.026929982	0.116578475	0.911	3431
23	KEGG_LYSINE_DEGRADATION	41	0.47991666	1.5068485	0.017857144	0.127734	0.937	5217
24	KEGG_OLFACTORY_TRANSDUCTION	25	0.5097665	1.4775319	0.05283019	0.15294166	0.971	1084
25	KEGG_N_GLYCAN_BIOSYNTHESIS	41	0.4692046	1.4749271	0.044483986	0.1497725	0.972	2558
26	KEGG_JAK_STAT_SIGNALING_PATHWAY	134	0.38527736	1.459002	0.013846153	0.16086785	0.979	2134
27	KEGG_ABC_TRANSPORTERS	40	0.45518944	1.4366304	0.041666668	0.18090415	0.987	1664
28	KEGG_WNT_SIGNALING_PATHWAY	135	0.36600208	1.4006691	0.02511774	0.22057502	0.996	3306

C

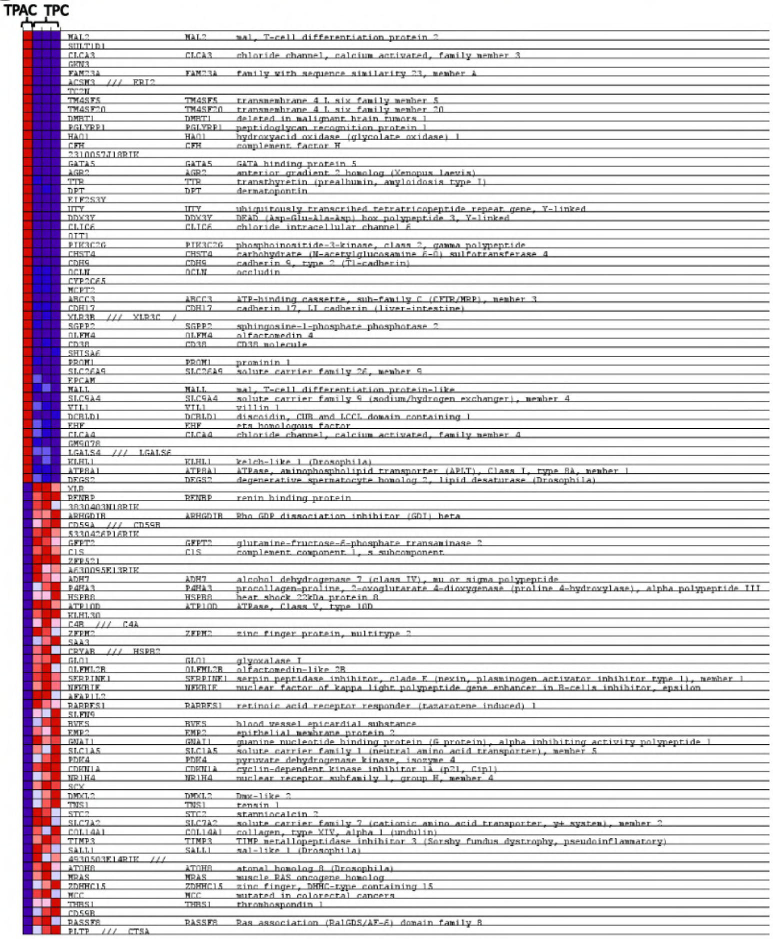

D

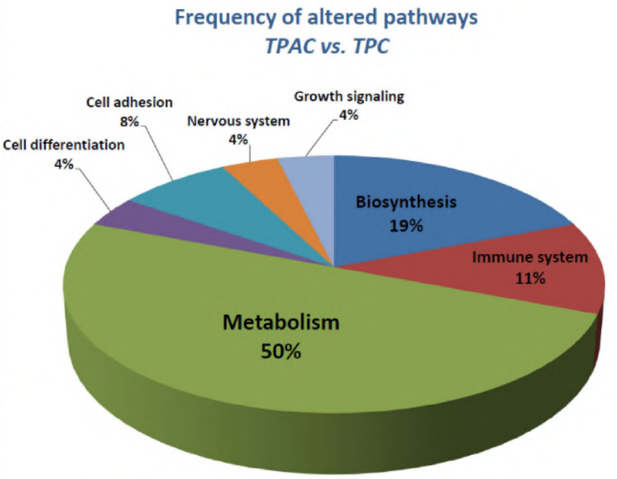

- 100 / 171 gene sets are upregulated in phenotype **TPAC**
- 28 gene sets are significant at FDR < 25%
- 16 gene sets are significantly enriched at nominal pvalue < 1%
- 27 gene sets are significantly enriched at nominal pvalue < 5%

Figure S4

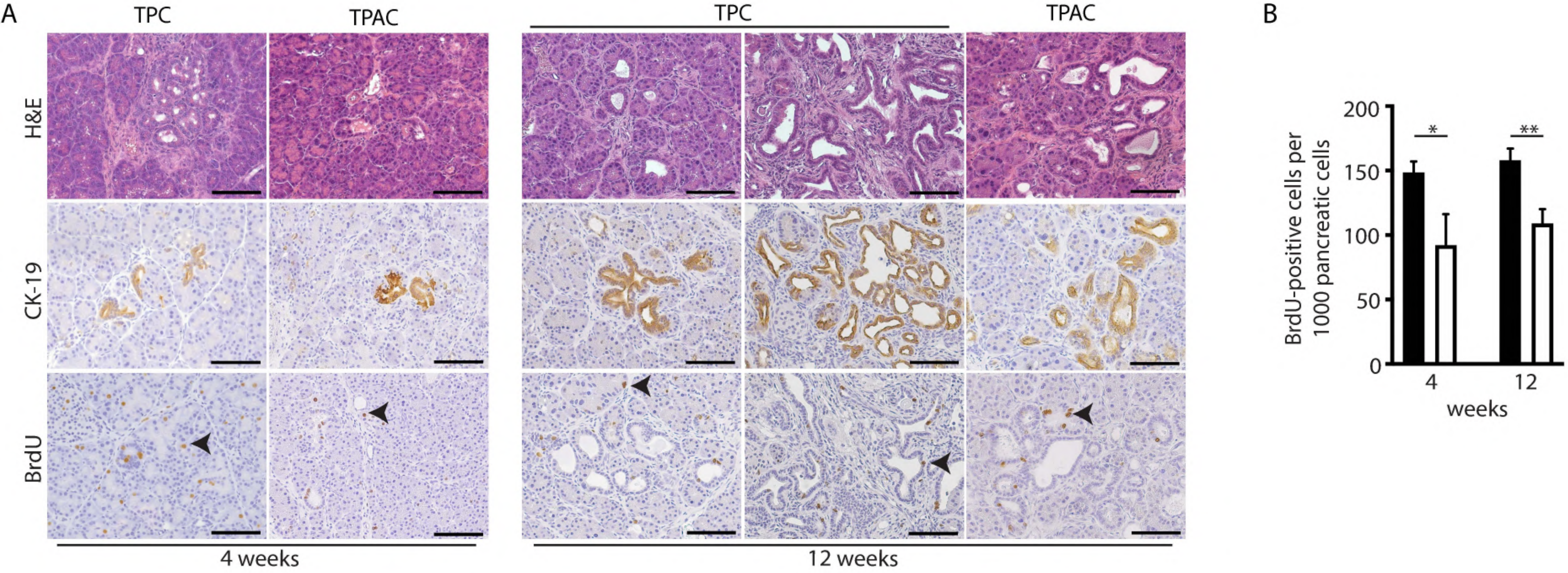

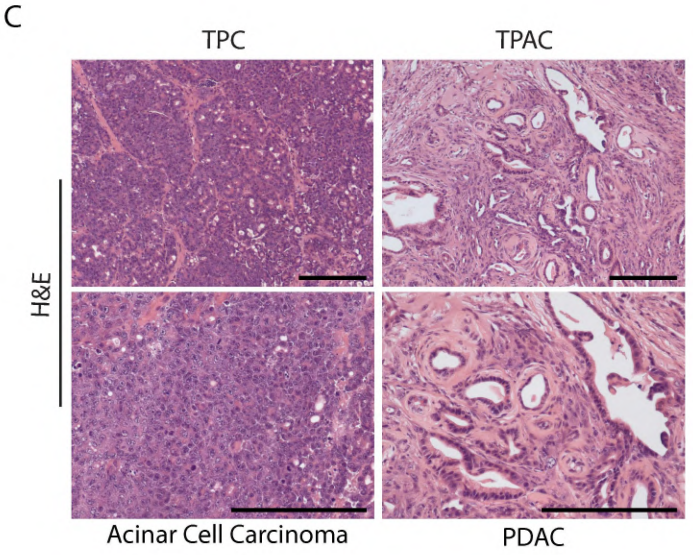

D

	acinar cell carcinoma	PDAC	mixed	Metastases	Total
TPC	17	2	3	7	22
TPAC	5	18	1	2	24

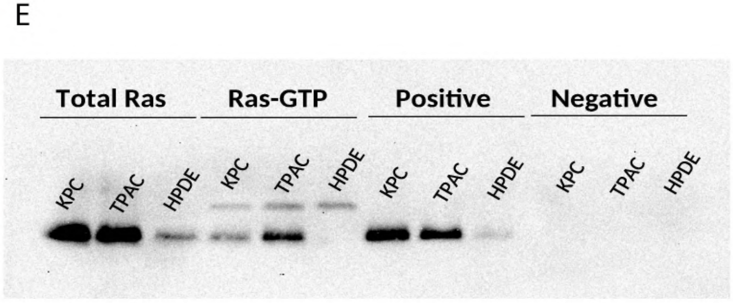

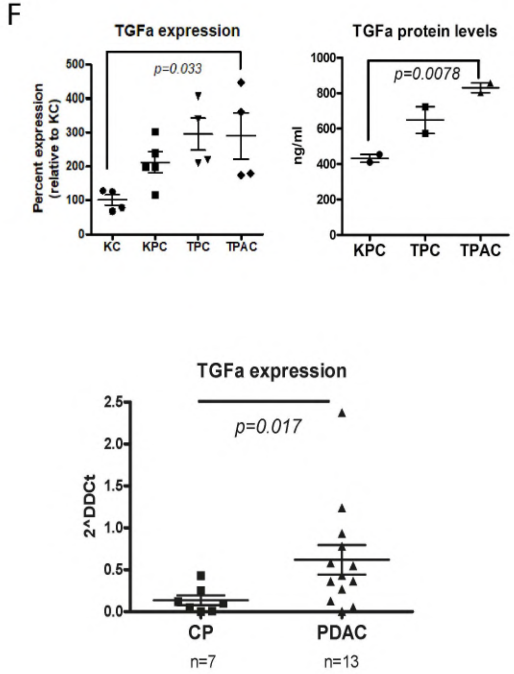

Figure S5

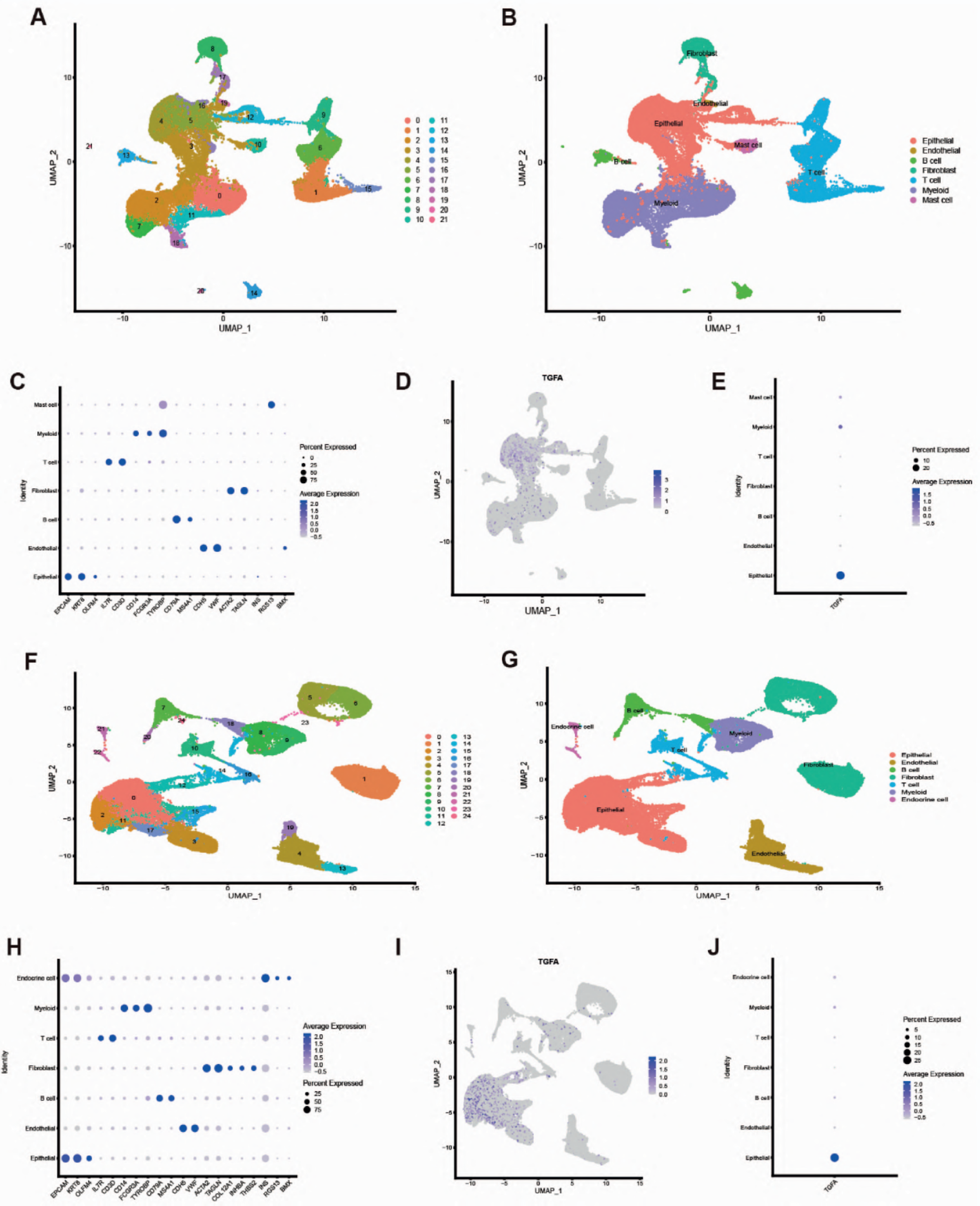
A
B
C
D
E
F
G
H
I
J
UMAP_1
UMAP_2
Epithelial
Endothelial
B cell
Fibroblast
T cell
Myeloid
Mast cell
Endocrine cell
Percent Expressed
Average Expression
Identity
TGFA
EPCAM
KRT8
OLFM4
IL7R
CD3D
CD14
FCGR3A
TYROBP
CD79A
MS4A1
CDH5
VWF
ACTA2
TAGLN
COL12A1
INHBA
THBS2
INS
RGS13
BMX

Figure S6

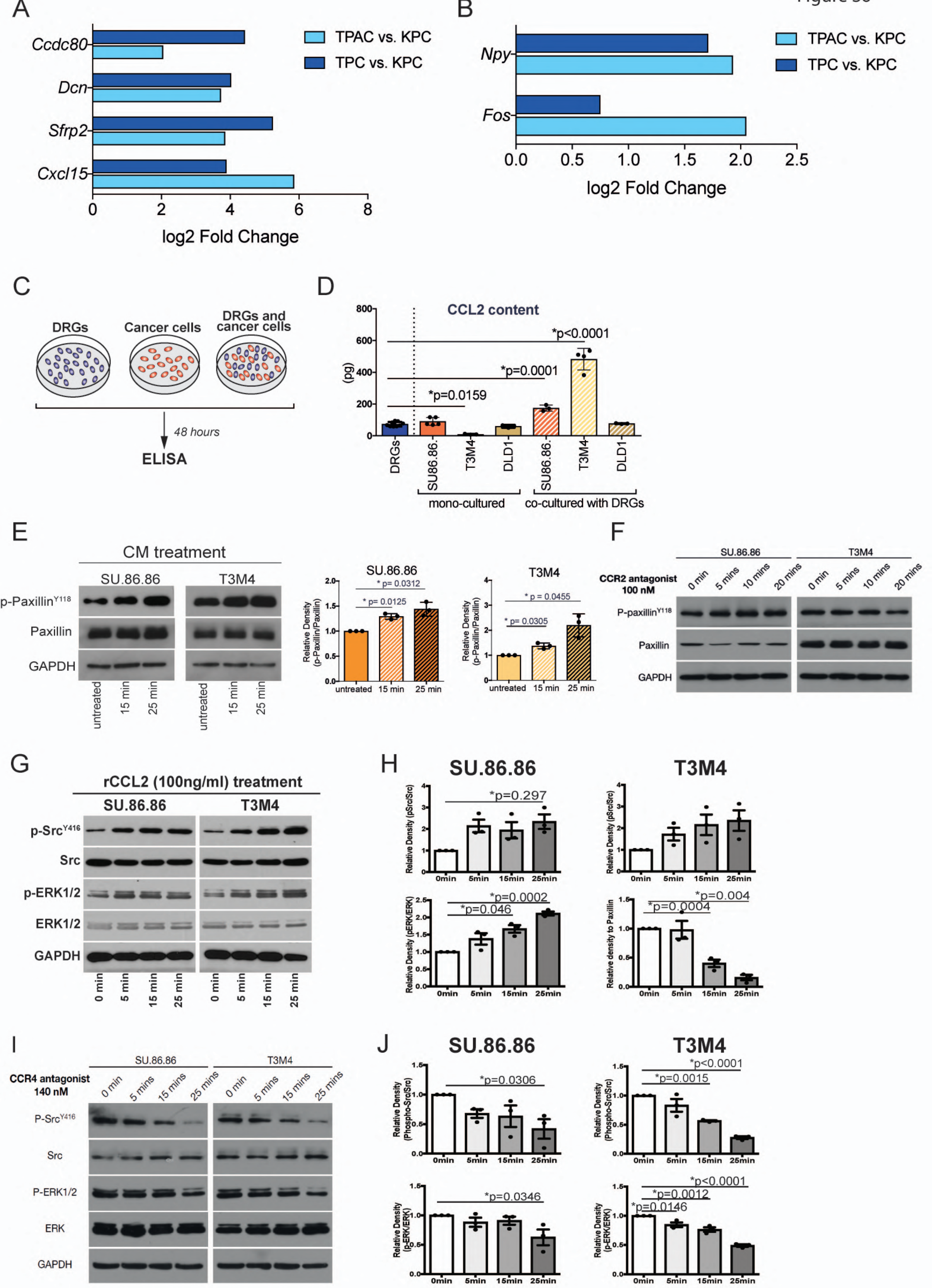
A
Ccdc80
Dcn
Sfrp2
Cxcl15
TPAC vs. KPC
TPC vs. KPC
log2 Fold Change
B
Npy
Fos
log2 Fold Change
C
DRGs
Cancer cells
DRGs and cancer cells
48 hours
ELISA
D
CCL2 content
(pg)
*p<0.0001
*p=0.0001
*p=0.0159
DRGs
SU86.86
T3M4
DLD1
mono-cultured
co-cultured with DRGs
E
CM treatment
SU.86.86
T3M4
p-Paxillin Y118
Paxillin
GAPDH
untreated
15 min
25 min
Relative Density (p-Paxillin/Paxillin)
* p= 0.0312
* p= 0.0125
* p = 0.0455
* p= 0.0305
F
CCR2 antagonist 100 nM
0 min
5 mins
10 mins
20 mins
P-paxillin Y118
Paxillin
GAPDH
G
rCCL2 (100ng/ml) treatment
SU.86.86
T3M4
p-Src Y416
Src
p-ERK1/2
ERK1/2
GAPDH
0 min
5 min
15 min
25 min
H
SU.86.86
T3M4
Relative Density (pSrc/Src)
*p=0.297
Relative Density (pERK/ERK)
*p=0.0002
*p=0.046
Relative density to Paxillin
*p=0.004
*p=0.0004
0min
5min
15min
25min
I
SU.86.86
T3M4
CCR4 antagonist 140 nM
0 min
5 mins
15 mins
25 mins
P-Src Y416
Src
P-ERK1/2
ERK
GAPDH
J
SU.86.86
T3M4
Relative Density (Phospho-Src/Src)
*p=0.0306
*p<0.0001
*p=0.0015
Relative Density (p-ERK/ERK)
*p=0.0346
*p<0.0001
*p=0.0012
*p=0.0146

Figure S7

A

Negative:0, weak positive:1, medium positive:2 strong positive:3

Acinus	Ductal (tumor)	neuron	Immune cells	fibroblast	Patients
1	0	1	0	1	M212
0	1	1	0	1	M235
2	1	2	1	2	M250
1	0	2	0	2	M272
1	1	2	1	1	M300
1	0	2	0	1	M301
1	0	2	0	2	M303
1	0	3	0	1	M312
0	1	1	0	0	M350
0	0	3	0	0	M393
0	0	1	0	0	M732
1	1	2	0	1	M758
0	0	2	0	0	M987
0	0	2	0	1	M1396

Acinus	Ductal (tumor)	neuron	Immune cells	fibroblast	TPAC
0	0	1	0	0	9087
0	1	1	0	0	14807
0	0	1	0	0	18538
0	0	1	1	0	18394
0	0	1	0	1	16090
0	0	0	0	1	18563
0	0	1	1	0	50063
0	0	1	0	0	54500
0	0	1	0	0	9037
0	0	1	0	0	9086

B

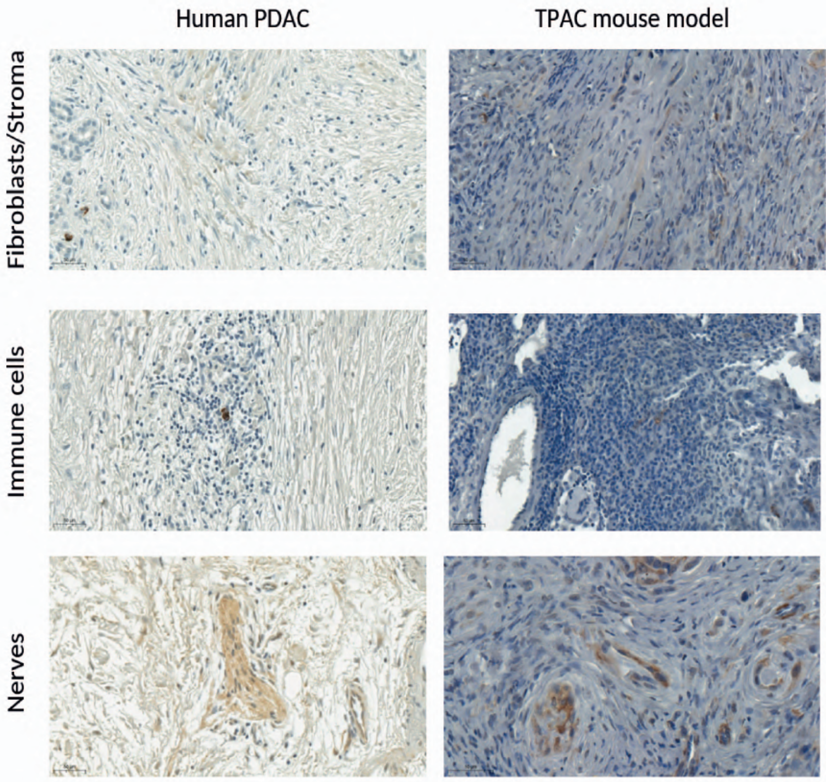

C

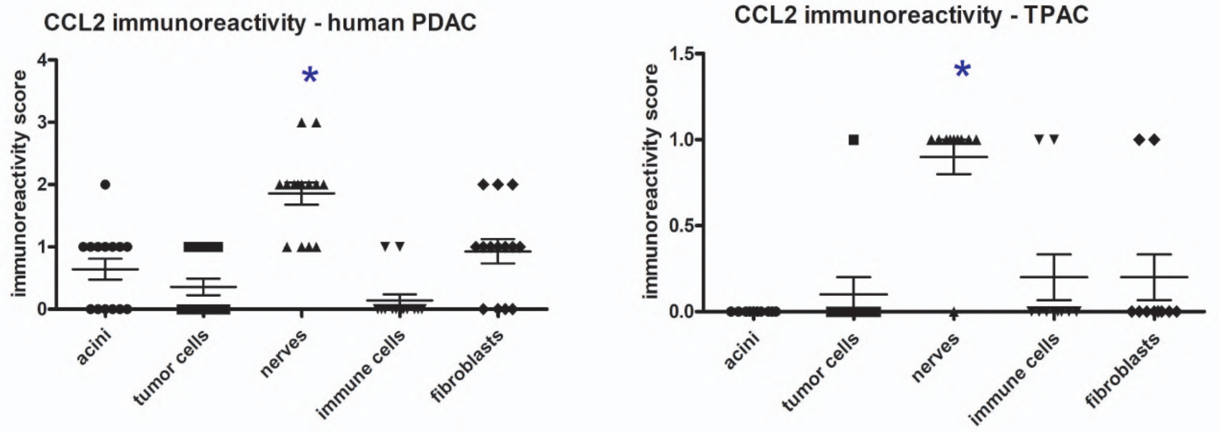

D

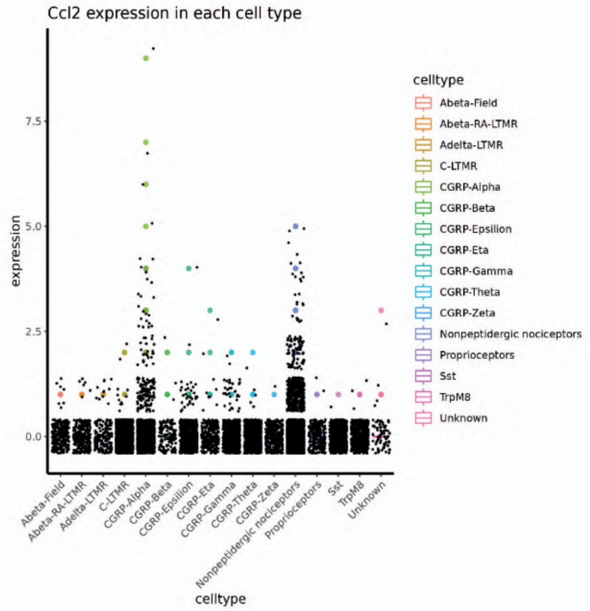

E

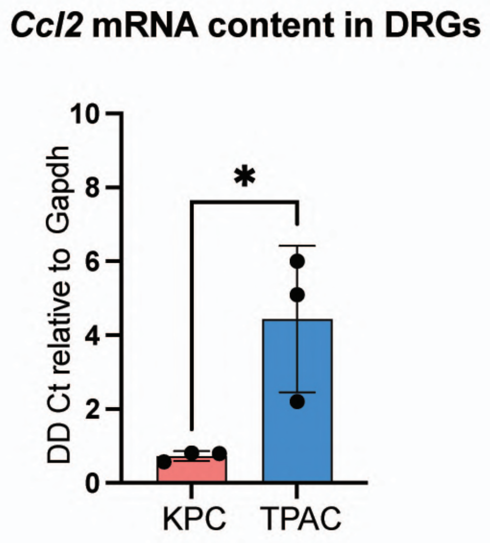

Figure S8

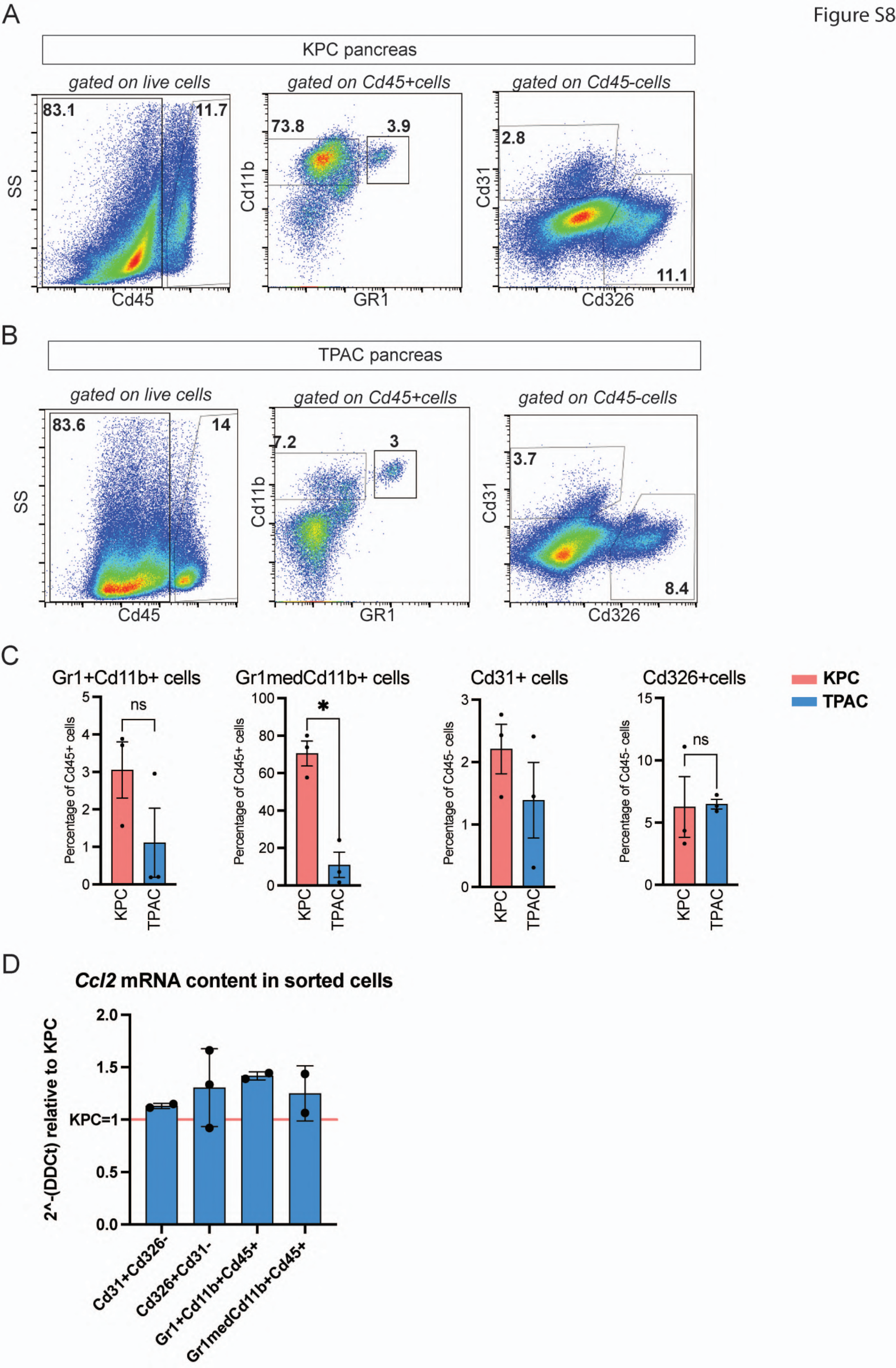
A
KPC pancreas
gated on live cells
83.1
11.7
SS
Cd45
gated on Cd45+cells
73.8
3.9
Cd11b
GR1
gated on Cd45-cells
2.8
11.1
Cd31
Cd326
B
TPAC pancreas
gated on live cells
83.6
14
SS
Cd45
gated on Cd45+cells
7.2
3
Cd11b
GR1
gated on Cd45-cells
3.7
8.4
Cd31
Cd326
C
Gr1+Cd11b+ cells
ns
Percentage of Cd45+ cells
Gr1medCd11b+ cells
*
Percentage of Cd45+ cells
Cd31+ cells
Percentage of Cd45- cells
Cd326+cells
ns
Percentage of Cd45- cells
KPC
TPAC
D
Ccl2 mRNA content in sorted cells
2^-(DDCt) relative to KPC
KPC=1
Cd31+Cd326-
Cd326+Cd31-
Gr1+Cd11b+Cd45+
Gr1medCd11b+Cd45+

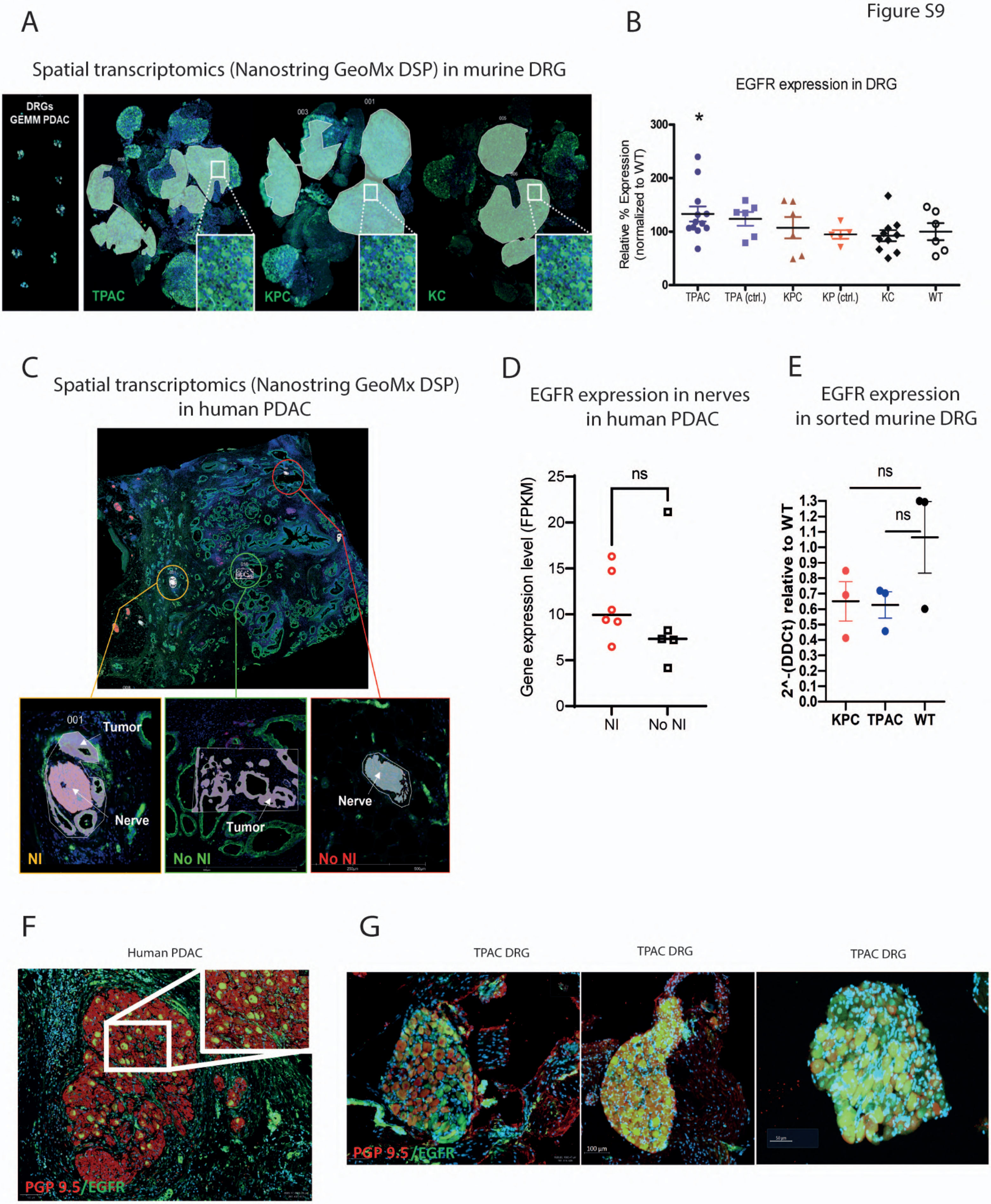

Figure S9
A
Spatial transcriptomics (Nanostring GeoMx DSP) in murine DRG
DRGs GEMM PDAC
TPAC
KPC
KC
B
EGFR expression in DRG
Relative % Expression (normalized to WT)
300
200
100
0
*
TPAC
TPA (ctrl.)
KPC
KP (ctrl.)
KC
WT
C
Spatial transcriptomics (Nanostring GeoMx DSP) in human PDAC
001
Tumor
Nerve
NI
Tumor
No NI
Nerve
No NI
D
EGFR expression in nerves in human PDAC
Gene expression level (FPKM)
25
20
15
10
5
0
ns
NI
No NI
E
EGFR expression in sorted murine DRG
2^-(DDCt) relative to WT
1.3
1.2
1.1
1.0
0.9
0.8
0.7
0.6
0.5
0.4
0.3
0.2
0.1
0.0
ns
ns
KPC
TPAC
WT
F
Human PDAC
PGP 9.5/EGFR
G
TPAC DRG
TPAC DRG
TPAC DRG
PGP 9.5/EGFR
100 µm
50 µm

Figure S10

A

Clinical parameters	low p-Paxillin	high p-Paxillin
**Tumour size**		
T1 (< 2cm)	6%	11%
T2 (2.1-4cm)	3%	6%
T3 (4.1-6cm)	78%	56%
T4 (> 6cm)	13%	28%
**Tumour grading**		
G1(well differentiated)	9%	0%
G2 (moderately differentiated)	63%	76%
G3 (not differentiated)	25%	24%
**NCTx**		
0	63%	22%
1	38%	78%
**Resection status**		
R0 (margin-free)	34%	50%
R1 (margin-positive)	66%	50%

B

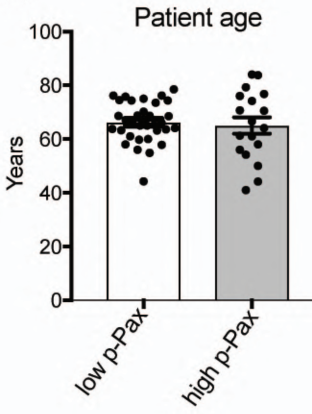

C

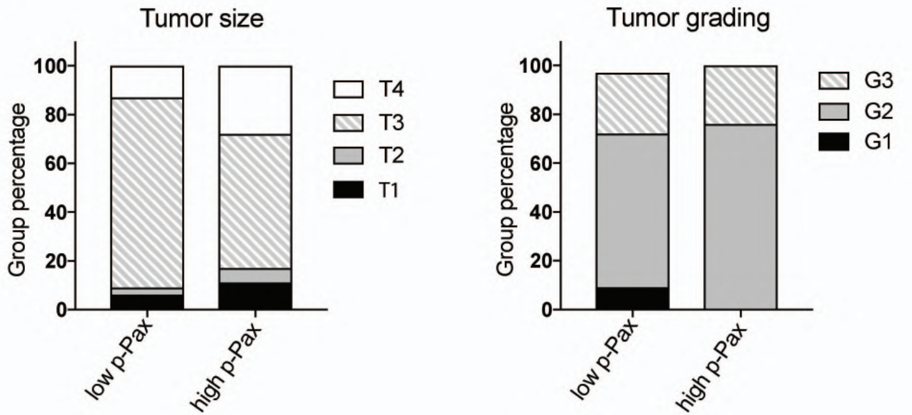

Figure S11

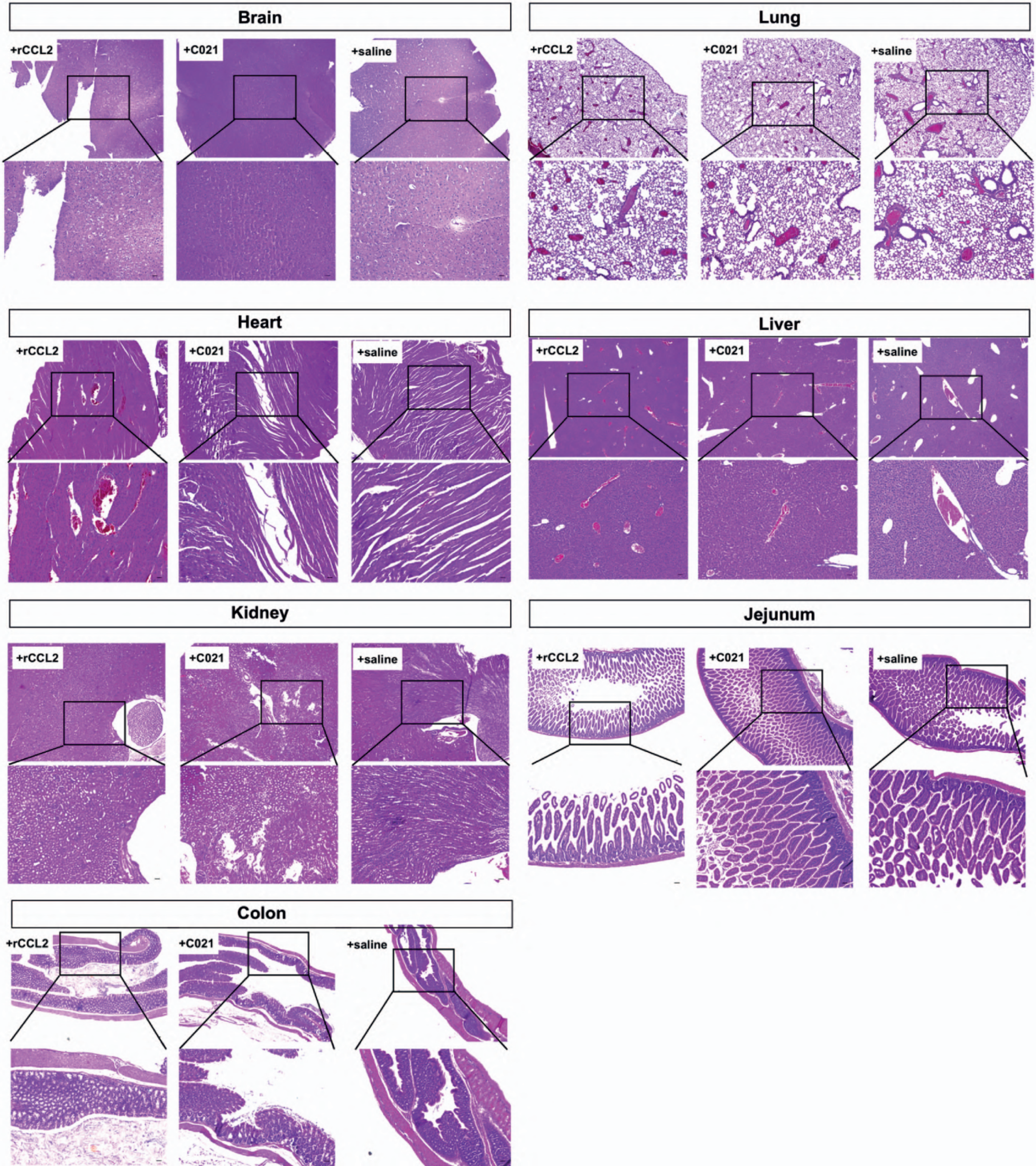
Brain
+rCCL2
+C021
+saline
Lung
+rCCL2
+C021
+saline
Heart
+rCCL2
+C021
+saline
Liver
+rCCL2
+C021
+saline
Kidney
+rCCL2
+C021
+saline
Jejunum
+rCCL2
+C021
+saline
Colon
+rCCL2
+C021
+saline

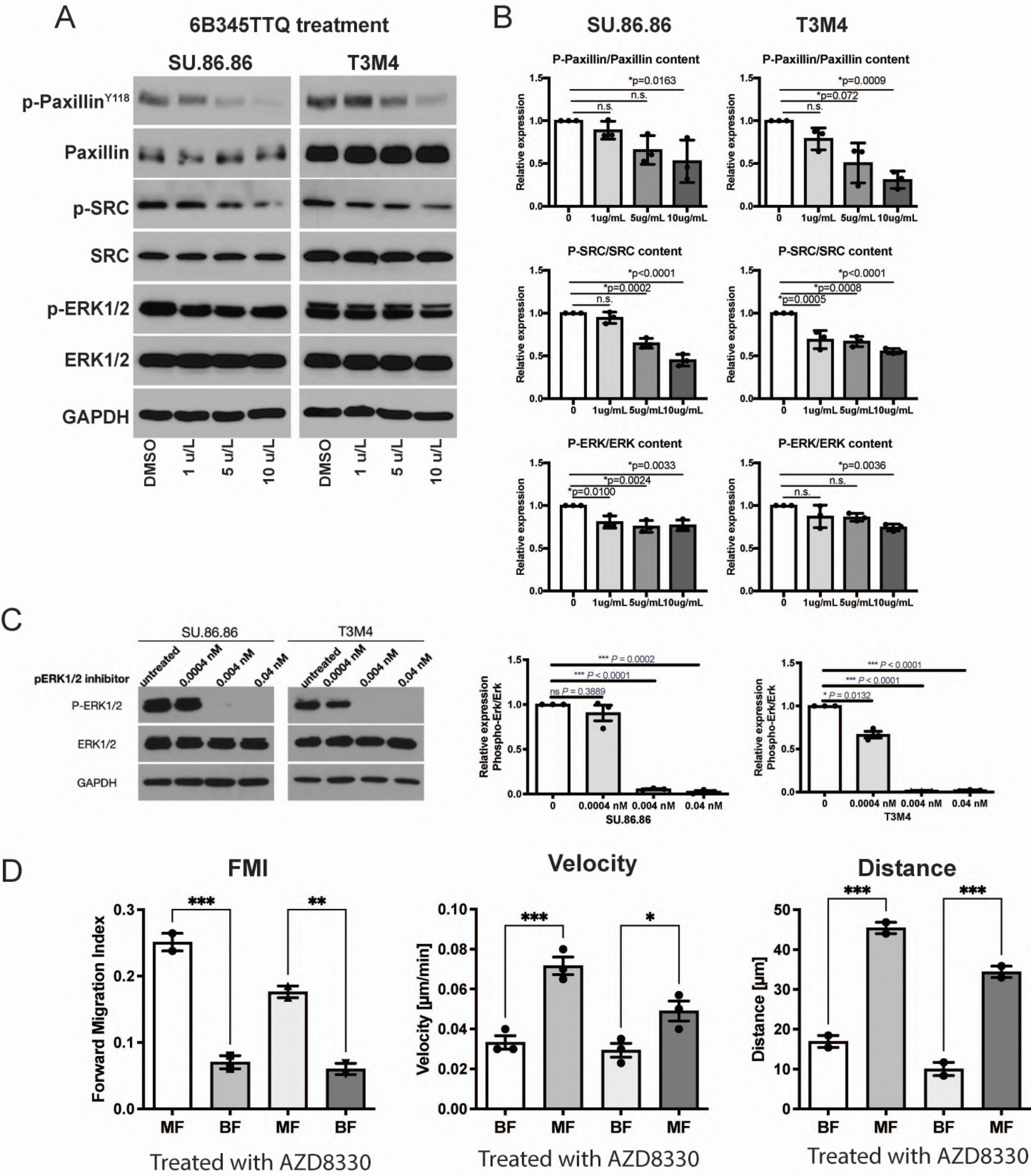
A
6B345TTQ treatment
SU.86.86
T3M4
p-Paxillin Y118
Paxillin
p-SRC
SRC
p-ERK1/2
ERK1/2
GAPDH
DMSO
1 u/L
5 u/L
10 u/L
B
SU.86.86
T3M4
P-Paxillin/Paxillin content
P-SRC/SRC content
P-ERK/ERK content
Relative expression
0
1ug/mL
5ug/mL
10ug/mL
*p=0.0163
*p=0.0009
*p=0.072
n.s.
*p<0.0001
*p=0.0002
*p=0.0008
*p=0.0005
*p=0.0033
*p=0.0024
*p=0.0100
*p=0.0036
C
SU.86.86
T3M4
pERK1/2 inhibitor
untreated
0.0004 nM
0.004 nM
0.04 nM
P-ERK1/2
ERK1/2
GAPDH
Relative expression Phospho-Erk/Erk
*** P = 0.0002
*** P < 0.0001
ns P = 0.3889
* P = 0.0132
D
FMI
Velocity
Distance
Forward Migration Index
Velocity [µm/min]
Distance [µm]
MF
BF
Treated with AZD8330

Figure S13

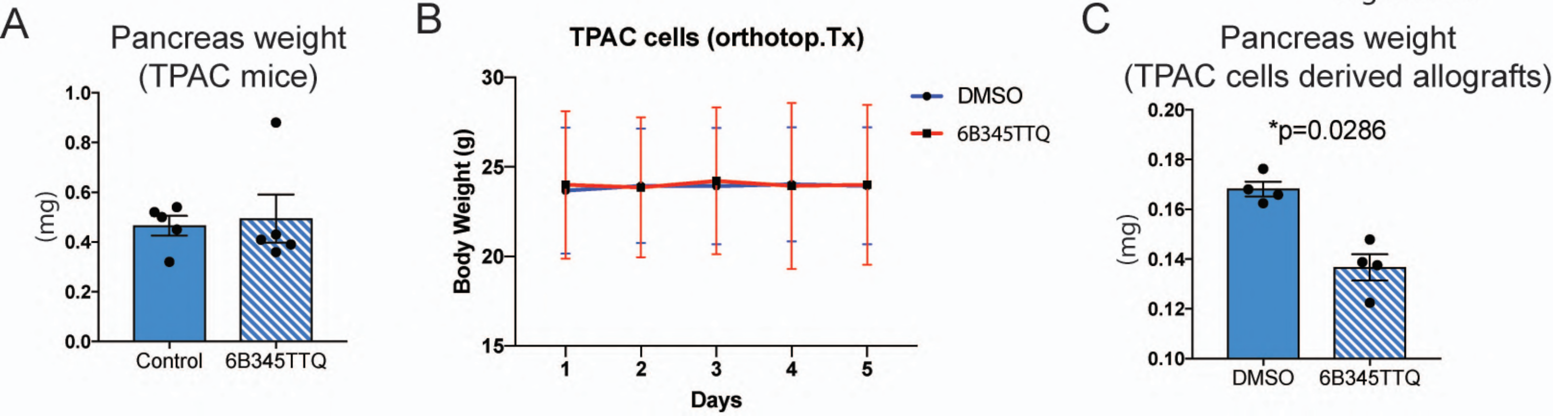
A
Pancreas weight
(TPAC mice)
(mg)
1.0
0.8
0.6
0.4
0.2
0.0
Control
6B345TTQ
B
TPAC cells (orthotop.Tx)
Body Weight (g)
30
25
20
15
1
2
3
4
5
Days
DMSO
6B345TTQ
C
Pancreas weight
(TPAC cells derived allografts)
*p=0.0286
(mg)
0.20
0.18
0.16
0.14
0.12
0.10
DMSO
6B345TTQ

Figure S14

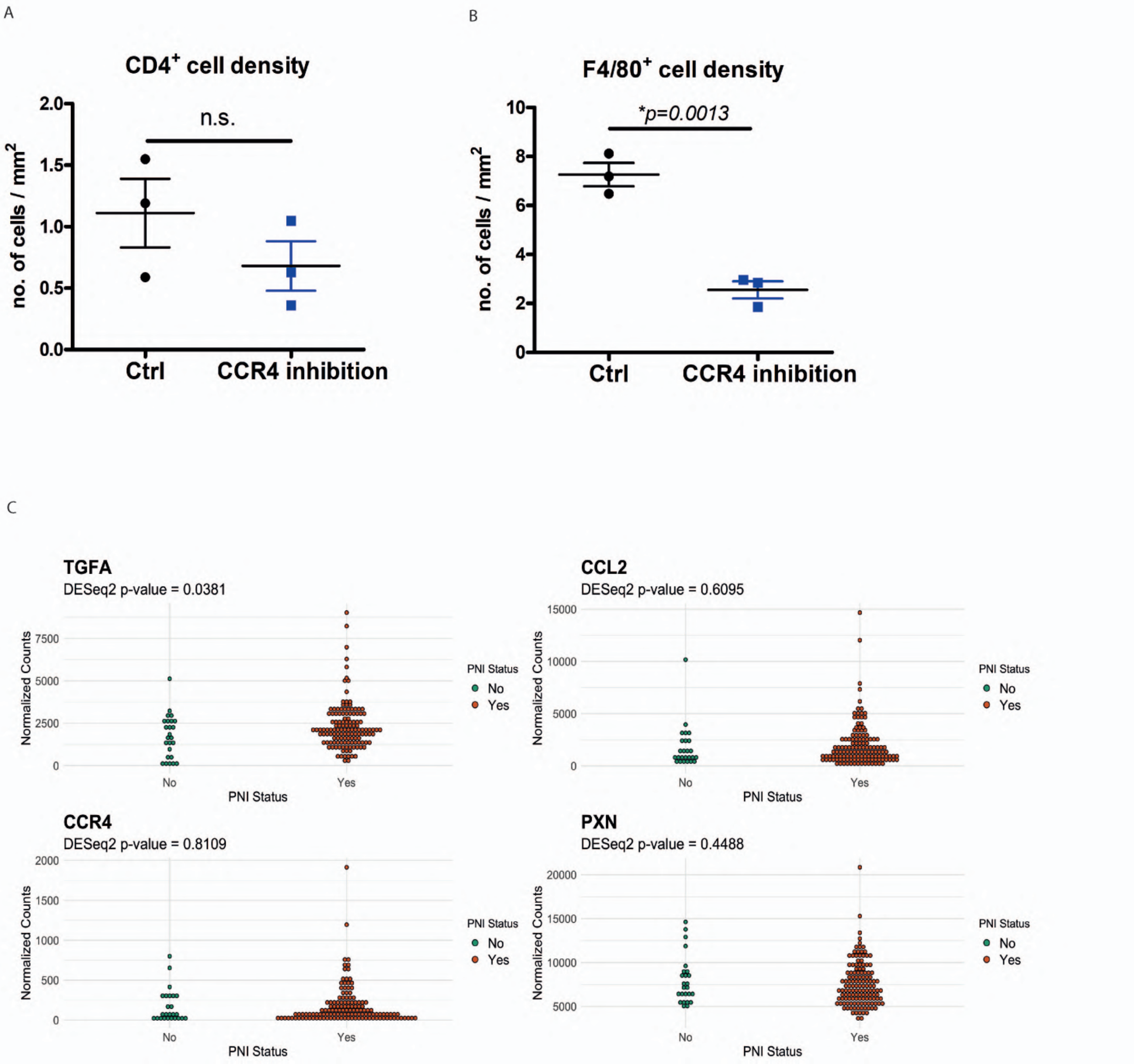
A
CD4+ cell density
no. of cells / mm2
n.s.
Ctrl
CCR4 inhibition
B
F4/80+ cell density
no. of cells / mm2
*p=0.0013
Ctrl
CCR4 inhibition
C
TGFA
DESeq2 p-value = 0.0381
CCL2
DESeq2 p-value = 0.6095
CCR4
DESeq2 p-value = 0.8109
PXN
DESeq2 p-value = 0.4488
Normalized Counts
PNI Status
No
Yes

Supplementary Table 1

GENOTYPE of the MICE

p48-Cre +/-, LSL-Kras +/-, Mek1 +/-
p48-Cre +/-, LSL-Kras +/-, LSL-Snail +/-, Ink4a +/+
p48-Cre +/-, LSL-Kras +/-, LSL-Trp53 +/+, LSL-R26-Tva +/-
p48-Cre +/-, LSL-Kras +/-, LSL-R26-Tva +/-
p48-Cre +/-, LSL-Kras +/-, p53-lox +/-, LSL-R26-Tva +/-
p48-Cre +/-, LSL-Kras +/-
p48-Cre +/-, LSL-Kras +/-, p16-lox +/+, LSL-R26-Tva +/+
p48-Cre +/-, LSL-Kras +/-, Erk1 +/+
Low-Cre +/-, LSL-Kras +/-, LSL-Snail +/-, Ink4a +/-
Pdx1-Cre +/-, LSL-Kras +/-, LSL-Snail +/-, R26-tdTo +/-
p48-Cre +/-, LSL-Kras +/-, Ink4a +/-
p48-Cre +/-, LSL-Kras +/-, LSL-Trp53 +/-, LSL-R26-Tva +/+
p48-Cre +/-, Pdx1-Cre, LSL-pAe5 +/+, LSL-Trp53 +/-
p48-Cre +/-, Pdx1-Cre +/-, LSL-pAe5 +/+, LSL-Trp53 +/-
p48-Cre +/-, LSL-Kras +/-, LSL-R26-Tva +/-, LSL-PCNA-ATG +/-
Pdx1-Flp +/-, FSF-Kras +/-, Cpa-Cre +/-
p48-Cre +/-, LSL-Kras +/-, LSL-Trp53 +/-
Pdx1-Cre +/-, LSL-pAe5 +/-
8153.46-+/-, FSF-Kras +/-, LSL-Trp53 +/-, Pdk +/-, LSL-R26-Tva +/-
p48-Cre +/-, LSL-Kras +/-, BIIR +/+, LSL-R26-Tva +/-
Pdx1-Flp +/-, LSL-Kras +/-, BIIR +/-, p53-frt +/-, Tva +/-
Pdx1-Flp +/-, FSF-Kras +/-, FSF-R26-CreER +/-, LSL-R26-Tva +/-
Pdx1-Cre +/-, LSL-Kras +/-, LSL-Snail +/+, E-cadh +/-
p48-Cre +/-, LSL-Kras +/-, LSL-Snail +/-, SCID +/+, IL2 +/+
Low-Cre +/-, LSL-Kras +/-, LSL-Snail +/-
p48-Cre +/-, LSL-Kras +/-, LSL-Snail +/-
p48-Cre +/-, LSL-pAe5 +/-, PTEN-flox +/-
Pdx1-Flp +/-, FSF-Kras +/-, LSL-R26-Tva +/-
Pdx1-Flp +/-, FSF-Kras +/-, R26+/-, R26dual+/-, R26-TdTo+/-, EGFP+/-
p48-Cre +/-, LSL-Kras +/-, LSL-Snail +/-, Tnc-ko +/+
p48-Cre +/-, LSL-pAe5 +/-
p48-Cre +/-, LSL-pAe5 +/-, LSL-Trp53 +/-
p48-Cre +/-, LSL-pAe5 +/+, LSL-Trp53 +/-
p48-Cre +/-, LSL-Kras +/-, LSL-Trp53 +/-, p16-lox +/+, LSL-R26-Tva +/+
Pdx1-Flp +/-, FSF-Kras +/-, p53-frt +/-
Pdx1-Flp +/-, FSF-Kras +/-, p53-frt +/-, R26-dual +/-,
Pdx1-Cre +/-, LSL-Kras +/-, LSL-Trp53 +/-, LSL-PCNA-ATG +/-
p48-Cre +/-, Pdx1-Cre +/-, LSL-Kras +/-, LSL-Trp53 +/-, LSL-R26-Tva +/-
p48-Cre +/-, LSL-Kras +/-, BIIR +/+
p48-Cre +/-, LSL-Kras +/-, BIIR +/-, LSL-R26-Tva +/-
p48-Cre +/-, LSL-pAe5 +/-, Flp-ERT2 +/-
p48-Cre +/-, LSL-pAe5 +/-, Erk1 +/+
p48-Cre +/-, LSL-pAe5 +/-, MEK1 +/-
p48-Cre +/-, LSL-Kras +/-, BIIR +/-
Pdx1-Flp +/-, FSF-Kras +/-, mGFAP2-Cre 77.6 +/-, R26-dual +/-
p48-Cre +/-, LSL-pAe5 +/-, PTEN-flox +/-, R26-Confetti +/-

Pdx1-Flp +/-, FSF-Kras +/-, FSF-R26-CreER +/+, p16-lox +/-, Smad4 +/+, p53-lox +/-
p48-Cre +/-, LSL-Kras +/-, LSL-Trp53 +/-, LSL-R26-Tva +/-
p48-Cre +/-, LSL-Kras +/-, LSL-Trp53 +/-, R26-tdTO +/-
Pdx1-Flp +/-, FSF-Kras +/-, FSF-R26-CreER +/-, SMAD4 +/+, p53-lox +/-
Pdx1-Cre +/-, LSL-Kras +/-, p53-lox +/+
Pdx1-Cre +/-, LSL-Kras +/-, p53-lox +/-
Pdx1-Cre +/-, LSL-Kras +/-, LSL-Trp53 +/-, E-cadh +/-
Pdx1-Cre +/-, LSL-Kras +/-, LSL-Trp53 +/-
Pdx1 Cre +/-, LSL-Kras +/-, LSL-Trp53 +/-, LSL-R26-Tva +/-
Pdx1-Cre +/-, LSL-Kras +/-, LSL-Trp53 +/-, Bl6-Raf +/-
Pdx1-Flp +/-, FSF-Kras +/-, LSL-p53-WT +/-
Pdx1-Cre +/-, LSL-Kras +/-, LSL-Trp53 +/-, E-cadh +/+, LSL-R26-Tva +/-
p48 Cre, p53, Kras (überprüft)
p48-Cre +/-,LSL-Kras +/-, LSL-PCNA-ATG +/-
Pdx1-Cre +/-, LSL-Kras +/-, LSL-R26-Tva +/-, LSL-PCNA-ATG +/-
p48-Cre +/-, Pdx1-Cre +/-, LSL-pAe5 +/-, LSL-R26-Tva +/-
p48-Cre +/-, LSL-Kras +/-, LSL-PCNA-IRES +/-
Pdx1-Cre +/-, LSL-Kras +/-
Pdx1-Cre +/-, LSL-Kras +/-, LSL-Trp53 +/-,
p48-Cre +/-, LSL-Kras +/-, R26-LacZ +/-
p48-Cre +/-, LSL-Kras +/-, LSL-R26-Tva +/+
p48-Cre +/-, LSL-Kras +/-, p16-lox +/+, LSL-R26-Tva +/-
p48-Cre +/-, LSL-Kras +/-, p16-lox +/-
p48-Cre +/-, LSL-Kras +/-, p16-lox +/-, LSL-R26-Tva +/+
p48-Cre +/-, LSL-Kras +/-, p16-lox +/-, LSL-R26-Tva +/-
Pdx1-Cre +/-, LSL-pAe5 +/-, E-cadh +/+, LSL-Trp53 +/-
Pdx1-Cre +/-, LSL-Kras +/-, p53-lox +/+, LSL-PCNA-ATG +/-, LSL-R26-Tva +/-
Pdx1-Flp +/-, FSF-PI3K +/-, R26-dual +/-
Pdx1-Flp +/-, FSF-Kras +/-, FSF-R26-CreER +/-, LSL-Trp53 +/-, DTA +/-, E-cadh +/-,
Pdx1-Flp +/-, FSF-Kras +/-, FSF-R26-CreER +/+, E-cadh +/-
Pdx1-Flp +/-, FSF-Kras +/-, E-cadh +/-, LSL-R26-Tva +/+
Pdx1-Flp +/-, FSF-Kras +/-, FSF-R26-CreER +/-, LSL-Trp53 +/-, E-cadh +/+
Pdx1-Flp +/-, FSF-Kras +/-, FSF-R26-CreER +/-, E-cadh +/+
Pdx1-Flp +/-, FSF-Kras +/-, FSF-R26-CreER +/+, LSL-Trp53 +/-, E-cadh +/-
Pdx1-Flp +/-, FSF-Kras +/-, S-Cre +/-, Ecadh +/-, LSL-R26-Tva +/+
Pdx1-Flp +/-, FSF-Kras +/-, FSF-R26-CreER +/+, E-cadh +/+
Pdx1-Flp +/-, FSF-Kras +/-, FSF-R26-CreER +/-, LSL-Trp53 +/+, E-cadh +/-, LSL-R26-Tva +/-
Pdx1-Flp +/-, FSF-Kras +/-, S-Cre +/-, LSL-R26-Tva +/-
Pdx1-Flp +/-, FSF-Kras +/-, LSL-Trp53 +/-, E-cadh +/+, LSL-R26-Tva +/+
p48-Cre +/-, LSL-Kras +/-
p48-Cre +/-, LSL-Kras +/-, LSL-R26-Tva +/-
Pdx-Flp +/-, FSF-Kras +/-, FSF-R26-CreER +/-, E-cadh +/+, Tva +/-
p48-Cre +/-, LSL-Kras +/-, LSL-R26-Tgfb-1 +/-
Pdx1-Flp +/-, FSF-Kras +/-, E-cadh +/+, R26-tdEG +/+
p48-Cre +/-, LSL-Kras +/-, Pdk +/-, Pdk-K465E +/-
p48-Cre +/-, LSL-Kras +/-, LSL-Snail +/-, LSL-R26-Tva +/-
p48-Cre +/-, LSL-Kras +/-, LSL-Trp53 +/-, LSL-Snail +/-
p48-Cre +/-, LSL-Kras +/-, LSL-Snail +/-, Ink4a +/-
p48-Cre +/-, LSL-Kras +/-, LSL-Trp53 +/-, LSL-Snail +/-, LSL-R26-Tva +/-

Pdx1-Cre +/-, LSL-Kras +/-, LSL-Snail +/-, E-cadh +/-
p48-Cre +/-, LSL-Kras +/-, Snail-KO lox +/+
p48-Cre +/-, LSL-Kras +/-, LSL-Snail +/-, p16-lox +/-
Pdx1-Flp +/-, FSF-Kras +/-, R26-tdEG +/-, LSL-R26-Tva +/-
p48-Cre +/-, LSL-Kras +/-, LSL-Snail +/-, p16-lox +/+
p48-Cre +/-, LSL-Kras +/-, p16-lox +/+
Pdx1-Flp +/-, FSF-Kras +/-, FSF-R26-CreER +/-, E-cadh +/+, LSL-Trp53 +/-, R26-tdEG +/-
Pdx1-Flp +/-, FSF-Kras +/-, E-cadh +/+, LSL-Trp53 +/-, R26-tdEG +/-
Pdx1-Flp +/-, FSF-Kras +/-, S-Cre +/-, R26-tdEG +/-, LSL-R26-Tva +/-
p48-Cre +/-, LSL-Kras +/-, LSL-Trp53 +/-, R26-tdEG +/-, LSL-R26-Tva +/-
p48-Cre +/-, LSL-Kras +/-, Pdk +/-, Pdk-L155E +/-
Pdx1-Flp +/-, FSF-Kras +/-, C-Cre +/-, LSL-Trp53 +/-, Pdk +/-, LSL-R26-Tva +/-
Pdx1-Flp +/-, FSF-Kras +/- FSF-R26-CreER +/-, LSL-p53-WT +/-, Pdk +/-, R26-tdEG +/-
Pdx1-Flp +/-, FSF-Kras +/-, FSF-R26-CreER +/-, LSL-p53-WT +/+, R26-tdEG +/-
Pdx1-Flp +/-, FSF-Kras +/-, p53-frt +/-, R26-tdEG +/-
Pdx-Flp +/-, FSF-Kras +/-, C-Cre +/-, p53-frt +/-, R26-tdEG +/-
Pdx1-Flp +/-, FSF-Kras +/- C-Cre +/-, p53-frt +/-, R26-tdEG +/-
Pdx1-Flp +/-, FSF-Kras +/-, p53-frt +/-, R26-tdEG +/
Pdx1-Flp +/-, FSF-Kras +/-, Fsp1-Cre +/-, LSL-Trp53 +/-, R26-tdEG +/-
p48-Cre +/-, Pdx1-Cre +/-, LSL-Kras +/-, LSL-Snail +/-
p48-Cre +/-, LSL-Kras +/-, Pdk +/-, R26-LSL-PB +/-, ATP
Pdx-Flp +/-, FSF-Kras +/-, p53-frt +/-
Pdx-Flp +/-, FSF-Kras +/-, C-Cre +/-, p53-frt +/-
p48-cre+/-, LSL-Kras, p53-lox +/+, LSL-R26-Tva +/-
Pdx1-Flp +/-, FSF-Kras +/-, Fsp1-Cre +/-, p53-frt +/-, R26-tdEG +/-
Pdx1-Cre +/-, LSL-Kras +/-, LSL-R26-Tva +/-
Pdx1-Cre +/-, LSL-Kras +/-, LSL-Trp53 +/-, LSL-R26-Tva +/-
Pdx1 Cre +/-, LSL-Kras +/-, LSL-Trp53 +/-, LSL-R26-Tva +/-, LSL-PCNA-ATG +/-
Pdx1-Cre +/-, LSL-Kras +/-, LSL-Trp53 +/-, E-cadh +/+
PTC +/-, LSL-Kras +/-, LSL-Trp53 +/-, Rbp +/-
p48-Cre +/-,LSL-Kras +/-, p53-lox +/-, LSL-R26-Tva +/-
Pdx1-Cre +/-, LSL-Kras +/-, LSL-Trp53 +/-, Raf +/-
p48-Cre +/-, Pdx1-Cre +/-, LSL-pAe5 +/-, LSL-Trp53 +/-, R26 +/-, LSL-R26-Tva +/-
p48-Cre +/-, LSL-Kras +/-, LSL-Trp53 +/+, Pdk +/-, Raf +/-
p48-Cre +/-, LSL-Kras +/-, PCNA-IRES-5072.2 +/-
Pdx1-Cre +/-, LSL-Kras+/-, LSL-Trp53 +/-
Pdx1-Cre +/-, LSL-Kras+/-, LSL-Trp53 +/-, LSL-R26-Tva +/-
Pdx1-Cre +/-, LSL-Kras +/-, LSL-Trp53 +/-, LSL-R26-Tva +/-, LSL-PCNA-ATG +/-
Pdx1-Cre +/-, LSL-Kras +/-, p53-lox +/-, LSL-R26-Tva +/-, Swiss-nu +/-
p48-Cre +/-, LSL-Kras +/-, LSL-Trp53 +/-, LSL-R26-Tva +/-
p48-Cre +/-, LSL-Kras +/-, LSL-Trp53 +/+, LSL-R26-Tva +/-, R26 +/-
Pdx1-Cre +/-, LSL-pAe5 +/-, p53-lox +/-
Pdx1-Cre +/-, LSL-pAe5 +/-, p53-lox +/+
Pdx1-Cre +/-, LSL-Kras +/-, LSL-Trp53 +/-, LSL-PCNA-ATG +/-, LSL-R26-Tva +/-
Pdx1-Cre +/-, LSL-Kras +/-, p53-lox +/-, LSL-PCNA-ATG +/-,
Pdx1-Flp +/-, FSF-Kras +/-, hGFAP-Cre +/-, p53-frt +/-, R26-AP +/-
Pdx1-Flp +/-, FSF-Kras +/-, FSF-R26-CreER +/-, LSL-p53-WT +/-, PTEN +/-
Pdx1-Flp +/-, FSF-Kras +/-, LSL-p53-WT +/-, R26-tdEG +/+
Pdx1-Flp +/-, FSF-Kras +/-, FSF-R26-CreER +/-, LSL-p53-WT +/-

Pdx1-Flp +/-, FSF-Kras +/-, LSL-Trp53 +/-, LSL-R26-Tva +/-
Pdx1-Flp +/-, FSF-Kras +/-, SER +/-, p53-frt +/-, R26-AP +/-
Pdx1-Flp +/-, FSF-Kras +/-, mGFAP2-Cre +/-, R26-tdEG +/-
Pdx1-Flp +/-, FSF-Kras +/-, SER +/-, p53-frt +/+, R26-tdEG +/-
Pdx1-Flp +/-, FSF-Kras +/-, FSF-R26-CreER +/+, LSL-p53-WT +/-
Pdx1-Flp +/-, FSF-Kras +/-, SER +/-, p53-frt +/+, R26-AP +/-
Pdx1-Flp +/-, FSF-Kras +/-, LSL-Trp53 +/+, Pdk +/-, LSL-R26-Tva +/-
Pdx1-Flp +/-, FSF-Kras +/-, mGFAP2-Cre +/-, p53-frt +/-, R26-tdEG +/-
Pdx1-Flp +/-, FSF-Kras +/-, SER +/-, p53-frt +/+
p48-Cre +/-, LSL-Kras +/-, BIIR +/-, LSL-R26-Tva +/-
Pdx-Flp +/-, FSF-Kras +/-, p53-frt +/-, R26-tdEG +/+
Pdx-Flp +/-, FSF-Kras +/-, p53-frt +/+, R26-tdEG +/+
Pdx1-Flp +/-, FSF-Kras +/-, FSF-R26-CreER +/-, LSL-p53-WT +/-, R26-tdEG +/-
p48-Cre +/-, LSL-Kras +/-, BIIR +/+, LSL-Trp53 +/-, LSL-R26-Tva +/-
Pdx1-Flp +/-, FSF-Kras +/-, Cpa-Cre +/-, p53-frt +/-, R26-tdEG +/+
Pdx1-Flp +/-, FSF-Kras +/-, LSL-p53 +/-, R26-tdEG +/-
Pdx1-Flp +/-, FSF-Kras +/-, GFAP-Cre +/-, LSL-Trp53 +/-, R26-tdEG +/+
Pdx1-Flp +/-, FSF-Kras +/-, Fsp1-Cre +/-, LSL-Trp53 +/-, R26-tdEG +/-, LSL-R26-Tva +/-
Pdx-Flp +/-, FSF-Kras +/-, Mek1 +/+, Mek2 +/-, R26-tdEG +/-
Pdx1-Flp +/-, FSF-Kras +/-, Fsp1-Cre +/-, FGFR +/-, LSL-Trp53 +/-, R26-tdEG +/-
Pdx1-Flp +/-, FSF-Kras +/-, Fsp1-Cre +/-, FGFR +/-, R26-tdEG +/-
p48-Cre +/-, LSL-Kras +/-, BIIR +/+, LSL-Trp53 +/-
Pdx1-Flp +/-, FSF-Kras +/-, FGFR +/+, R26-tdEG +/+
Pdx1-Flp +/-, FSF-Kras +/-, FGFR +/+, p53-frt +/+, R26-tdEG +/+
Pdx1-Flp +/-, FSF-Kras +/-, Fsp1-Cre +/-, FGFR +/+, p53-frt +/+, R26-tdEG +/+
Pdx1-Cre +/-, LSL-pAe5 +/-, LSL-Trp53 +/-, R26-tdEG +/-
Pdx1-Flp +/-, FSF-Kras +/-, E-cadh +/+, p53-frt +/-, R26-dual +/-
Pdx1-Flp +/-, FSF-Kras +/-, p53-frt +/-, Ai65-tdTom +/+
p48-Cre +/-, LSL-Kras +/-, p53-lox +/-
p48-Cre +/-, LSL-Kras +/-, p53-lox +/-, R26-tdTo +/-
Pdx1-Flp +/-, FSF-Kras +/-, FSF-R26-CreER +/+, MEK1 +/+, MAP2K2 +/+
Pdx-Flp +/-, FSF-Kras +/-, FSF-R26-CreER +/-, LSL-R26-TGFb-1 +/-
Pdx1-Flp +/-, FSF-Kras +/-, Cpa-Cre +/-, p53-frt +/+
p48-Cre +/-, LSL-Kras +/-, LSL-Snail +/-, Ink4a +/+, LSL-R26-Tva +/-
Pdx1-Flp +/-, FSF-Kras +/-, Cpa-Cre +/-, p53-frt +/-, R26-tdEG +/-
p48-Cre +/-, LSL-Kras +/-, LSL-Snail +/+
p48-Cre +/-, Ela-Cre +/-, LSL-Kras +/-, Snail-KO lox +/+
Low-Cre +/-, LSL-Kras +/-, LSL-Snail +/+
p48-Cre +/-, LSL-Kras +/-, Tnc-ko +/+
p48-Cre +/-, LSL-Kras +/-, LSL-Snail +/-, Hnf4a-lox +/-
Pdx1-Cre +/-, LSL-Kras +/-, LSL-Snail +/-, Ink4a +/-
Pdx1-Flp +/-, FSF-Kras +/-, FSF-R26-CreER +/-, LSL-Trp53 +/-, p53-lox +/-, R26-tdEG+/-
p48-Cre +/-, LSL-Kras +/-, Hnf4a-lox +/+
Pdx1-Flp +/-, FSF-Kras +/-, FSF-R26-CreER +/+, LSL-Trp53 +/-, p53-lox +/-,
Pdx1-Flp +/-, FSF-Kras +/-, R26-FSF-CreERT2 +/-, Snail-KO lox +/-
Pdx1-Flp +/-, FSF-Kras +/-, p53-frt +/-, Fsp1-Cre +/-, R26-tdEG +/+
Pdx1-Flp +/-, FSF-Kras +/-, p53-frt +/-, Myh11-CreER, R26-tdEG +/+
p48-Cre +/-, LSL-Kras +/-, LSL-Snail +/+, p16 lox +/-
Pdx1-Flp +/-, FSF-Kras +/-, p53-frt +/-, R26-tdEG +/+

Pdx1-Flp +/-, FSF-Kras +/-, p53-frt +/-, Nest-CreER, R26-tdEG +/+
Pdx1-Flp +/-, FSF-Kras +/-, p53-frt +/-, Nest-CreER, LSL-Snail +/-, R26-tdEG +/-
Pdx1-Flp +/-, FSF-Kras +/-, p53-frt +/-, Nest-CreER, R26-tdEG +/-
Pdx-Flp +/-, FSF-Kras +/-, p53-frt +/-, Nest-CreER, R26-tdEG +/+
Pdx1-Flp +/-, FSF-Kras +/-, FSF-PCNA-CreER +/-, LSL-p53 +/-, R26-AP +/-
Pdx1-Flp +/-, FSF-Kras +/-, FSF-R26-CreER +/-, LSL-Trp53 +/-, DTA+/-
Pdx1-Flp +/-, FSF-Kras +/-, FSF-R26-CreER +/-, LSL-Trp53 +/-, DTA +/-
Pdx1-Flp +/-, FSF-Kras +/-, FSF-R26-CreER +/-, LSL-Trp53 +/-, DTA +/-, E-cadh +/-, dsRED-eGFP +/-
Pdx1-Flp +/-, FSF-Kras +/-, FSF-R26-CreER +/-, LSL-Trp53 +/-, DTA +/-, E-cadh +/+?
Pdx1-Flp +/-, FSF-Kras +/-, FSF-R26-CreER +/-, Pdk +/-
Pdx1-Flp +/-, FSF-Kras +/-, LSL-Trp53 +/-, Pdk +/-, Raf +/+
Pdx1-Flp +/-, FSF-Kras +/-, FSF-R26-CreER +/-, LSL-Trp53 +/-, DTA +/-, dsRed-eGFP +/-
Pdx-Flp +/-, FSF-Kras +/-, FSF-R26-CreER +/-, LSL-Trp53 +/-
Pdx1-Flp +/-, FSF-Kras +/-, R26-tdEG +/-, R26-AP +/-
Pdx1-Flp +/-, FSF-Kras +/-, Lgr5-Cre +/-, R26-tdEG +/-
Pdx1-Flp +/-, FSF-Kras +/-, DTA +/-, R26-tdEG +/-
Pdx1-Flp +/-, FSF-Kras +/-, Lgr5-Cre +/-, LSL-p53-WT +/-, R26-tdEG +/-
Pdx1-Flp +/-, FSF-Kras +/-, FSF-R26-CreER +/-, LSL-Trp53 +/-, R26-tdEG +/-
Pdx1-Cre +/-, LSL-Kras +/-, LSL-Trp53 +/-, Pdk +/+, Raf +/+
Pdx1-Flp +/-, FSF-Kras +/-, LSL-p53-WT+/-, R26-tdEG +/-
Pdx1-Flp +/-, FSF-Kras +/-, FSF-PCNA-CreER +/-, LSL-Trp53 +/-, Pdk +/-, DTA +/-
Pdx1-Flp +/-, FSF-Kras +/-, Col-CreER +/-, PCNA-flox +/-, R26-tdEG +/-
Pdx1-Flp +/-, FSF-Kras +/-, p53-frt +/-, PCNA-flox +/-
Pdx1-Flp +/-, FSF-Kras +/-, p53-frt +/-, R26-td-EG +/-, R26-AP +/-
Pdx1-Flp +/-, FSF-Kras +/-, p53-frt +/-, PCNA-flox +/-, R26-tdEG +/-
Pdx1-Flp +/-, FSF-Kras +/-, FSF-R26-CreER +/-, p53-frt +/-, DTA +/-
p48-Cre +/-, LSL-Kras +/-, R26-LSL-PB +/-, ATP
Pdx1-Flp +/-, FSF-Kras +/-, LSL-Trp53 +/-, LSL-R26-Tva +/- , R26 +/-
Pdx1-Flp +/-, FSF-Kras +/-, FSF-R26-CreER +/+, p16-lox +/+, Smad4 +/-, p53-lox +/+
Pdx1-Flp +/-, FSF-Kras +/-, FSF-R26-CreER +/+, p16-lox +/+, SMAD4 +/+, p53-lox +/-
Pdx1-Cre +/-, LSL-pAe5 +/-, Pdk +/-, LSL-R26-Tva +/-
p48-Cre +/-, LSL-Kras +/-, BIIR +/-, LSL-R26-Tva +/+
Pdx1-Cre +/-, LSL-Kras +/-, p53-lox +/+, LSL-R26-Tva +/-, R26-tdTO +/-
Pdx1- Flp +/-, FSF-Kras +/-, R26-td-EG +/-
Pdx1-Cre +/-, LSL-Kras +/-, LSL-Trp53 +/+, E-cadh +/+
Pdx1-Cre +/-, LSL-Kras +/-, LSL-Trp53 +/-, Raf +/+
p48-Cre +/-, LSL-PCNA-IRES +/-, LSL-Kras +/-, LSL-R26-Tva-K1 +/-
p48-Cre +/-, LSL-Kras +/-, LSL-Trp53 +/-, p53-lox +/-, LSL-R26-Tva +/-
Pdx1-Cre +/-, LSL-Kras +/-, p53-lox +/-, LSL-R26-Tva +/-
p48-Cre +/-, LSL-Kras +/
p48-Cre +/-, Pdx1-Cre +/-, LSL-Kras +/-, LSL-Trp53 +/-, LSL-R26-Tva +/+
p48-Cre +/-, LSL-pAe5 +/-, LSL-R26-Tva +/-
p48-Cre +/-, LSL-Kras +/-, LSL-pAe5 +/-, LSL-Trp53 +/+
p48-Cre +/-, LSL-Kras +/-, LSL-pAe5 +/-
Pdx1-Cre +/-, LSL-pAe5 +/+
Pdx1-Cre +/-, LSL-pAe5 +/-, p53-lox +/-, LSL-Trp53 +/-
Pdx1-Flp +/-, FSF-Kras +/-, FSF-PCNA-CreER +/-, R26-td-EG +/-
Pdx1-Cre +/-, LSL-Kras +/-, LSL-PCNA-ATG +/-, Notch-1 +/-
p48-Cre +/-, LSL-Kras +/-, BIIR +/+, LSL-R26-Tva +/+

p48-Cre +/-, LSL-Kras +/-, BIIR +/+, LSL-Trp53 +/+
8153.46 +/-, FSF-Kras +/-, R26-FSF-CreERT2 +/+, R26 -/-, LSL-Trp53 +/-, Pdk +/-
8153.46 +/-, FSF-Kras +/-, DTA +/-, LSL-Trp53 +/-
Pdx1-Flp +/-, FSF-Kras +/-, LSL-Trp53 +/-
8153.46 +/-, FSF-Kras +/-, R26-FSF-CreERT2 del +/-, R26 +/+, LSL-Trp53 +/-, DTA +/-
8153.46 +/-, FSF-Kras +/-, LSL-Trp53 +/-, LSL-R26-Tva +/-
8153.46 +/-, FSF-Kras +/-, LSL-Trp53 +/-
8153.46 +/-, FSF-Kras +/-, DTA +/-, LSL-Trp53 +/+
Pdx1-Flp +/-, FSF-Kras +/-, FSF-R26-CreER +/, DTA+/-, E-cadh +/-,
8153.46 +/-, FSF-Kras +/-
p48-Cre +/-, LSL-Kras +/-, BIIR +/-, Pdk +/-
8153.46 +/-, FSF-Kras +/-, LSL-Trp53 +/+
Pdx1-Cre +/-, LSL-pAe5 +/-, LSL-Trp53 +/-, E-cad +/-
p48-Cre +/-, LSL-Kras +/-, Erk1 +/+, R26-tdEG +/-
p48-Cre +/-,LSL-pAe5 +/-, Erk1 +/+
Pdx1-Cre +/-, LSL-pAe5, R26-LSL-PB, ATP
p48-Cre +/-, LSL-pAe5 +/-, Mek1 +/+
Pdx1-Flp +/-, FSF-Kras +/-, p53 +/-,
Pdx1-Flp +/-, FSF-Kras +/-, LSL-R26-Tva +/-, R26-AP +/-
8153.46 +/-, FSF-Kras +/-, LSL-Trp53 +/+, LSL-R26-Tva +/-, Pdk +/-
p48-Cre +/-, LSL-Kras +/-, MAP2K1 +/-,
8153.46 +/-, FSF-Kras +/-, R26-FSF-CreERT2 +/-, LSL-Trp53 +/-
p48-Cre +/-, Ela-Cre +/-, LSL-Kras +/-, Snail-KO lox +/+, LSL-R26-Tva +/-
p48-Cre +/-, LSL-Kras +/-, Snail-KO lox +/+, LSL-R26-Tva +/-
Pdx1-Flp +/-, FSF-Kras +/-, Pdk +/-, BIIR +/-, p53-frt +/-, R26-tdEG +/-
Pdx1-Cre +/-, LSL-Kras +/-, LSL-Snail +/-
Pdx1-Flp +/-, FSF-Kras +/-, LyzM-Cre +/+, p53-frt +/-, Pdk +/+, BIIR +/-
Pdx1-Flp +/-, FSF-Kras +/-, Pdk +/-, Raf +/-, Braf +/- (?), p53 wt del +/-, R26-tdEG +/-
Pdx1-Flp +/-, FSF-Kras +/-, FSF-R26-CreER +/+, Pdk +/-, LSL-Trp53 +/-, dsRED-eGFP
Ela-Cre +/-, LSL-Kras +/-, Pdk +/-, LSL-R26-Tva +/-
Pdx1-Cre +/-, LSL-Kras +/-, Pdk +/-, Raf +/-, Braf +/+
Pdx1-Cre, LSL-pAe5 +/-, R26 +/-, LSL-R26-Tva +/-
Pdx1-Flp +/-, FSF-Kras +/-, FSF-R26-CreER +/-, Pdk +/-, R26-tdEG +/-, p53-frt +/-
Pdx1-Flp +/-, FSF-Kras +/-, FSF-R26-CreER +/-, Pdk +/-, Raf +/-, LSL-Trp53 +/+, dsRED-eGFP
p48-Cre +/-, LSL-Kras +/-, BIIR +/+, Cdh1 +/-,
p48-Cre +/-, LSL-Kras +/-, LSL-Trp53 +/-, BIIR +/-
p48-Cre +/-, LSL-Kras +/-, LSL-Trp53 +/-, BIIR +/+
Pdx1-Cre +/-, LSL-pAe5 +/-, p53-lox +/-, R26-Confetti +/-
Pdx1-Flp +/-, FSF-Kras +/-, FSF-R26-CreER +/-, Pdk +/-, LSL-Tva +/-, LSL-Trp53 +/-, dsRed-eGFP
Pdx1-Flp +/-, FSF-Kras +/-, FSF-R26-CreER +/-, Pdk +/+, p53-frt +/-
Pdx-Flp +/-, FSF-Kras +/-, FSF-R26-CreER +/-, LSL-p53 WT +/-
Pdx-Flp +/-, FSF-Kras +/-, LSL-p53 WT +/-, R26-tdEG+/-
Pdx-Flp +/-, FSF-Kras +/-, FSF-R26-CreER +/+, LSL-p53 WT +/-
Pdx-Flp +/-, FSF-Kras +/-, FSF-R26-CreER +/-, LSL-p53 WT +/-, R26-tdEG+/-
p48-Cre +/-, LSL-Kras +/-, LSL-Snail +/-, Hnf4a-lox +/+
p48-Cre +/-, LSL-Kras +/-, LSL-TGFβ +/-
p48-Cre +/-, LSL-Kras +/-, Pten +/-
p48-Cre +/-, LSL-Kras +/-, Pdk +/-, Pten +/-
Pdx1-Cre +/-, Br +/-, LSL-Trp53 +/-, Ink4a +/-

p48-cre +/-, LSL-Kras +/-, Ribo-tag +/-
p48-Cre +/-, Ela1-TGFa,p53 fl/fl
p48-Cre +/-, Ela1-TGFa,p53 fl/fl, p65 fl/fl
p48-Cre +/-, LSL-Kras +/- ,p53 fl/fl, p65 fl/fl

Table S2. Genotypic, age, number and survival characteristics of the analyzed mice.

Mouse line	Genotype	Number (n)	Age range	Age distribution	Median survival
**KC**	Ptf1a-Cre$^{ex1/+}$;LSL-Kras^{G12D}	10	250-400 days	2 mice <250 d<br>3 mice 250-400 d<br>5 mice >400d	467 days
**KPC**	Ptf1a-Cre$^{ex1/+}$;LSL-Kras^{G12D};<br>Trp53$^{fl/fl}$	10	49-61 days	2 mice 49-50 d<br>5 mice 51-54 d<br>3 mice 54-61 d	61.5 days
**TPC**	Ela-TGFalpha;Trp53$^{\Delta/\Delta}$	12	193-339 days	2 mice 150-200 d<br>3 mice 201-250 d<br>2 mice >250 d	300 days
**TPAC**	Ela-TGFalpha;Trp53$^{\Delta/\Delta}$;p65$^{\Delta/\Delta}$	15	316-689 days	3 mice 300-350 d<br>5 mice 350-400 d<br>4 mice 400-450 d<br>3 mice >450 d	360 days

sample number	sample	#reads on-target	#reads G12G-(GGT)
1	spleen TPAC 164	56.901	56.721
2	spleen TPAC 186	56.901	55.265
3	spleen TPAC 194	60.985	60.803
4	spleen TPAC 197	61.121	60.886
5	spleen KPC 324	55.335	25.412
6	spleen KPC 457	57.545	26.146
7	pancreas TPAC normal 164	57.393	57.195
8	pancreas TPAC tumour 186	56.901	56.691
9	pancreas TPAC normal 194	57.398	57.220
10	pancreas TPAC tumour 194	60.407	60.190
11	pancreas TPAC normal 197	48.745	48.554
12	pancreas TPAC tumour 197	57.533	57.326
13	pancreas KPC normal 324	60.504	26.731
14	pancreas KPC tumour 324	49.111	21.723
15	pancreas KPC normal 457	54.151	26.049
16	KPC T9801 cell line	54.492	17.857
17	TPAC 5067 cell line	64.835	64.625

#reads G12D-(GAT)	%G12G	%G12D
8	99,7%	0,0%
25	97,1%	0,0%
6	99,7%	0,0%
9	99,6%	0,0%
29.627	45,9%	53,5%
31.123	45,4%	54,1%
11	99,7%	0,0%
10	99,6%	0,0%
14	99,7%	0,0%
9	99,6%	0,0%
8	99,6%	0,0%
8	99,6%	0,0%
33.463	44,2%	55,3%
27.173	44,2%	55,3%
27.822	48,1%	51,4%
36.280	32,8%	66,6%
9	99,7%	0,0%

Antibody	Catalogue number	Application (Dilution)	Source
Rabbit anti-phospho-paxillin (Y118) Ab	2541	WB (1:1000), IHC (1:50)	Cell Signaling Technology
Rabbit anti-paxillin Ab	12065S	WB (1:1000)	Cell Signaling Technology
Rabbit anti-phospho-Src (Y416) Ab	2101S	WB (1:1000)	Cell Signaling Technology
Rabbit anti-Src Ab	2102S	WB (1:1000)	Cell Signaling Technology
Rabbit anti-phospho-FAK (Y397) Ab	8556S	WB (1:1000)	Cell Signaling Technology
Rabbit anti-FAK (D507U) Ab	71433S	WB (1:1000)	Cell Signaling Technology
Rabbit anti-phospho-ERK1/2 (T202/Y204) Ab	4370S	WB (1:2500)	Cell Signaling Technology
Rabbit anti-ERK1/2 (137F5) Ab	4695s	WB (1:2500)	Cell Signaling Technology
Rabbit anti-PGP9.5 Ab	Z5116	IF (1:500)	Dako Deutschland GmbH
Mouse anti-S100 Ab	MAB079-1	IHC (1:1000)	EMD Millipore
Mouse anti-pan-Cytokeratin Ab	Ab17154	IF (1:200)	Abcam
Rat anti-CK19 Ab	-	IF (1:50)	Developmental Studies Hybridoma Bank
Mouse anti-GAPDH Ab	SC-32233	WB (1:1000)	Santa Cruz Biotechnology
Phalloidin-TRITC	P1951	IF (0.001mg/mL)	Sigma-Aldrich Chemie GmbH
Alexa Fluor goat anti-mouse IgG 488/594	1834337/1830459	IF (1:200)	Thermo Fisher Scientific
Alexa Fluor goat anti-rabbit IgG 488/594	1885240/1851471	IF (1:200)	Thermo Fisher Scientific
Goat HRP-Labelled Polymer Anti-Mouse Ab	K4000	IHC	Dako Deutschland GmbH
Goat HRP-Labelled Polymer Anti-Rabbit Ab	K4003	IHC	Dako Deutschland GmbH
Rabbit anti-EGFR antibody	Ab52894	IF (1:50)	Abcam

Isolation of murine tumor cells

Dilution Buffer	for 1l:<br>770 ml NBSS<br>3,3 ml 1M HEPES<br>10 ml 100X MEM Amino acids<br>10 ml 100X MEM Nonessential amino acids<br>10 ml 100X Sodium pyruvate<br>135 µl 200mM $MgCl_2$<br>80 µl 100mM $CaCl_2$<br>10 ml 0,68M trisodium citrate, pH 7,6<br>2,1 g glycerol<br>H_2O
Enzyme:	Liberase Blenzyme (25 U/ml)
Trypsin Inhibitor:	Oval Trypsin Inhibitor (50 mg/ml) = OTI
Digestion Buffer:	10 ml Dilution Buffer<br>200 µl Liberase<br>50 µl OTI
Culture Medium	DMEM (440ml)<br>100X MEM Nonessential amino acids (5 ml)<br>1% PS (5ml PS)<br>10% FCS (50ml FCS)

1. Remove the pancreas quickly and place it into a Petri dish kept on ice.
2. Chop pancreas into small pieces with sterile scalpel.
3. Incubate the suspension with 5 ml Digestion Buffer at 37°C for 10 min.
4. Transfer carefully the suspension into a 50 ml Falcon tube.
5. Centrifuge tube at 1200 rpm 5min. Pour off the supernatant. Add 5 ml Digestion Buffer and incubate the suspension at 37°C for 10 min.
6. Transfer carefully the suspension into a 50 ml Falcon tube.
7. Centrifuge tube at 1200 rpm 5min. Pour off the supernatant. Resuspend the cell pellet in 10 ml Dilution Buffer + 50 µl OTI. Filter through a sieve with a 100-µm mesh. Rinse the dish with 5 ml Dilution Buffer + 25 µl OTI and collect all tissue on the sieve.
8. Centrifuge tube at 1200 rpm 5min. Pour off the supernatant. Resuspend the cell pellet in 10 ml Dilution Buffer + 50 µl OTI.
9. Centrifuge tube at 1200 rpm 5min. Pour off the supernatant. Resuspend the cell pellet in 10 ml Culture Medium.

Supplementary figure legends

Figure S1 (A) Genotype, age, number and survival characteristics of the analyzed mice. (B) Kaplan-Meier survival curves of KC and KPC mice, TPC and TPAC mice. (C) Western blot of p65 and p53 in representative TPC and TPAC pancreatic cancer cells. β-Actin served a loading control. PC = positive control. Delta (Δ) indicates the lost p65 allele. Immunoblots confirm the absence of the p65 (Δp65) and p53 protein in TPAC mice. (D) Representative images of NI severity level score 0, 1, and 2. (E) Representative images of neural invasion from KPC, TPC and TPAC mouse. (F) Graphs show the innervation density of different mouse genotypes. (G) Graphs show the velocity of primary cancer cells isolated from KC, KPC, TPC and TPAC towards neural cells in a migration assay. (H) Graphs showing the velocity and distance of Schwann cells towards different cancer cells.

Figure S2 Immunostaining of the primary tumor, liver and lung metastases of TPAC mice (aged 360 days) for detecting nerves. Anti-Amylase antibody was used to detect cancer cells in the TPAC model, and anti-PGP9.5 to detect nerves.

Figure S3 Microarray-based transcriptomic comparison of TPAC vs. TPC cancer cells. (A) Gene set enrichment analysis (GSEA) plots. (B) KEGG-based analysis of the differentially regulated pathways. (C) Heatmap of the differentially regulated targets. (D) Pie chart of the types of altered pathways.

Figure S4 (A) Representative H&E-stained sections of pancreata from TPC and TPAC mice at the indicated time points. Scale bars represent 200µm. (B) Quantification of BrdU-positive cells in TPC and TPAC pancreata. Analysis performed on 3 individual mice per group. (C) Representative H&E-stained sections of pancreatic tumor from TPC and TPAC mice. Scale bars represent 200µm. (D) Pathologist based tumor characterization of TPC and TPAC pancreatic tumors. Tumors were divided into three groups: acinar cell carcinoma, PDAC, and mixed. Metastases were quantified. (E) Ras activity assay with lysates of cancer cells isolated

from TPAC tumors, KPC tumors, and (control) human pancreatic ductal epithelial cells (HPDE). (F) TGFa expression as determined via QRT-PCR from lysates obtained from KC mice (pre-invasive), KPC, TPC and TPAC tumors, as well as TGFa protein levels as determined via ELISA (Abcam, UK) in the KPC, TPC and TPAC tumors.

Figure S5 Analysis of the leading source of TGFa using single-cell RNA-sequencing data of human pancreatic ductal adenocarcinoma. (A and F) UMAP visualization shows the distribution of cells and clusters. A: Two datasets (GSE155698, GSE156405) were downloaded from the GEO database. F: The dataset (CRA001160) for validation was downloaded from the GSA database. The datasets were analyzed using the Seurat R package. (B and G) Dot plot with annotated cell populations based on clustering on top expression gene markers. B: The datasets GSE155698 and GSE156405. G: The dataset CRA001160. (C and H) Dot plot shows the expression profile of top expression genes at the cluster level and facilitates the inter-cluster comparison. C: The datasets GSE155698 and GSE156405. H: The datasets CRA001160. (D-E and I-J) The expression profile of TGFa at the cell level(D) and cluster level(E) for the datasets GSE155698 and GSE156405. The expression profile of TGFa at the cell level (I) and cluster level (J) for the dataset CRA001160.

Figure S6 (A) The most upregulated genes in the RNA microarray analysis of murine cancer cells isolated from the TPAC, TPC and KPC genotypes. (B) Comparison of the *Npy* and *Fos* expression in the TPAC, TPC and KPC cancer cells. (C) Experimental design: cancer cells and DRGs were monocultured or co-cultured for 48 hours with subsequent measurements of chemokines in the cell media with ELISA. (D) Content of CCL2 in the media of cells. (E) Representative pictures of Western blot from SU.86.86 and T3M4 cells treated for 15 and 25 minutes with conditioned media (CM) from DRG neurons. As loading control, GAPDH was used; (D) Graphs indicating content of p-paxillin/paxillin after treatment of DRG CM. (F) Graphs showing content of p-paxillin/paxillin after treatment of CCR2 antagonist. (G-J) Western blot images of the human PDAC cancer cell lines SU86.86 and T3M4 treated with recombinant

CCL2 (rCCL2) or the CCR4 inhibitor C021 and blotted against p-Src, Src, p-ERK or ERK. GAPDH served as loading control, and the GAPDH blot for rCCL2 treatment (panel G) is the same as on Figure 3F due to membrane stripping.

Figure S7 Comparative analysis of the sites of CCL2 expression in PDAC. (A) Semi-quantitative scoring analysis of the immunostaining density for CCL2 in human PDAC specimens (n=14). (B) Representative immunophotographs. (C) Bar charts comparing the immunostaining scores of tissue substructures in human PDAC specimens (left) and in TPAC tumors (right). (D) *Ccl2* expression levels in available single-cell RNA sequencing datasets of mouse dorsal root ganglia/DRG (https://www.ncbi.nlm.nih.gov/pmc/articles/PMC7307422/). (E) Comparative expression levels of *Ccl2* in the murine DRG FACS-isolated from the KPC vs. TPAC genotype. T-test.

Figure S8 FACS sorting of murine pancreatic tumors. Representative FACS plots of sorted cells populations from pancreas of KPC (A) and TPAC (B) mice. (C) Graphs with percentages of sorted cell populations from KPC and TPAC mice; (D) Fold change of Ccl2 mRNA content in TPAC sorted cell populations relative to corresponding sorted cells from KPC mice (red line) n=2; (E) Fold change of Ccl2 mRNA content in TPAC DRGs relative to KPC DRGs (n=3).

Figure S9 Epidermal growth factor (EGF) receptor (EGFR) expression in dorsal root ganglia (DRG) neurons and nerves in PDAC. (A) Spatial transcriptome analysis using the NanoString GeoMx ® DSP platform and the whole mouse transcriptome were performed on the FFPE sections of DRG extracted from the spinal cord levels T8 to T12. NeuN was used as the morphology marker for specific detection of neurons within the DRG. (B) Levels of EGFR expression in the DRG of different mouse mutants in this study. WT: wild type. (C) Spatial transcriptome analysis at the NanoString GeoMx ® DSP platform with the human transcriptome on the FFPE sections from two PDAC patients. Regions of interest (ROIs) included invaded (NI) vs. non-invaded (no NI) nerves that were manually selected by using the

S100 as the pan-neural marker. (D) Levels of EGFR expression in invaded (NI) vs. non-invaded (no NI) nerves in human PDAC. (E) Comparison of the *Egfr* mRNA levels in the FACS-sorted DRG neurons of wildtype (WT), KPC and TPAC mice. (F) EGFR immunostaining in nerves within human PDAC sections. Red: EGFR, Green: PGP9.5 as pan-neural marker. (G) Immunostaining of murine DRG from TPAC mice against the EGFR and the pan-neural marker PGP9.5.

Figure S10 (A, C) Pathology characteristics of tumors in PDAC patients (n=57). (B) Patient age of high p-paxillin (p-pax) group and low p-paxillin group. NCTx: neoadjuvant chemotherapy.

Figure S11 Representative images of brain, lung, heart, liver, kidney, jejunum, and colon from the recombinant CCL2 (rCCL2) treatment group, CCR4 antagonist (C021) group and control (saline) group.

Figure S12 (A) Western blots of human pancreatic cancer cell lines SU86.86 and T3M4 treated with the p-paxillin inhibitor 6B345TTQ at different concentration. (B) Relative content of p-paxillin/paxillin, p-Src/Src and p-Erk/Erk in treated cells. (C) Western blots of cancer cell lines treated for 1 hour with the MEK inhibitor AZD8330 and content of phosphorylated Erk1/2. (D) Forward migration index, velocity and distance of cancer cells in the 3D migration assay from SU86-86 cancer cells pre-treated with AZD8330.

Figure S13 (A) Pancreas weight in 6-B345TTQ-treated TPAC mice and control (DMSO)-treated TPAC mice. (B-C) Body weight and pancreas weight in recipient mice transplanted with TPAC cancer cells.

Figure S14 (A) Density of $CD4^+$ cells in the tumors of TPAC mice treated with the CCR4 inhibitor C021 vs. control (Ctrl). n.s. : not significant. N= 3 per group, unpaired t-test. (B) Density

of F4/80^{+} cells in the tumors of TPAC mice treated with the CCR4 inhibitor C021 vs. control (Ctrl). N= 3 per group, unpaired t-test. (C) Differential expression analysis (DESeq2) of TGFa, CCL2, CCR4 and paxillin (PXN) in the tumor mRNA of pancreatic cancer patients within the TCGA as compared with regard to the perineural invasion (PNI) status [yes (n=134) vs. no (n=25)] of the patients.

Supplementary table legends

Table S1. Full list of the screened genotypes.

Table S2. Genotypic, age, number and survival characteristics of the analyzed mice.

Table S3. Exon sequencing results for the Exon 2 of the *Kras* locus in TPAC and KPC mouse tumor, normal tissue, and cancer cell samples.

Table S4. (A) Primary and secondary antibodies. **(B)** ELISA kits.

Table S5. Protocol for the isolation of primary murine cancer cells from the PDAC mouse models.

Supplementary methods

Cell lines, cell culture and treatment

All human cancer cell lines were purchased from American Type Culture Collection (ATCC), and the pancreatic cancer cell line T3M4 was a kind gift from Dr. Metzgar (Department of Microbiology and Immunology and the Department of Biochemistry, Duke University Medical Center, Durham. North Carolina). All cancer cell lines were cultured according to the supplier's recommendations at 37°C, 5% CO_2. The human immortalized pancreatic epithelial cell line HPDE was a kind gift from Professor M.S. Tsao of the Ontario Cancer Institute (Toronto, Canada) and was cultured with Keratinocyte SFM, +EGF + bovine pituitary extract supplemented with 1 x antibiotic-antimycotic (1, 2). Murine pancreatic cancer cells were isolated via mechanical dissociation of the pancreatic cancer tissue with scalpel and enzymatic digestion with trypsin, followed by serial passaging for selection of the proliferating cancer cell clones. For a detailed protocol, please refer to Table S3. Two different primary cell types per genotype were used in the study.

Migration assay with time-lapse imaging microscopy

3D migration assay with time-lapse microscopy was used to quantify the migration of cancer cells, as described previously (3-6). In brief, $1x10^5$ of SU86.86 cells and DRG neurons isolated from 3-5 days old C57BL/6N mice were separately suspended in an extracellular matrix (ECM) (Sigma-Aldrich, Taufkirchen, Germany) and placed as a gel droplet with a distance of 1mm. Both droplets were connected with fibronectin bridges in order to create a gradient of secreted molecules. Additionally, on the opposite side, an empty ECM gel (negative control) was pipetted and connected with a fibronectin bridge. After polymerization of droplets and bridges in the incubator, neurobasal medium, supplemented with 100U/mL penicillin and 100 µg/mL streptomycin, 2% B-27, 0.5mM L-Glutamine and 10% FCS was applied to the assay. Cells were cultured in the chamber with 37°C and 5%CO_2 for 48 h. The migration was analysed by time-lapse video microscopy (Zeiss AxioObserver Z1, Germany, equipped with a CO2 incubation chamber, an AxioCam camera and a plan-neoluar 10x/0.3 PH1 M27 objective.). To quantify the migratory behaviour, a photomicrograph was taken by the digital time lapse

microscope every 15 min for a total of 12 to 24 hours. The videos were analysed at with the ImageJ software (version 1.44p, NIH, USA). A total of 30 cells at each front were randomly selected and tracked via “manual tracking” plug-in of the ImageJ software. This plugin generates the following morphometric parameters of cell migration in the “chemotaxis and migration tool” (Ibidi, München, Germany): 1) the Euclidean distance (μm), which is net displacement, of the migrating cells, 2) the velocity of migrating cells (μm/min), and 3) the forward-migration index/FMI, which corresponds to the proportion of the Euclidean distance to the total accumulated migration distance. A higher FMI implies a more targeted (linear) migration, as previously described (3-6).

Schwann cell (SC) outgrowth assay

5mm freshly isolated sciatic nerve trunks of C57BL/6J mice were placed between ECM bridges connecting primary cancer cells isolated from KC, KPC, TPC, or TPAC in ECM gel droplets and empty ECM gel droplet (Fig. 1D). SC were identified the in this assay first based on their typical morphology (spindle-like cells) that can be easily discerned from the more flat cancer cells, and second after fixation and co-staining against GFAP/S100 stain, as shown previously (4). The SC migration was documented via time-lapse-microscopy after 24 hours for 7 days, and we quantified the extent of SC migration toward the different GEMM PDAC cells, as described previously (4).

Treatment of cancer cells with conditioned media of DRG neurons

DRG neurons isolated from the 2-14 days old new-born mice were seeded in 24-well plates coated with 4μg/mm^2 poly-D-lysine hydrobromide, supplied with 500 μL of neurobasal medium supplemented with 100U/mL penicillin and 100 μg/mL streptomycin, 2% B-27, 0.5mM L-Glutamine and 10% FCS. 24 hours later medium was changed to serum-free medium and cells were further cultured for 48 hours. Conditioned serum free media (CM) were collected and kept at -80°C. Cancer cells were seeded in 6-well-plate and cultured until 70% confluency. Prior to treatment, cancer cells were serum-starved overnight and treated after medium change with control medium or CM from DRG neurons for 15 and 25 minutes.

GeneChip microarray

Total RNA from cancer cells was isolated with the RNEasy Plus Mini Kit (Qiagen, Hilden, Germany) according to the manufacturer's instructions. Sample preparation for microarray hybridization was carried out as described in the Ambion WT Expression Kit Protocol (Life Technologies, Carlsbad, CA, USA) and the Affymetrix WT Terminal Labeling and Hybridization User Manual (Affymetrix, Inc., Santa Clara, CA, USA).
In brief, 300 ng of total RNA were used to generate double-stranded cDNA. 12 µg of subsequently synthesized cRNA was purified and reverse transcribed into sense-strand (ss) cDNA, whereat unnatural dUTP residues were incorporated. Purified ss cDNA was fragmented using a combination of uracil DNA glycosylase (UDG) and apurinic/apyrimidinic endonuclease 1 (APE 1) followed by a terminal labeling with biotin. 3,8 µg fragmented and labeled ss cDNA were hybridized to Affymetrix Mouse Gene 1.0 ST arrays for 16 h at 45 °C in a rotating chamber. Hybridized arrays were washed and stained in an Affymetrix Fluidics Station FS450, and the fluorescent signals were measured with an Affymetrix GeneChip Scanner 3000 7G.

Sample processing was performed at the Affymetrix Service Provider and Core Facility, "KFB - Center of Excellence for Fluorescent Bioanalytics" (Regensburg, Germany; www.kfb-regensburg.de).

Microarray data processing and analyses

Summarized probe set signals were calculated by using the RMA(7) algorithm with the Affymetrix GeneChip Expression Console Software (GeneChip® Mouse gene 1.0 ST array) at the "*Kompetenzzentrum für Fluoreszente Bioanalytik*" (Regensburg, Germany). In details, after exporting into Microsoft Excel, average signal values, comparison fold changes and significance P values were calculated. Probe sets with a fold change above 2.0-fold and a student's t test P value lower than 0.05 were considered as significantly regulated.

Affymetrix identifiers were first mapped to the mouse Entrez Gene Symbols and Volcano plots of DEGs were generated using R package ggplot2 (8) . For pathway enrichment analysis, the

mouse Entrez Gene Symbols of DEGs were converted to their human homologues using HomoloGene from the National Center for Biotechnology Information (NCBI; http://www.ncbi.nlm.nih.gov/homologene?DB=homologene) and the ToppFun from the ToppGene Suite [https://toppgene.cchmc.org/] was used to search for over-represented pathways. Finally, pathway-level networks of DEGs were constructed using the STRING data base [https://string-db.org/], selecting experiments, databases, text mining and co expression as interaction sources with medium confidence (i.e. an interaction score >= 0.4).

Analysis of single cell RNA sequencing data

By screening single-cell RNA sequencing data from untreated pancreatic ductal adenocarcinoma patients, available datasets GSE155698, GSE156405 from the GEO database and CRA001160 from the GSA database were selected for downstream analysis. The Seurat package was applied to analyze the data by following default parameters unless specified setting otherwise. Low quality cells (<300 genes/cell, <3 cells/gene and >15% mitochondrial genes) were excluded. Each filtered matrix considered as a Seurat object was implemented to the merge function. The cluster-specific marker genes were identified by running the FindAllMarkers function to the normalized data, then used to assign cellular identities to clusters. After annotation for each cluster, UMAP plot was utilized to reveal the expression level of TGFa (*Tgfa*) gene at the cell level and cluster level.

For the comparison of *Ccl2* expression levels in the DRG cells, mouse DRG single-cell RNA sequencing dataset GSE139088 from the GEO database was selected for downstream analysis. The Seurat package was performed with default setting unless specified parameters otherwise. Low-quality cells (<300 genes/cell, <3 cells/gene, and >15% mitochondrial genes) were excluded. The FindAllMarkers function was conducted to identify cluster-specific markers for the annotation of cellular identities for each cluster, then visualized the expression level of the *Ccl2* gene at the cell level and cluster level.

Exon sequencing

An amplicon of Exon2 of murine Kras was amplified using Kapa Hifi Hotstart polymerase (Roche) with primers containing TruSeq overhangs. Amplicons were then were finally amplified using barcoded primers with Illumina P5 and P7 overhangs. The libraries were sequenced on a NextSeq 500 (Illumina) in a 61 cycles single end run. Raw reads were mapped to Kras reference sequence (Ensemble release GRCm38p4, Genome Reference Consortium). Variant allele frequencies on chr6 at position 145246771 were calculated. The primer sequences were as follows:

mKras-Tru-f 5'-ACACTCTTTCCCTACACGACGCTCTTCCGATCTAAGGCCTGCTGAAAATGACTGA-3'

mKras-Tru-r 5'-GTGACTGGAGTTCAGACGTGTGCTCTTCCGATCTACACCCAGTTTAAAGCCTTGGA-3'

Orthotopic transplantation in the pancreas

For orthotopic transplantations, as donor cells we used pancreatic cancer cells isolated from KPC and TPAC mice (55). In brief, 1×10^6 donor cells in 100 µl DMEM for orthotopic transplantation were injected with 26-gauge needles into the exposed pancreatic body of anesthetized mice. Mice were treated with the p-paxillin inhibitor 6-B345TTQ (B7438, Sigma) for 5 days after 3 weeks of cell implantation and sacrificed after the treatment. Mice were treated with the p-paxillin inhibitor for 5 days after 1 week of cell implantation and sacrificed after the treatment. Pancreas tissues from orthotopic transplantation mice were collected and embedded into paraffin. Occurrence of tumor or metastasis was visualized on 3 µm paraffin sections stained with haematoxylin-eosin on a brightfield microscope (Keyence, Japan) and quantified with the QuPath Software (Centre for Cancer Research & Cell Biology at Queen's University Belfast, UK).

Quantitative real-time PCR (qRT-PCR)

Extraction of mRNA from human and murine tissues and cells, cDNA synthesis and QRT-PCR for diverse growth factors were essentially performed as described previously (5). The primer

sequences were as follows: CCL2 primers: (F) 5-GCTTCAGATTTACGGGTC-3, (R) 5-CTCAGCCAGATGCAGTTA-3; NPY primers: (F) 5-TACTCCGCTCTGCGACACTA-3, (R) 5-GGGTCTTCAAGCCTTGTTCT-3. EGFR primers: (F) 5-TGGGACCAGCATTCCATTAC-3, (R) GGC TGA AAG GCA GTT AGT AGA A. Human TGFA primers: (F): 5-GAG CCC TCG GTA AGT ATG TTT AG-3; (R): 5-CAT AGT GGA GGT GAC TTG TTA GAG-3

Immunohistochemical and immunofluorescence staining, imaging

For immunostainings, paraffin sections (3 µm) and cells cultured in monolayers were stained with respective antibodies described in supplementary table 1 (Table S4). For the MCP-1/CCL2 immmunostaining, a rabbit polyclonal antibody (HPA019163, Sigma-Aldrich, Germany) was used. For immunofluorescence, secondary antibodies conjugated with Alexa® Fluor 488 or 594 were used. As counterstaining, DAPI was used. For immunohistochemistry, sections were stained with secondary antibody conjugated with horse-reddish peroxidase and visualized with DAB kit (Dako, K3468). The sections were digitalized on a Panoramic MIDI II Slide Scanner (3DHistech, Budapest, Hungary) for image viewing and analysis. The histological specimens were analysed by IED, XW and RI, who were blinded to the genotype during the analysis. We chose two sections per mouse that were at least 10 consecutive sections, i.e. at least 30 µm, away from each other for capturing separate, yet still in-tumor regions and to account for intratumoral heterogeneity. The NI severity score was adjusted for the number of nerves on each section according to the following formula (9-11): NI severity score = [(no. of nerves/n x “score 0”) + (n x “score 1/perineural invasion”) + (n x “score 2/endoneural invasion”)] / total no. of nerves.

The number of nerves on each section was determined with the help of the QuPath software (version: 0.1.2, The Queen's University of Belfast, Northern Ireland) by automatic capturing the number of PGP9.5-immunostained nerves on the digitally scanned tumor sections.

The scoring of CCL2 immunoreactivity in the human (n=14) and TPAC mouse (n=10) pancreatic cancer tissue was rated at four levels: negative=0, weak positive=1, moderate

positive=2, strong negative=3, evaluated based on the intensity of the CCL2 immunostaining and the expression level threshold. The immunostaining scoring for CCL2 in each component (acinus, tumor, immune cells, fibroblasts) was then compared with the expression scoring in nerves.

Western blot and ELISA

For the Western blots, cells were washed with 1xPBS after treatment and total protein was extracted with RIPA lysis buffer (R0278, Sigma, Taufkirchen, Germany) supplemented with Mini EDTA-free Protease Inhibitor (04693132001, Roche, Penzberg, Germany) and phosphatase inhibitors (04906837001, Carl Roth, Karlsruhe, Germany). After denaturation, proteins were fractioned on 10% SDS-polyacrylamide gels and transferred on nitrocellulose membranes. After blockade with 5% BSA (T844.3, Carl Roth, Karlsruhe, Germany), the membranes were incubated with primary antibody (Table S1) at 4°C overnight. After washing 3x with washing buffer, ECL IgG horse-reddish peroxidase coupled secondary antibody was applied for 1 hour at room temperature (Table S1). The detection was performed with ECL Plus Western Blotting substrate. After scanning of the films, the density of the target bands was measured with the ImageJ software (Version 1.44, Wayne Rasband, NIH, USA). Protein content in supernatants was measured with MCP-1/CCL2 ELISA kits (Sigma-Aldrich, Taufkirchen, Germany, RAB0055) according to the manufacturer's instructions.

Inhibitors and recombinant proteins

For the treatment of cancer cells and mice, we used human recombinant MCP-1/CCL2 (SRP3109, Sigma), the CCR4 antagonist C 021 dihydrochloride (3581, Tocris), CCR2 antagonist RS504393, (2517, Tocris), FAK inhibitor GSK2256098 (S8523, Selleckchem), Src inhibitor Bosutinib (S1014, Selleckchem), ERK inhibitor AZD8330 (S2134, Selleckchem), or Paxillin inhibitor 6B345TTQ (B7438, Sigma).

FACS sorting of pancreatic tumors

Pancreas with tumours were dissected and transferred to RPMI 1640 (Gibco) with cOmplete™, Mini Protease Inhibitor Cocktail (Roche) on ice. After washing 2-3 times with 1xPBS, the tissue was minced with scissors on ice, centrifuged at 1500 rpm for 2 min and treated with a dissociation enzyme cocktail (2 mg/ml Dispase II (Sigma-Aldrich), 1 mg/ml Trypsin Inhibitor (Sigma-Aldrich) and 1 unit/ml DNase I (NEB) dissolved in PBS containing 5% fetal bovine serum (FBS; Gibco), at 37 °C with a shaking speed of 50 r. p.m. for 30 minutes. Dissociated cells were collected every 10 min, filtered with a 40-µm nylon cell strainer (Falcon) and washed twice with PBS. After depletion of red blood cells with RBC lysis buffer (Invitrogen), cells were washed with PBS, counted and stained with an antibody cocktail in FACS buffer (PBS containing 0.5% FCS) for 60 minutes on ice. To isolate specific cell populations, cells were stained with Cd45-PECy5.5, Gr1-PB, Cd11b-APCCy7, Cd31-FITC, Ly6A/E-PECy7, Cd328-APC and Cd140b-PE antibodies (all from eBiosciences, ThermoFisher) and sorted using the MoFloAstrios EQ High Speed Sorter (Beckman Coulter).

Ras activation assay

The Ras activation assay was performed on the KPC, TPAC, and HPDE cell lines using the RAS activation kit (17-218, Millipore). The cells were cultured in T175 flasks until reaching 60%-70% confluency. Subsequently, the cells were harvested and lysed using the Mg2+ lysis/wash buffer. Total cellular protein was isolated and diluted to a concentration of 1 µg/µl. Next, 500 µg of protein from each cell line was incubated with GTPγS as the positive control and GDP as the negative control, following the instructions provided in the kit. Additionally, 500 µg of total cell protein from each cell line, including the positive and negative samples, was incubated with Raf-1 ras binding domain agarose beads for 45 minutes at 4 degrees Celsius. The GTP-Ras beads were then centrifuged three times and washed using the Mg2+ lysis/wash buffer. Afterwards, the agarose beads from each group, and 20 µg of total cell protein from each cell line, were boiled with Laemmli sample buffer and subjected to immunoblot analysis.

Differential expression analysis using the TCGA dataset

The Cancer Genome Atlas (https://www.cancer.gov/tcga) RNA-seq gene expression counts were downloaded from the Genomic Data Commons Data Portal (GDC; https://portal.gdc.cancer.gov/). Only primary tumor samples were included for differential expression analysis. PNI labels for 159 samples were obtained from digitized pathology reports from GDC. The metadata and data repository of the TCGA-PDAC database includes digitized pathology reports that included the information on the perineural invasion status for 74 samples, 66 of which has been reported to show PNI, and 8 were described as non-PNI. Differential expression analysis was performed using DESeq2. Briefly, median ratio method was used to calculate size factors for RNA-seq raw count data. Dispersion estimates were obtained for negative binomial distribution. After fitting a Negative Binomial Generalized Linear Model using the size factors and dispersion estimates, the significance of the coefficients were estimated. For visualization, size factor normalized count values were used.

Spatial transcriptomics / NanoString GeoMx® Digital Spatial Profiler

In the spatial transcriptome analyses with murine DRG, DRGs T8 to T12 were extracted bilaterally from TPAC (Ela-TGFα; Ptf1a-Cre; Trp53$^{fl/fl}$;RelA$^{fl/fl}$, n=6, 47-54 weeks old), KPC (Ptf1a-Cre; LSL-Kras+/^{G12D}; Trp53$^{+/fl}$, n=3, 20-21 weeks old) and age-similar tumor-free control mice (Ela-TGFα; Trp53$^{fl/fl}$;RelA$^{fl/fl}$, n=3, 48-50 weeks old for TPAC and LSL-Kras+/^{G12D}; Trp53$^{+/fl}$, n=1, 20 weeks old; Trp53$^{+/fl}$, n=1, 21 weeks old and LSL-Kras+/^{G12D}; n=1, 22 weeks old for KPC). Following the extraction, DRGs were briefly washed in ice-cold, fresh PBS (D5262, Sigma-Aldrich) and fixated in freshly prepared 4% PFA (15710, Electron Microscopy Sciences; diluted with PBS) for 24 hours at room temperature. The samples were consecutively dehydrated, paraffine embedded and sectioned. A whole transcriptome analysis was performed on NeuN rich regions within the DRG sections using the NanoString GeoMx® digital spatial profiler. 5µm thick unstained sections on slides were subjected to incubation for 35 minutes at 60°C. Afterwards, the slides were moved into Leica BOND Rx. Proteinase K (1ug/ml, for 5 minutes) and HIER2 (20 minutes) were used for RNA antigen retrieval. NanoString GeoMx Mouse Whole Transcriptome Atlas RNA probes were added for

hybridization for 16 hours at 37°C. Following the stringent washes per manufacturer's instructions, slides were blocked with W buffer for 1 hour. The slides were then stained with AF647 anti-NeuN (1:100, ab190565, Abcam), AF594 anti-Cd68 (1:200, sc-20060, Santa Cruz), AF532 Syto83 (1:10, ThermoFisher) and AF488 anti-GFAP (1:200, NBP2-3318AF488, Novus) antibodies. After washing the slides twice with SSC buffer, slides were loaded to the DSP and scanned for the regions of interest (ROI) selection. Two NeuN rich region per mouse were selected as ROIs.

For the human spatial transcriptome studies, two patients with pancreatic ductal adenocarcinoma were selected for transcriptome spatial analysis using the NanoString GeoMx Digital Spatial Profiler (DSP)/Human NGS Whole Transcriptome Atlas. Cases were selected based on the presence of perineural invasion in tissue sections. Each slide was stained with fluorescence-labeled antibodies to allow identification of tissue: pan-cytokeratin for epithelial cells and S100 for nerves. Tumor and Nerve areas of interest (AOI) were selected as follows: (i) NI Tumor: nerve-invading pancreatic (N=6); (ii) no NI Tumor: non-invading tumor cells (n=7); (iii) NI Nerve: tumor-invaded nerve (n=5); (iv) no NI Nerve: non-invaded nerve (n=4). AOIs were drawn manually around nerves and tumor cells. Following the ROI selection, UV-photocleaved oligonucleotide barcodes from each ROI were collected and prepared by the NanoString GeoMx protocol. Sequencing was performed on the NextSeq 2000. Raw basecell files were demultiplexed into fastsq files. NanoString's GeoMx NGS Pipeline v.2.0.0 was used to convert the fastsq files to Digital Count Conversion files. None of the 45 AOIs were below the warning sequencing saturation of 50%. Data was then normalized, and the downstream gene expression analyses were performed using the R package DESeq2.

Additional references pertinent to the methods

1. Ouyang H, Mou L, Luk C, Liu N, Karaskova J, Squire J, et al. Immortal human pancreatic duct epithelial cell lines with near normal genotype and phenotype. *Am J Pathol.* 2000;157(5):1623-31.
2. Furukawa T, Duguid WP, Rosenberg L, Viallet J, Galloway DA, and Tsao MS. Long-term culture and immortalization of epithelial cells from normal adult human pancreatic ducts transfected by the E6E7 gene of human papilloma virus 16. *Am J Pathol.* 1996;148(6):1763-70.

3. Liebl F, Demir IE, Rosenberg R, Boldis A, Yildiz E, Kujundzic K, et al. The severity of neural invasion is associated with shortened survival in colon cancer. *Clin Cancer Res.* 2013;19(1):50-61.
4. Demir IE, Boldis A, Pfitzinger PL, Teller S, Brunner E, Klose N, et al. Investigation of Schwann cells at neoplastic cell sites before the onset of cancer invasion. *J Natl Cancer Inst.* 2014;106(8).
5. Wang K, Demir IE, D'Haese JG, Tieftrunk E, Kujundzic K, Schorn S, et al. The neurotrophic factor neurturin contributes toward an aggressive cancer cell phenotype, neuropathic pain and neuronal plasticity in pancreatic cancer. *Carcinogenesis.* 2014;35(1):103-13.
6. Fangmann L, Teller S, Stupakov P, Friess H, Ceyhan GO, and Demir IE. 3D Cancer Migration Assay with Schwann Cells. *Methods Mol Biol.* 2018;1739:317-25.
7. Irizarry RA, Hobbs B, Collin F, Beazer-Barclay YD, Antonellis KJ, Scherf U, et al. Exploration, normalization, and summaries of high density oligonucleotide array probe level data. *Biostatistics.* 2003;4(2):249-64.
8. Wickham H. *ggplot2: Elegant Graphics for Data Analysis.*: Springer-Verlag New York; 2016.
9. Ceyhan GO, Liebl F, Maak M, Schuster T, Becker K, Langer R, et al. The severity of neural invasion is a crucial prognostic factor in rectal cancer independent of neoadjuvant radiochemotherapy. *Ann Surg.* 2010;252(5):797-804.
10. Liebl F, Demir IE, Mayer K, Schuster T, D'Haese JG, Becker K, et al. The impact of neural invasion severity in gastrointestinal malignancies: a clinicopathological study. *Ann Surg.* 2014;260(5):900-7; discussion 7-8.
11. Liebl F, Demir IE, Rosenberg R, Boldis A, Yildiz E, Kujundzic K, et al. The severity of neural invasion is associated with shortened survival in colon cancer. *Clin Cancer Res.* 2012:Nov 12. [Epub ahead of print].

P-paxillin

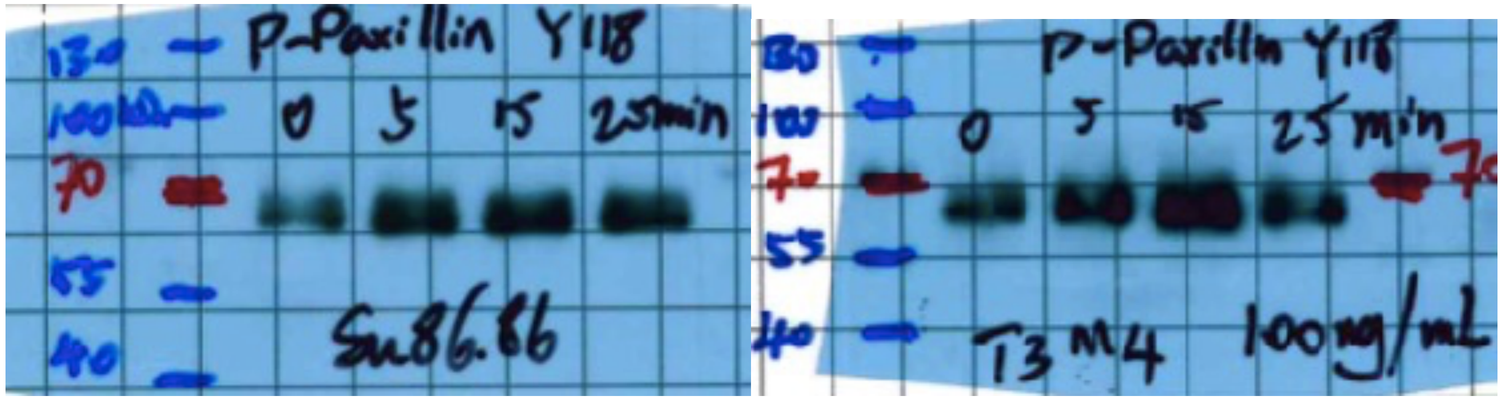

Paxillin

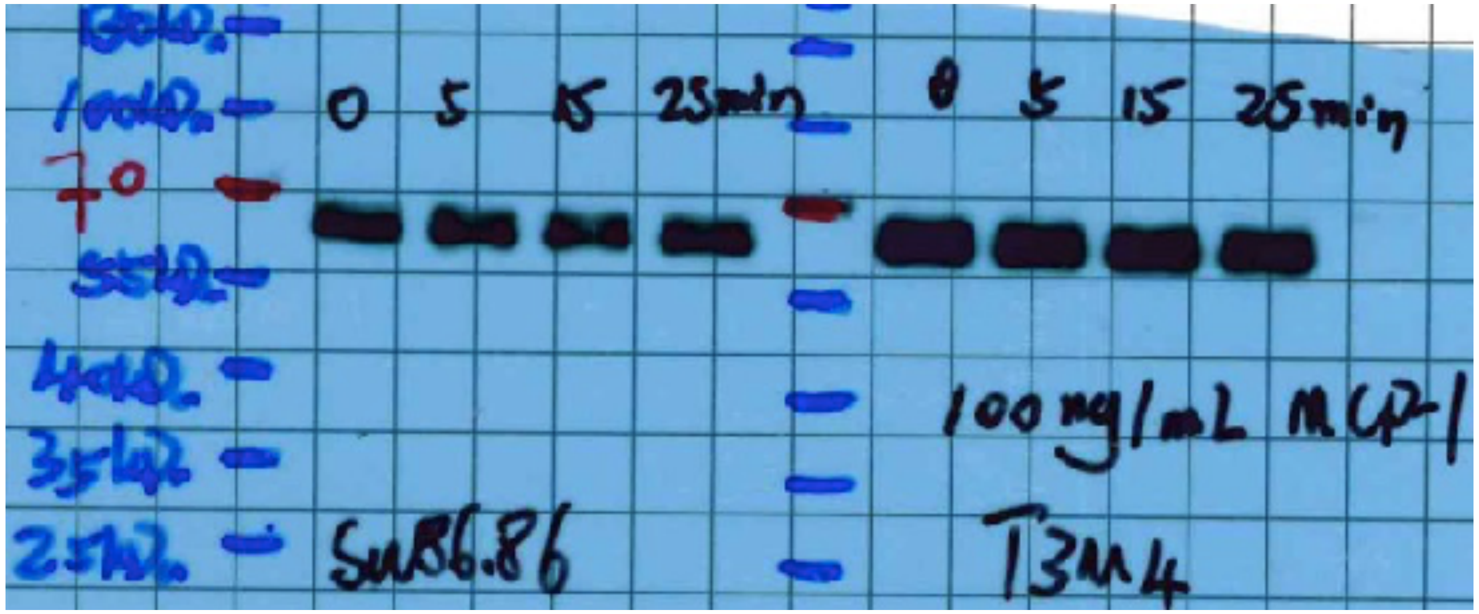

GAPDH

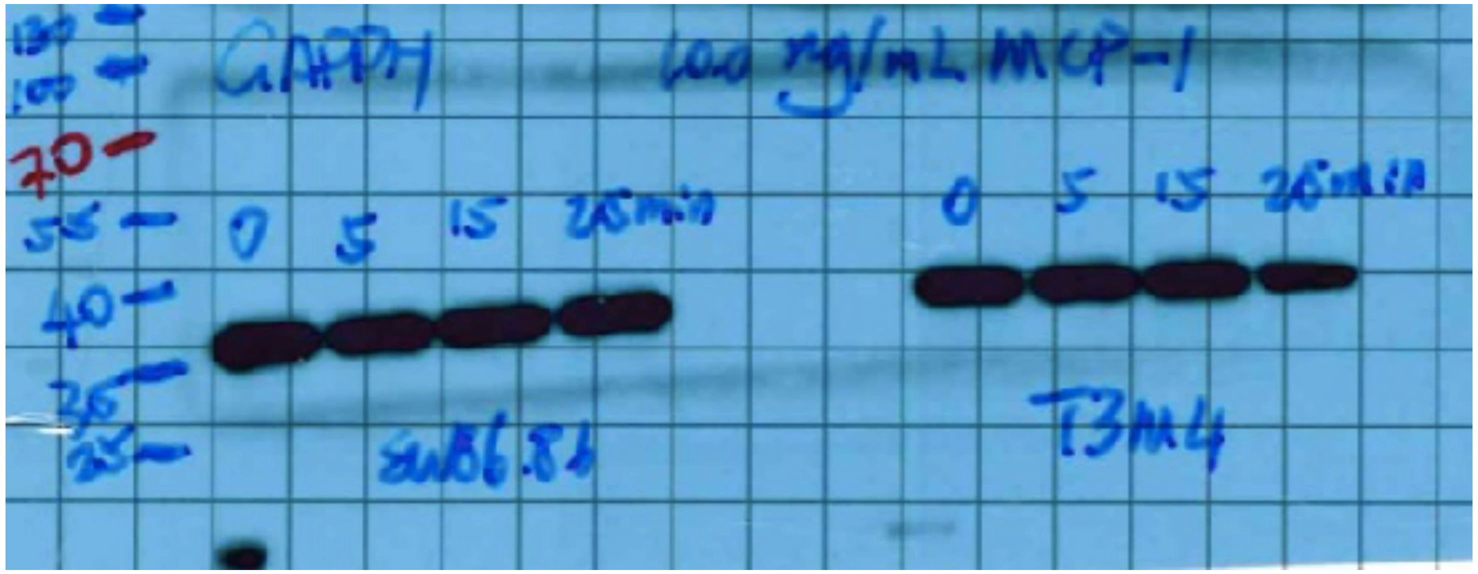

Unedited gel for Fig 3F

P-paxillin

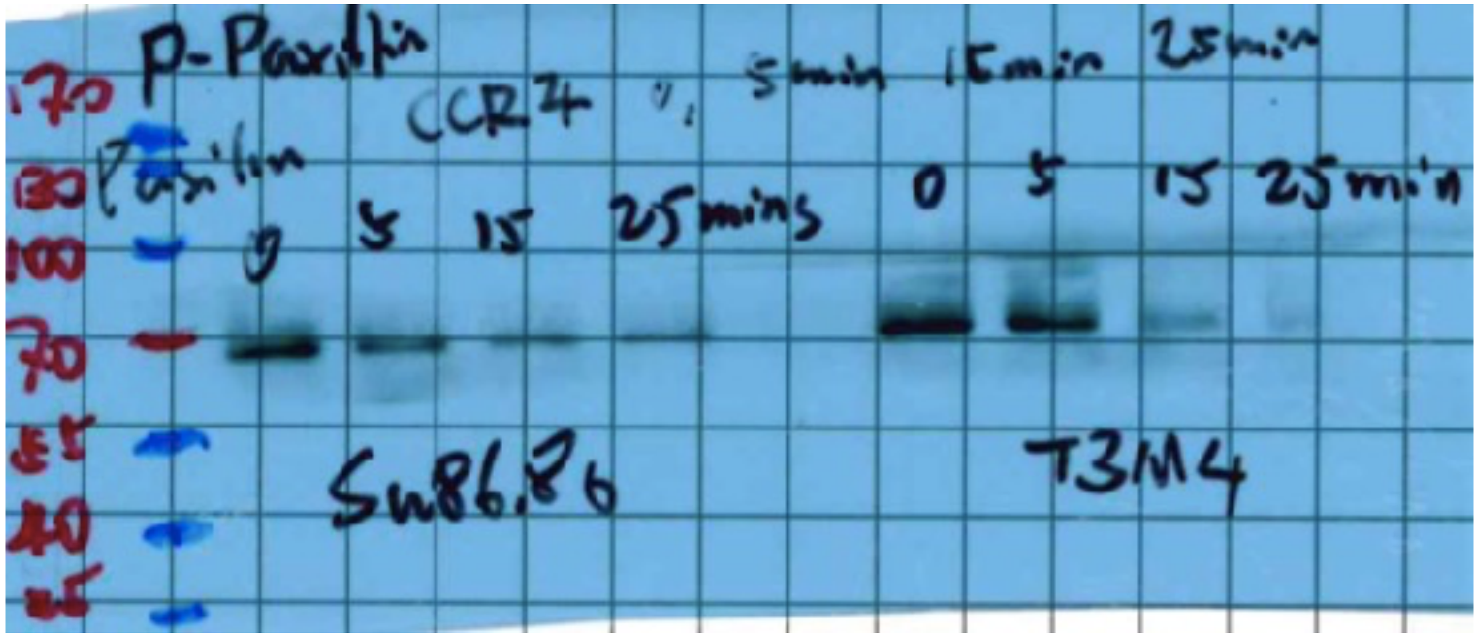

Paxillin

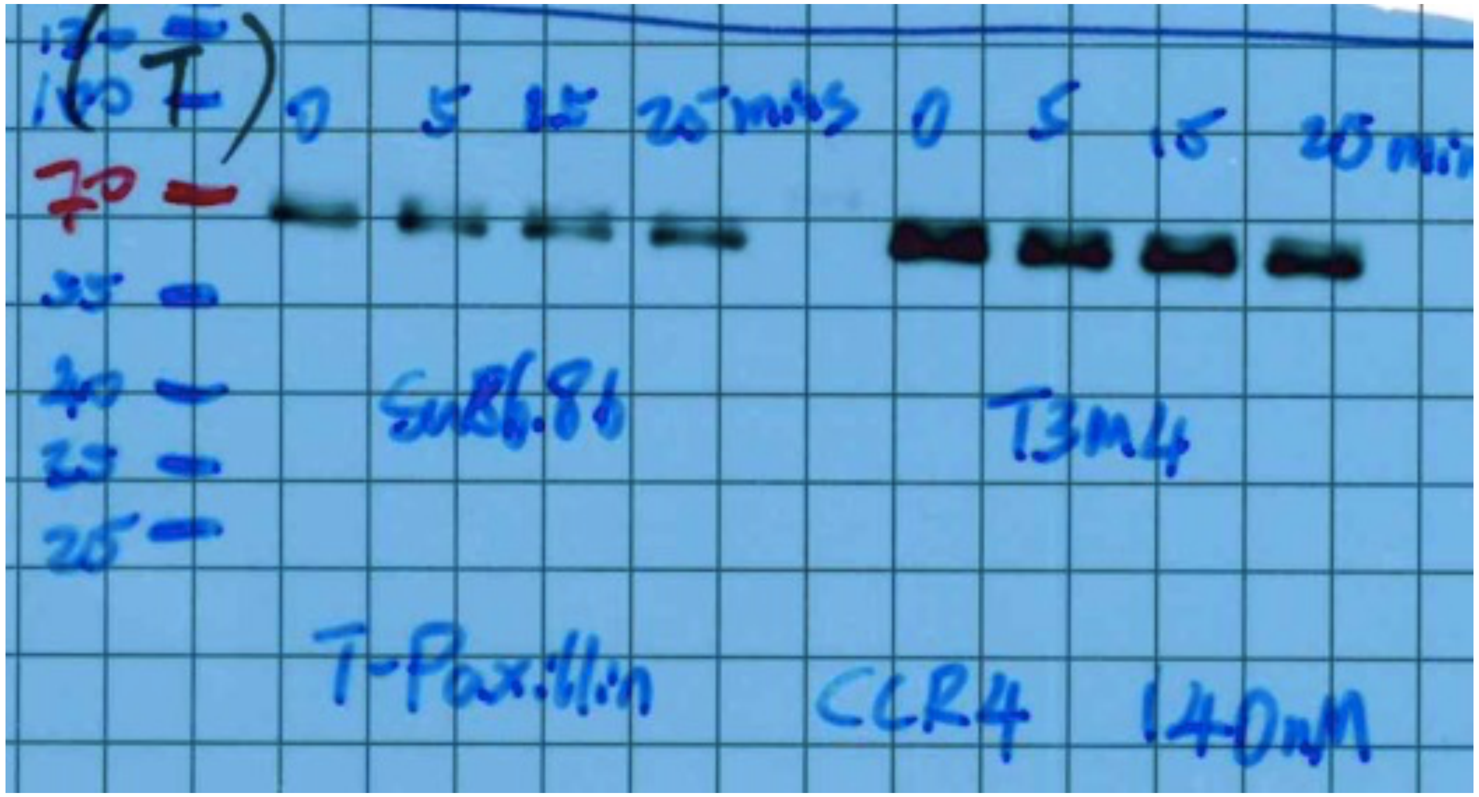

GAPDH

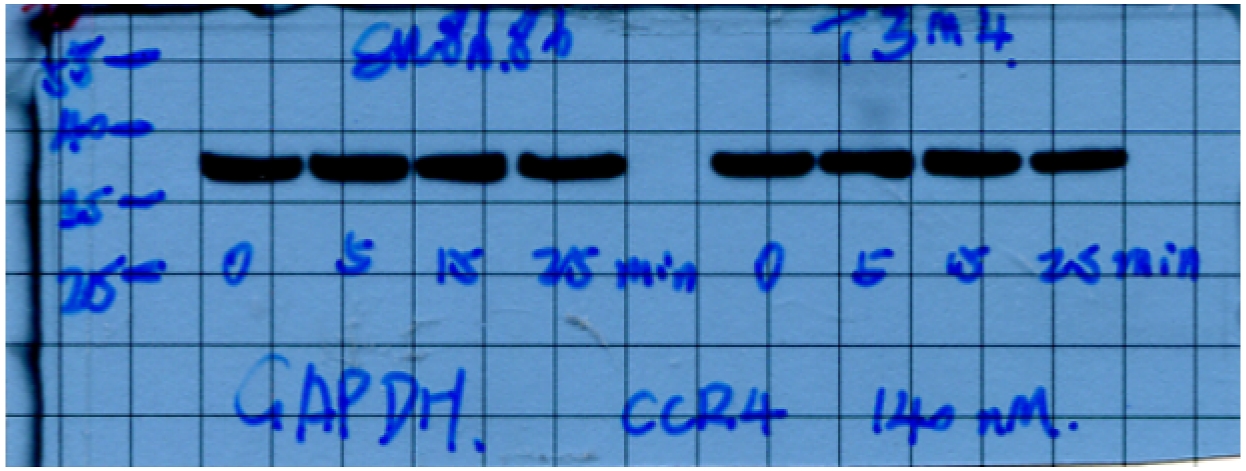

Unedited gel for Figure 3H

P-paxillin

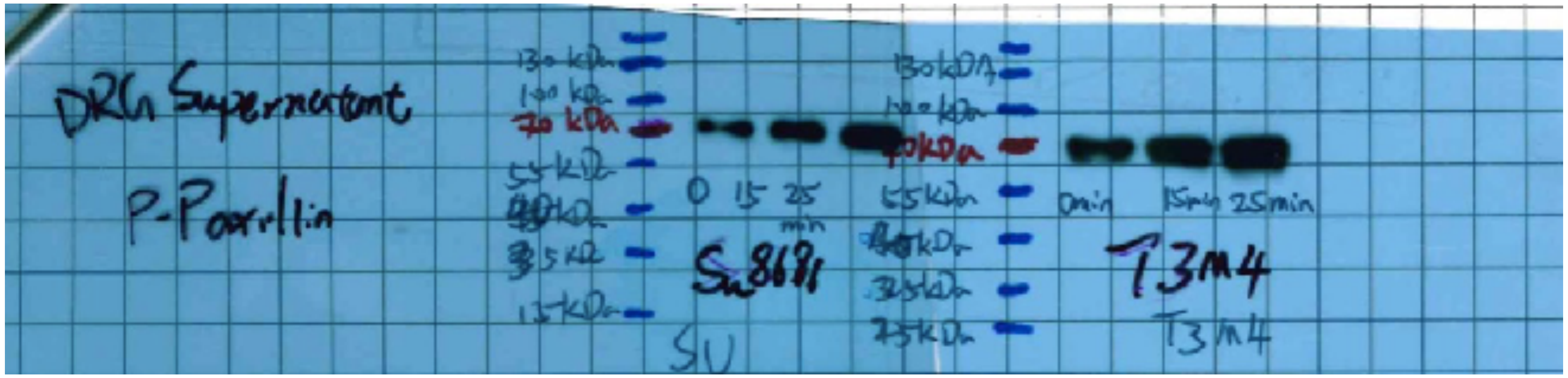

Paxillin

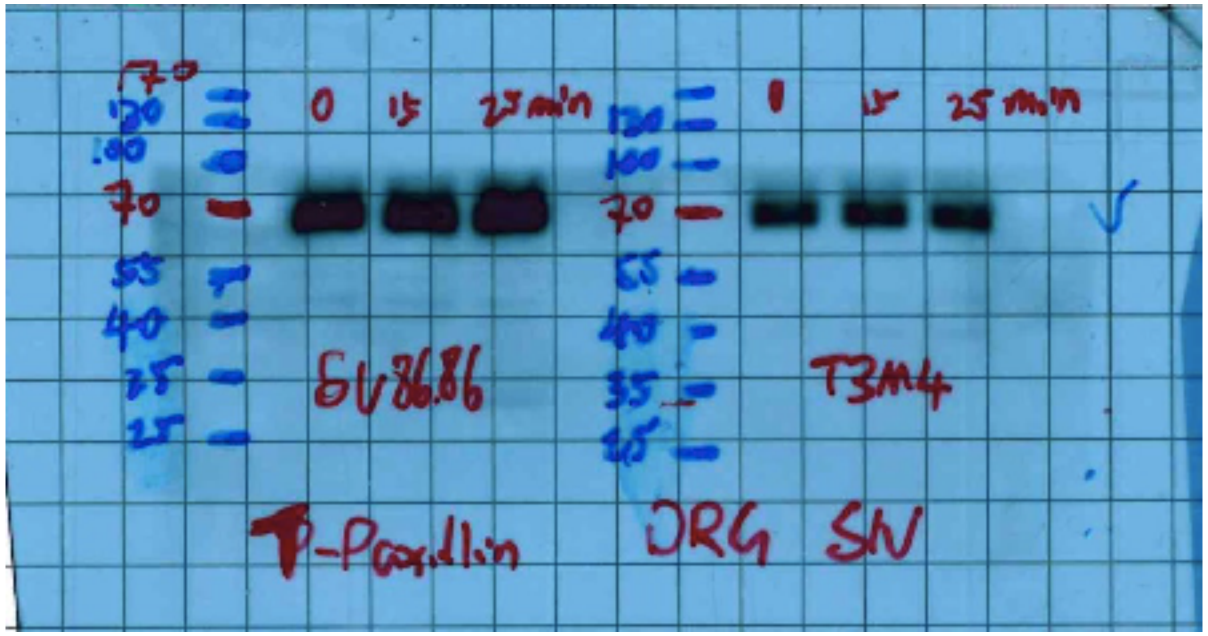

GAPDH

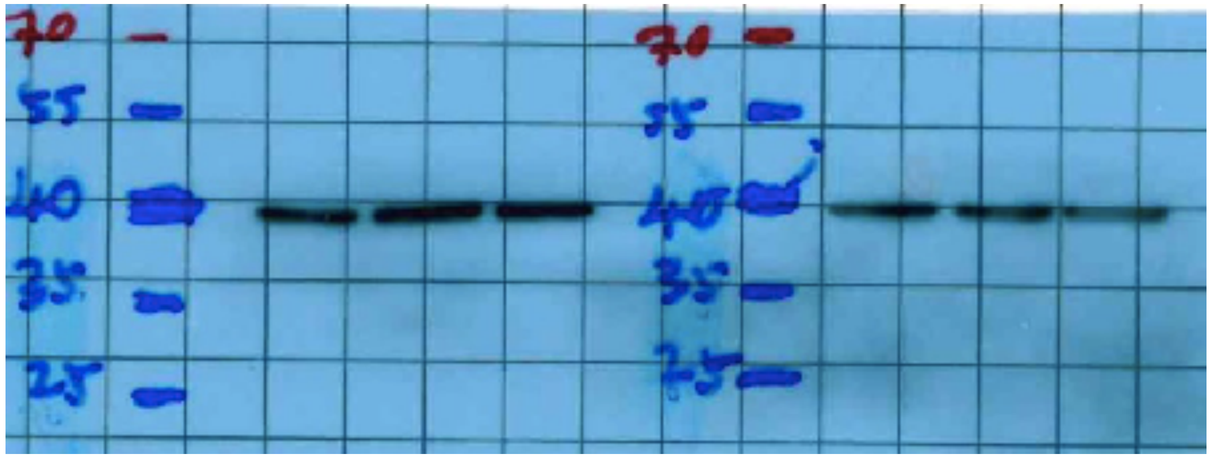

Unedited gel for Fig S3E

P-Src

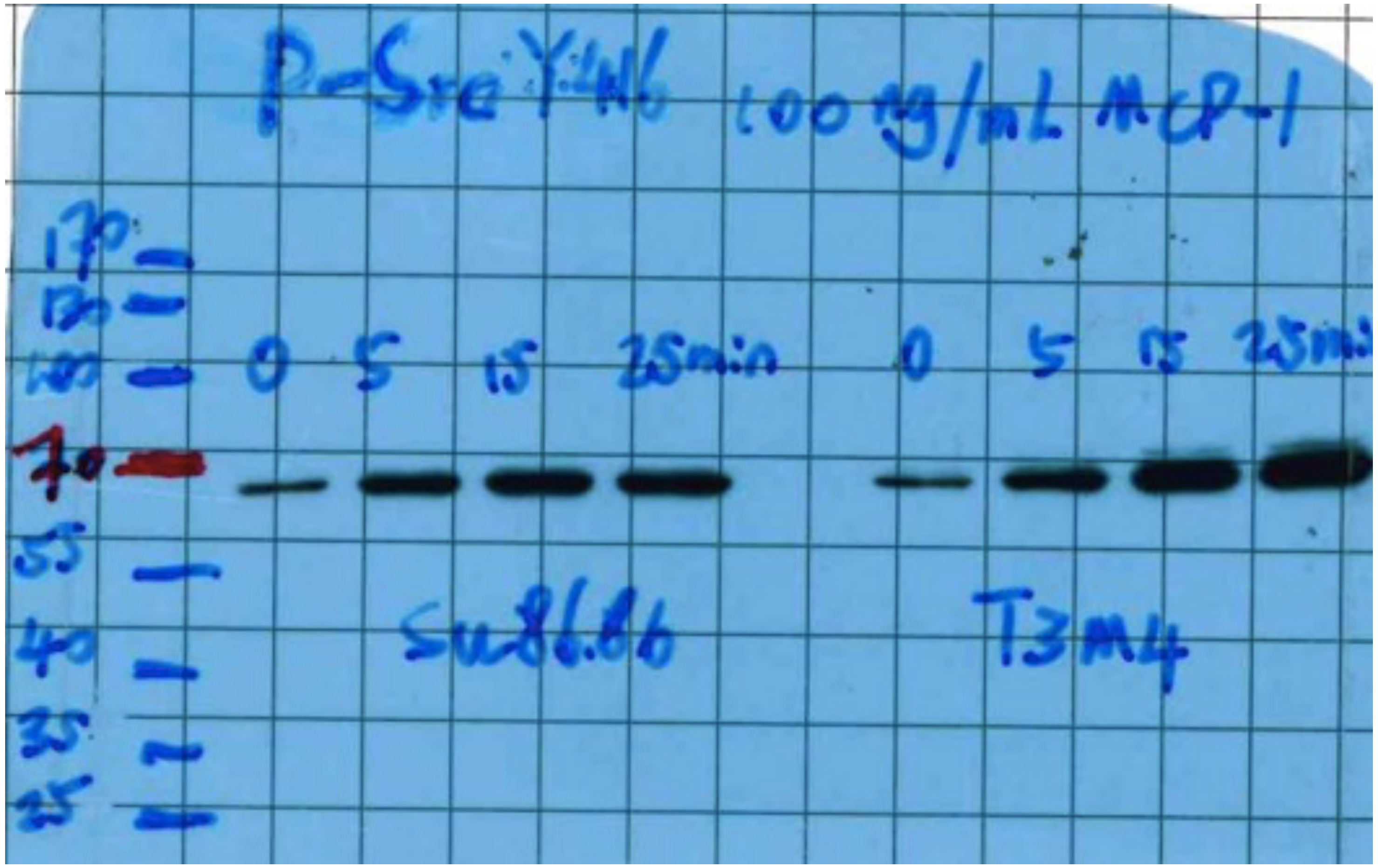

Src

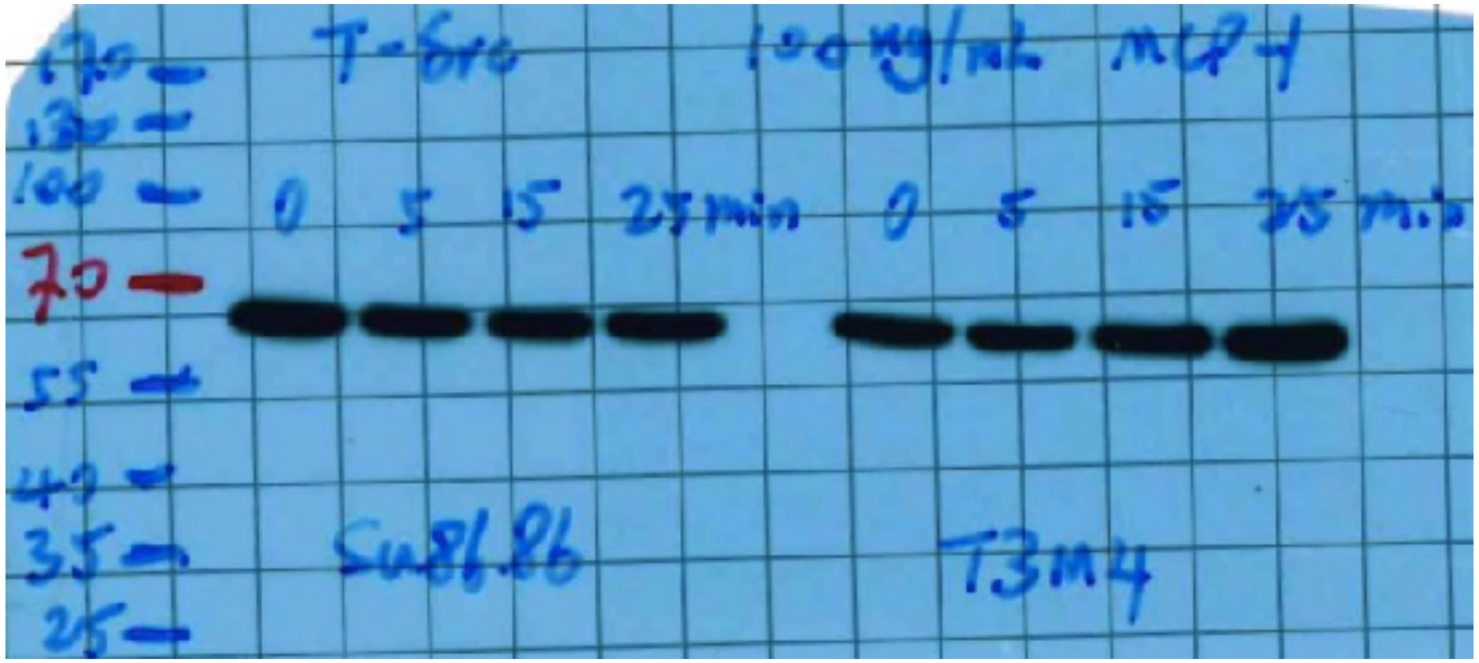

Unedited gel for Figure S3G

P-paxillin

Paxillin

GAPDH

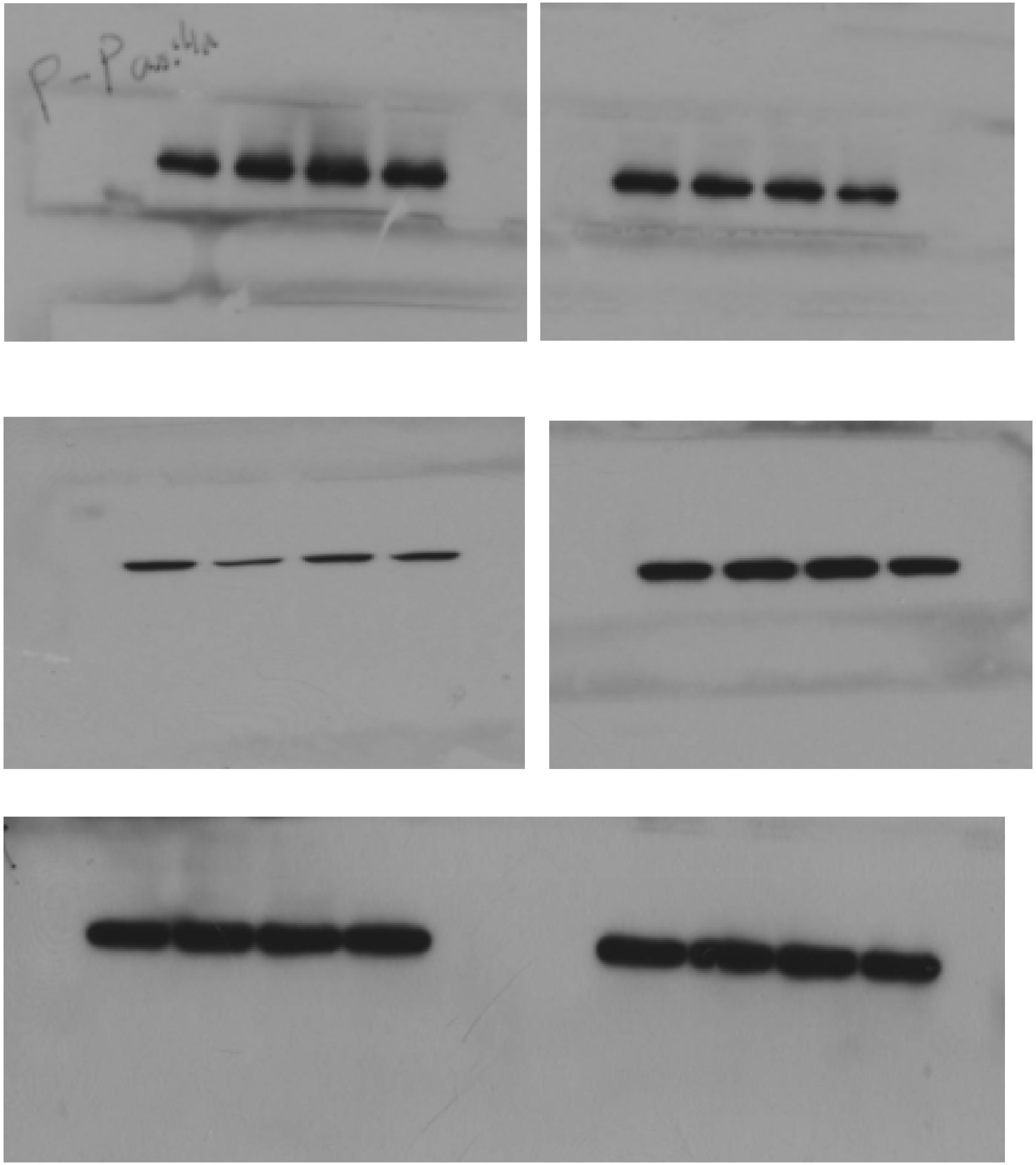

Unedited gel for Figure S3F

P-ERK1/2

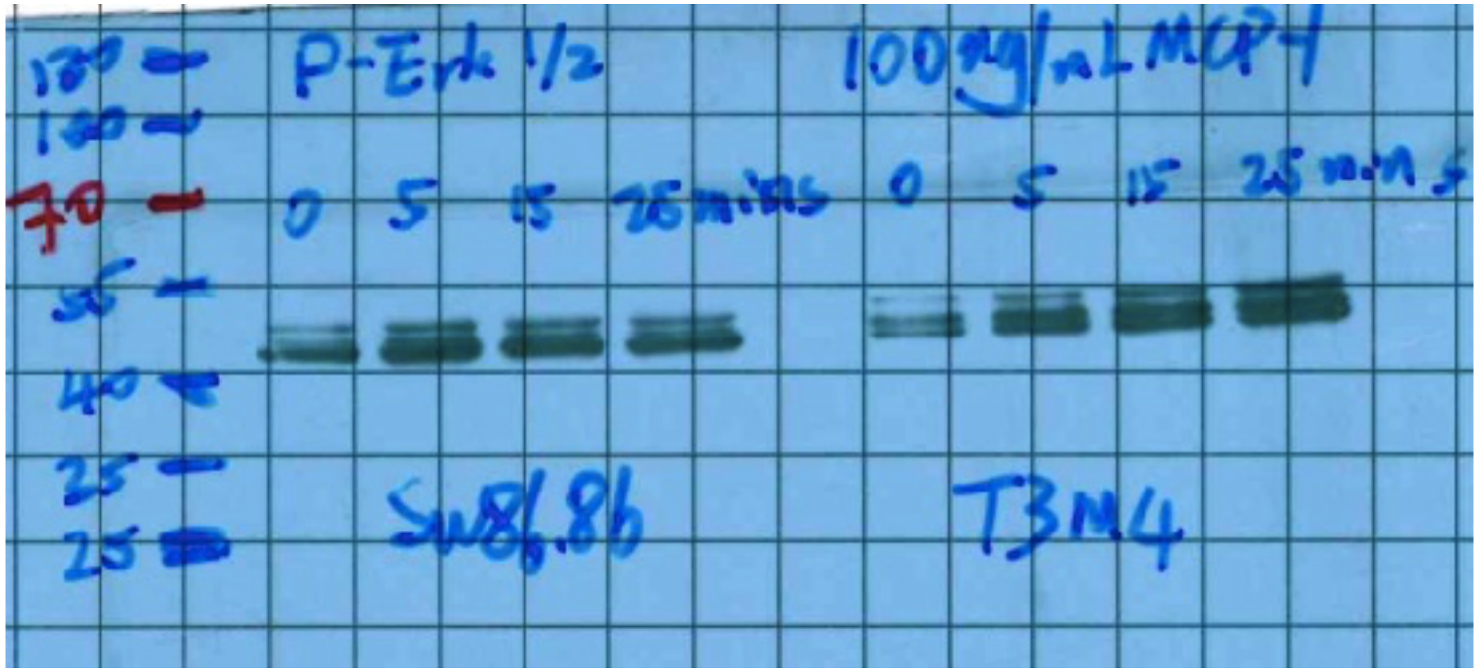

ERK1/2

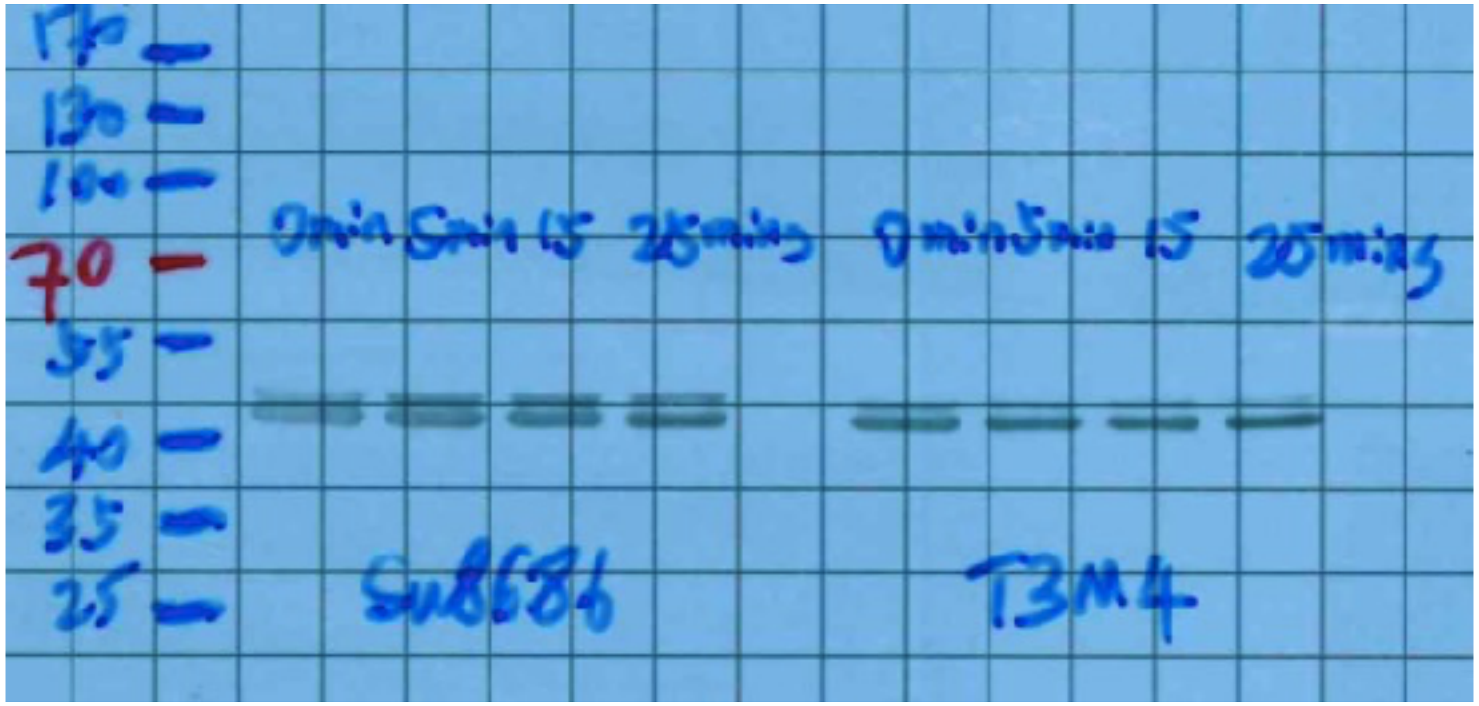

GAPDH

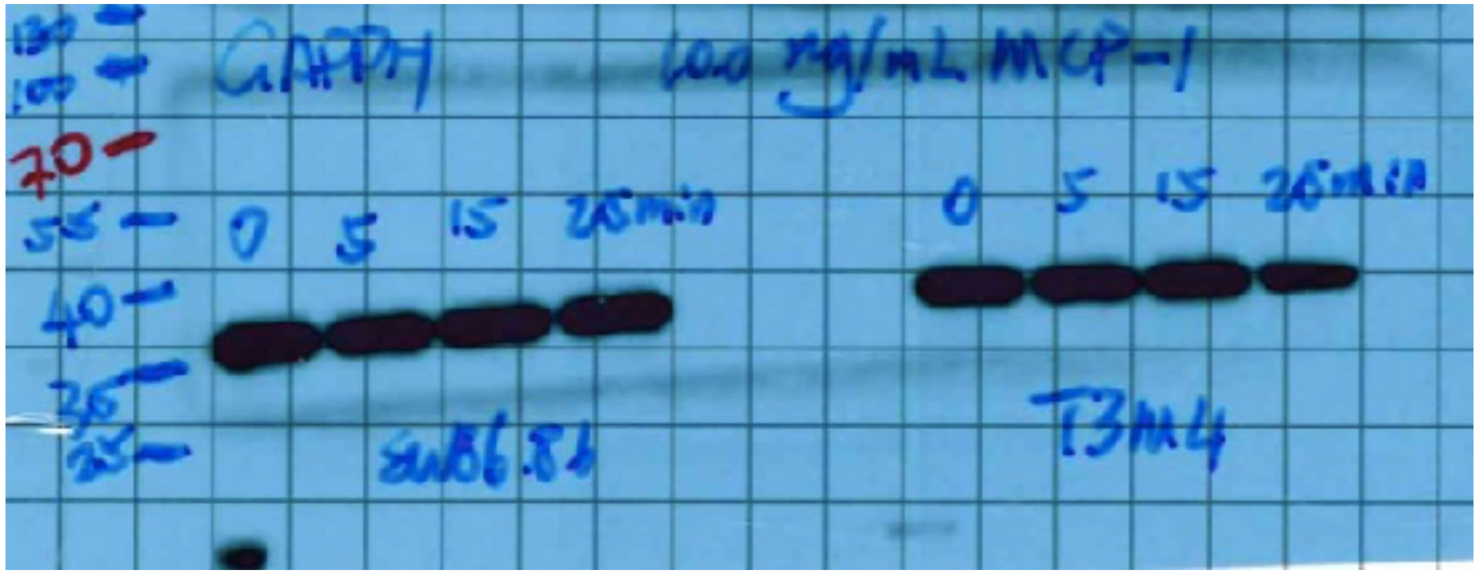

Unedited gel for Figure S3G

P-Src

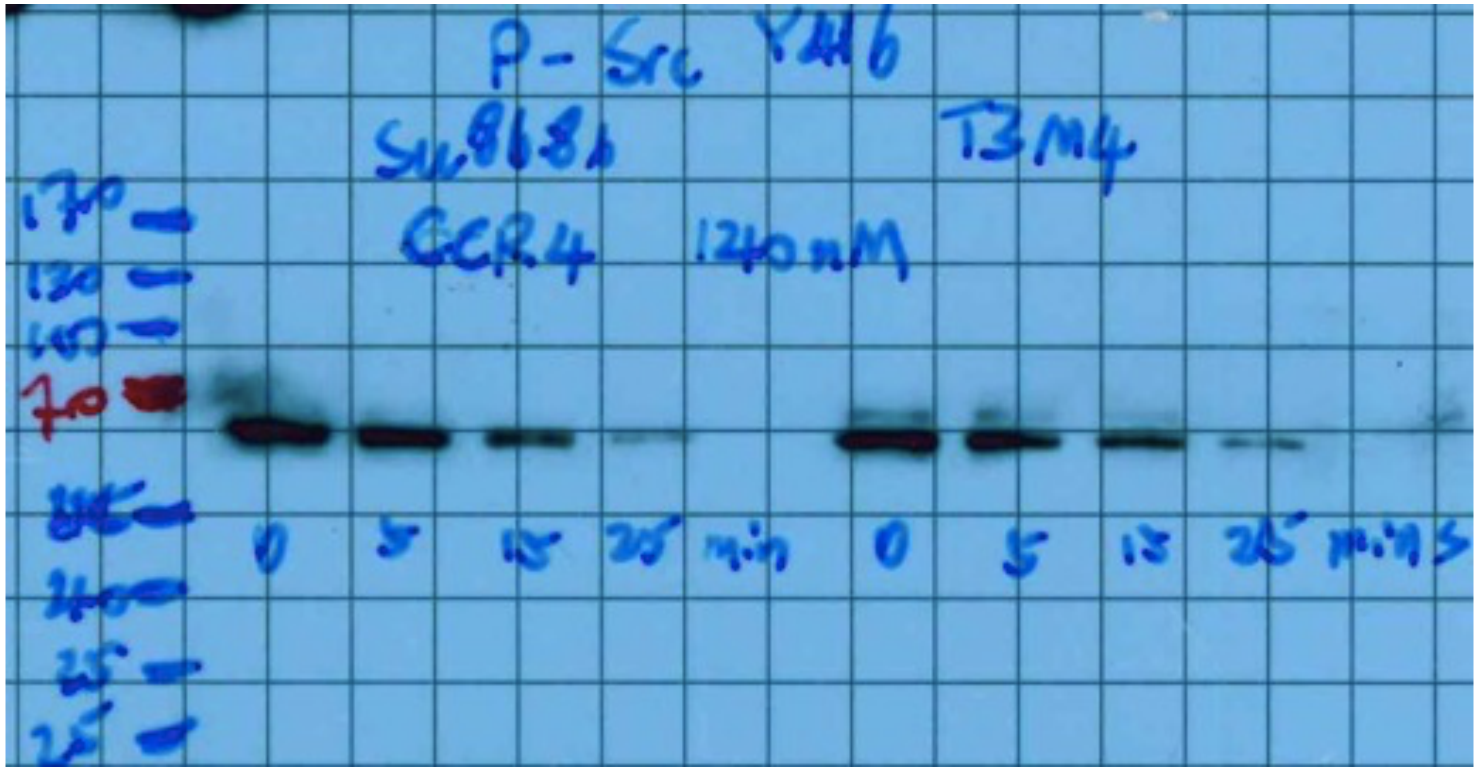

Src

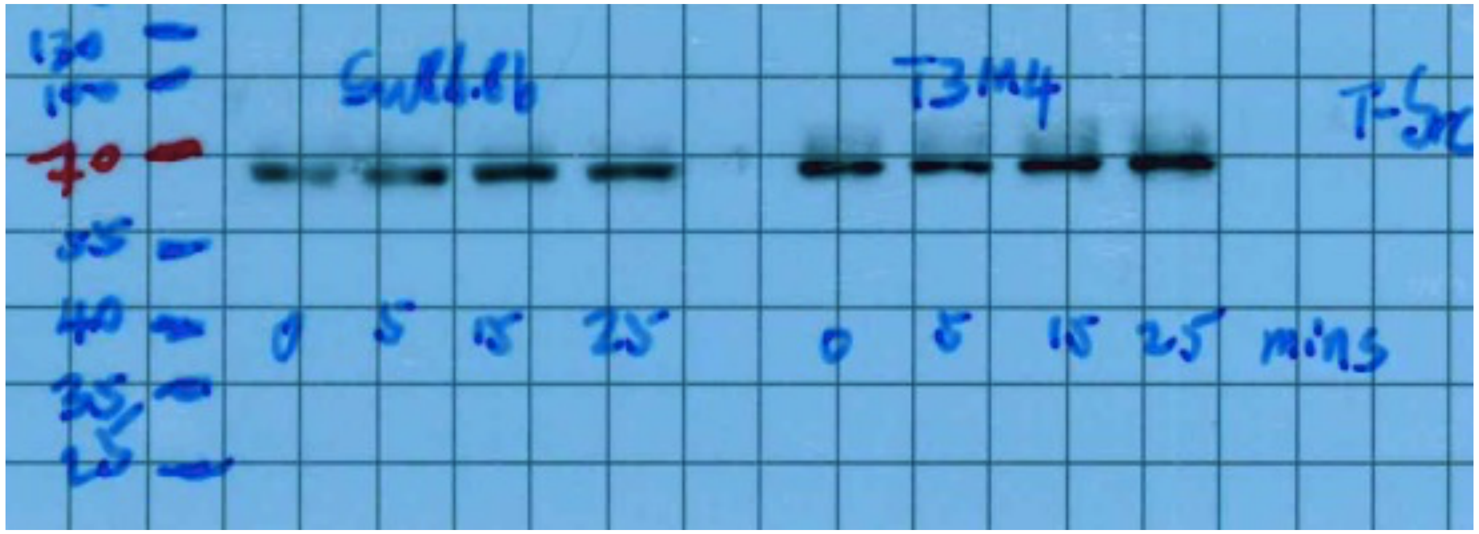

Unedited gel for Figure S3I

P-ERK1/2

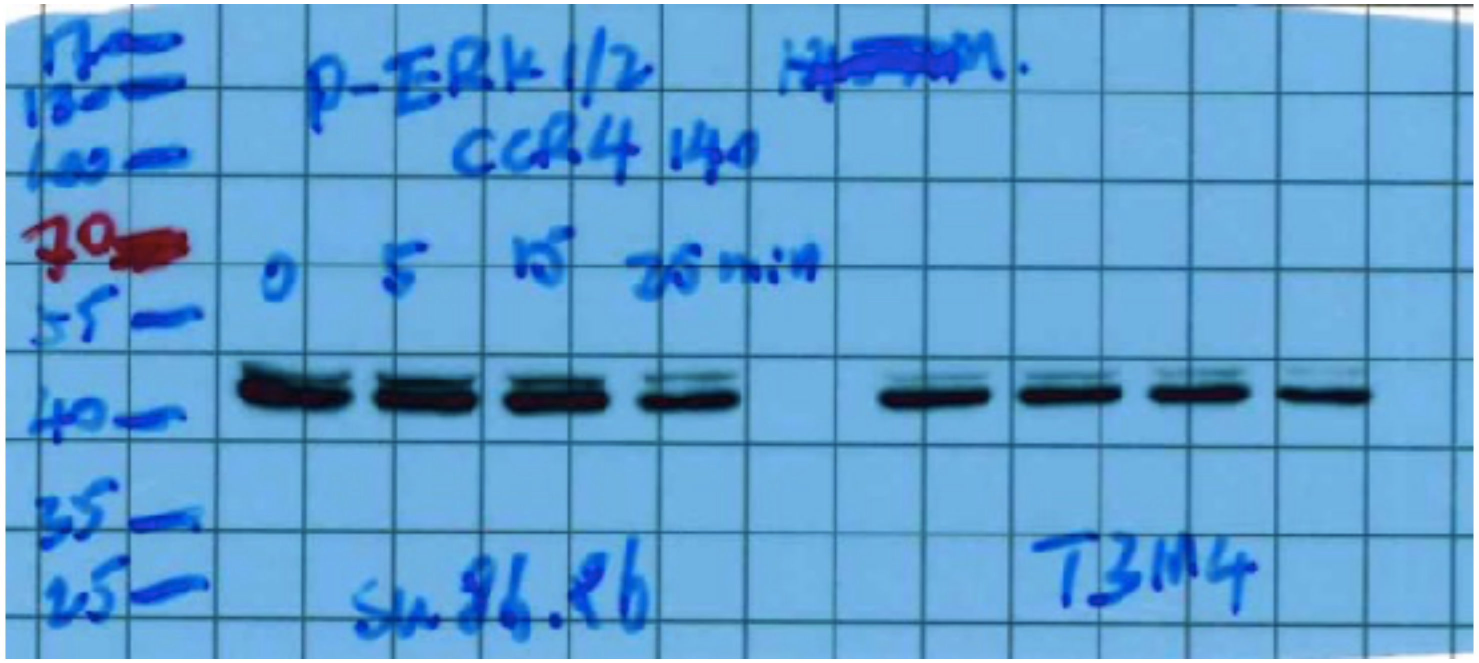

ERK1/2

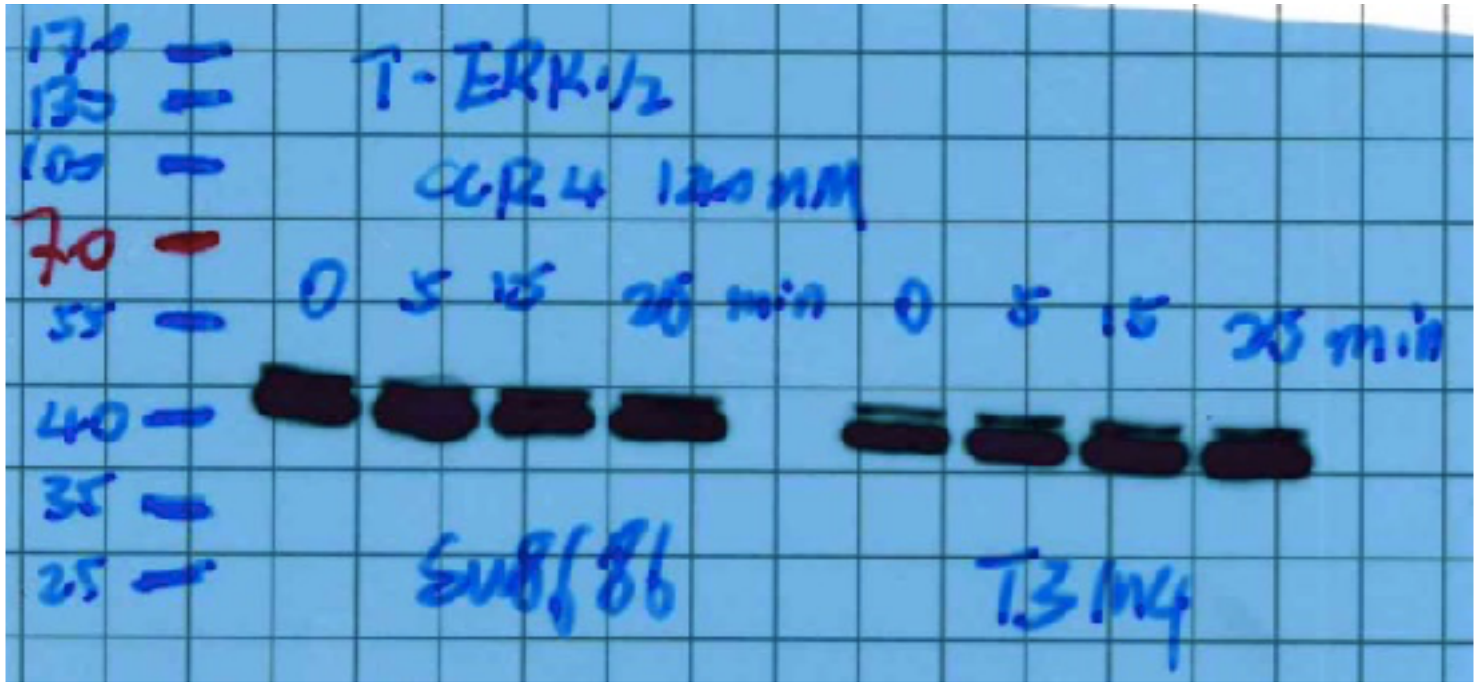

GAPDH

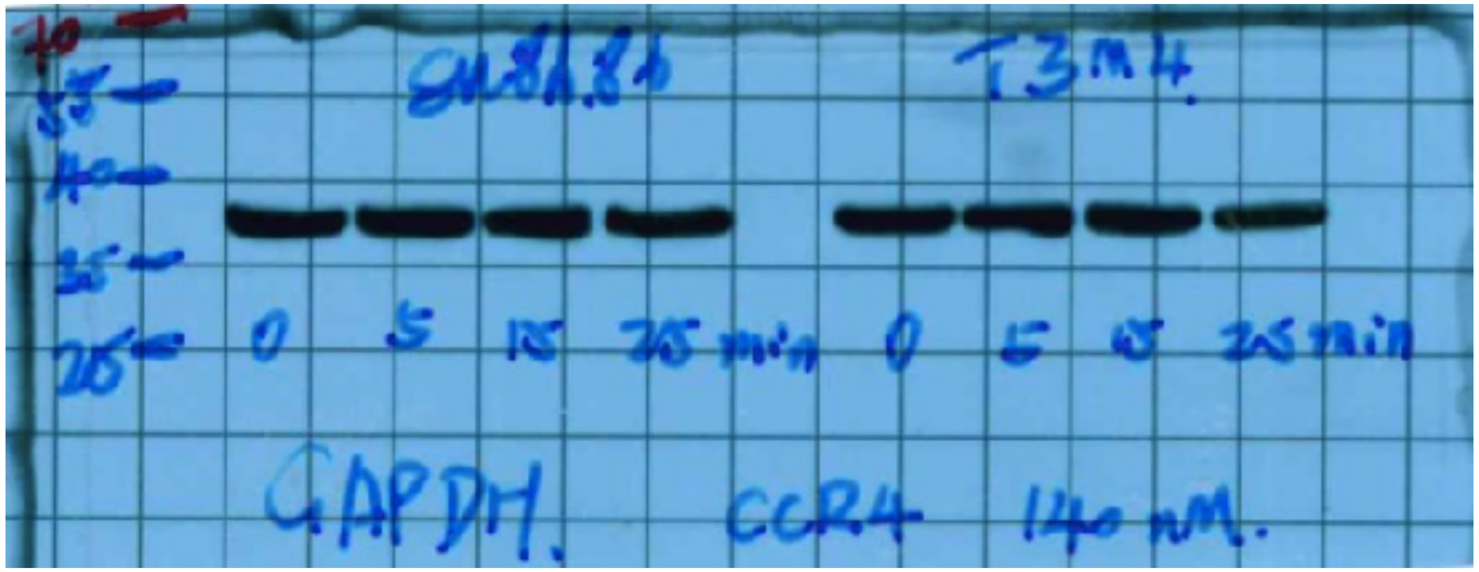

Unedited gel for Figure S3I

P-Paxillin

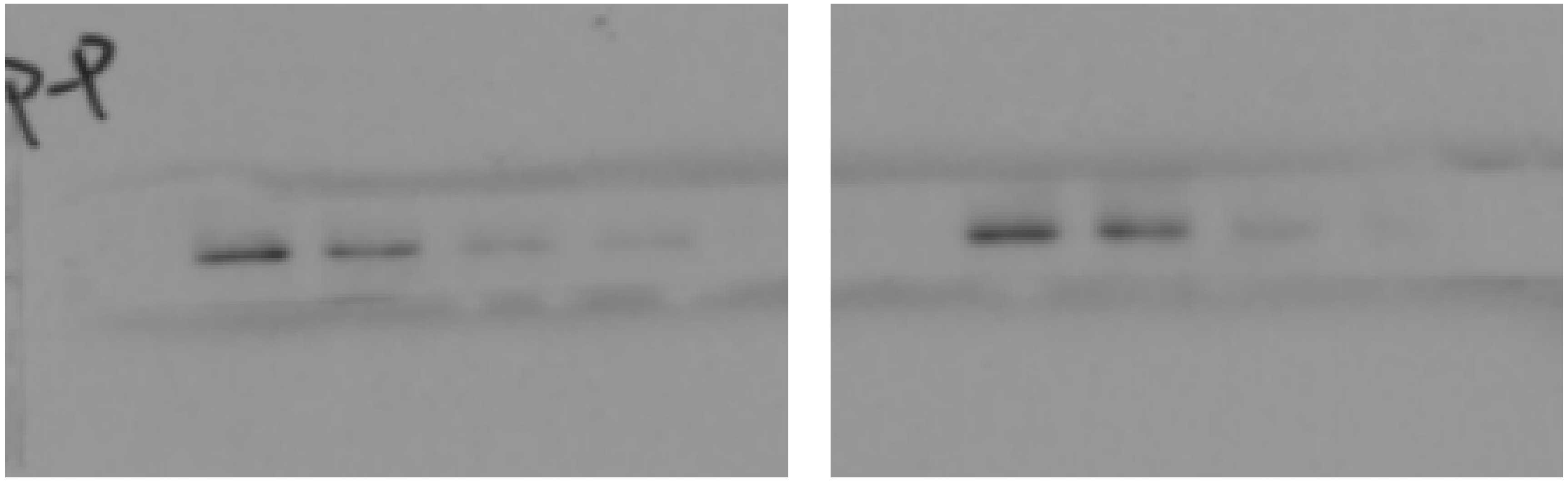

panxillin

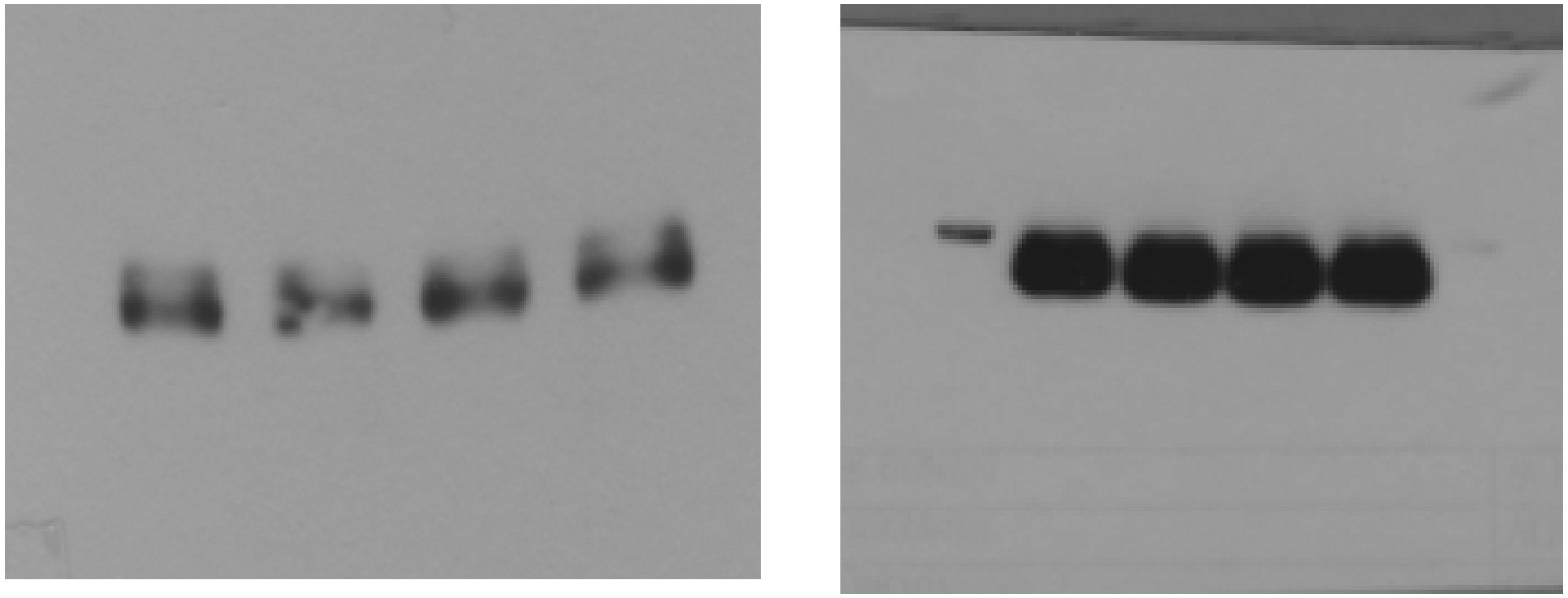

P-SRC

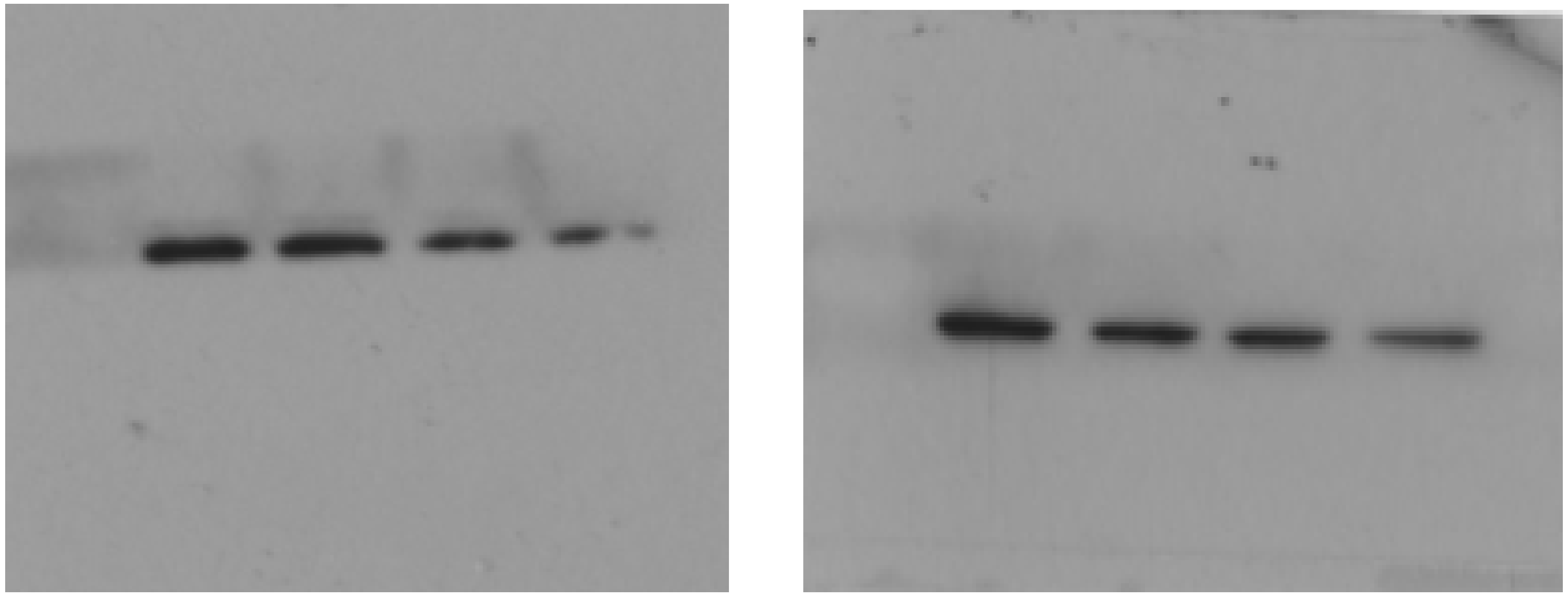

Unedited gel for Figure S6A

SRC

P-ERK

ERK

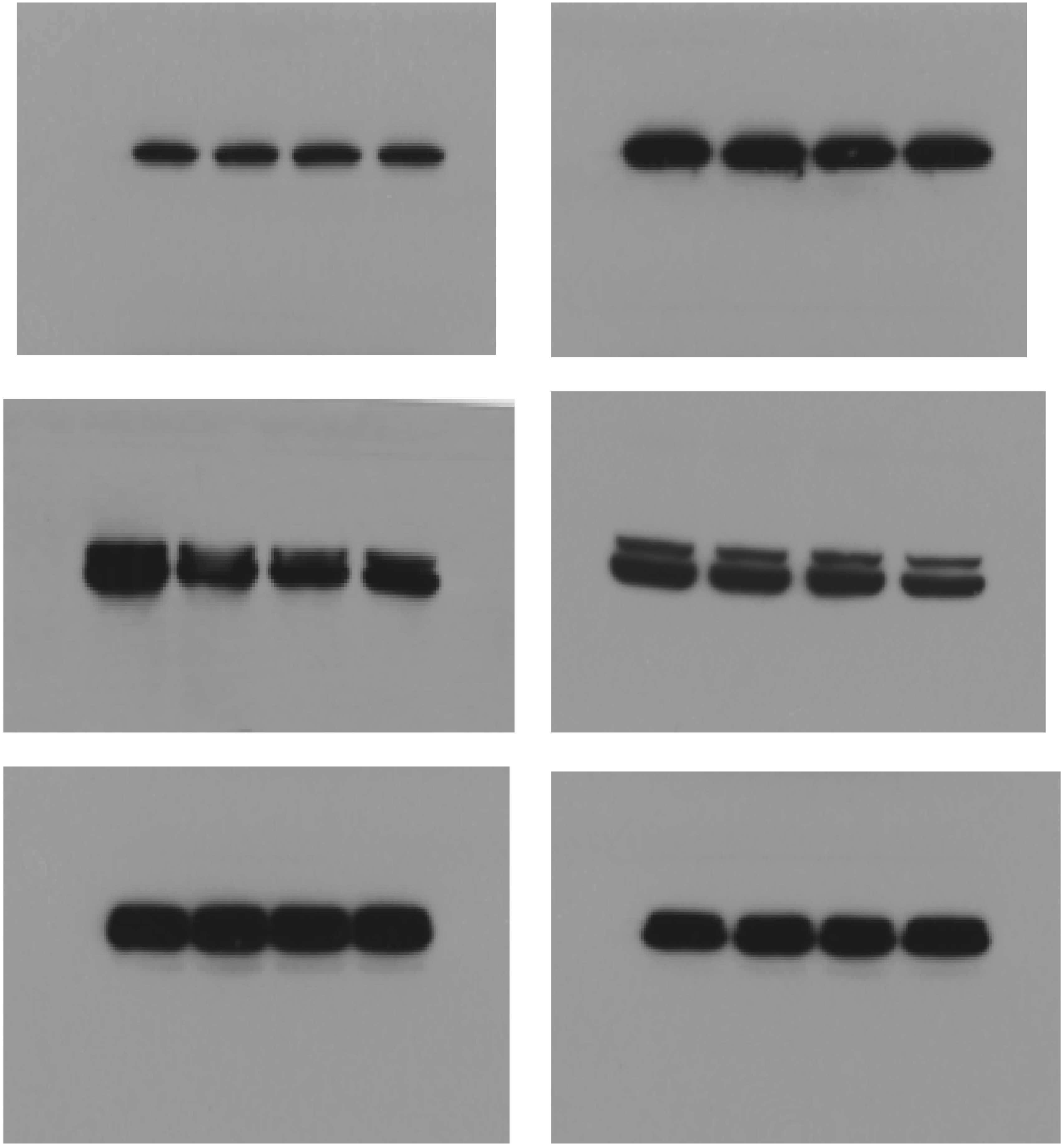

Unedited gel for Figure S6A

P-ERK1/2

ERK1/2

GAPDH

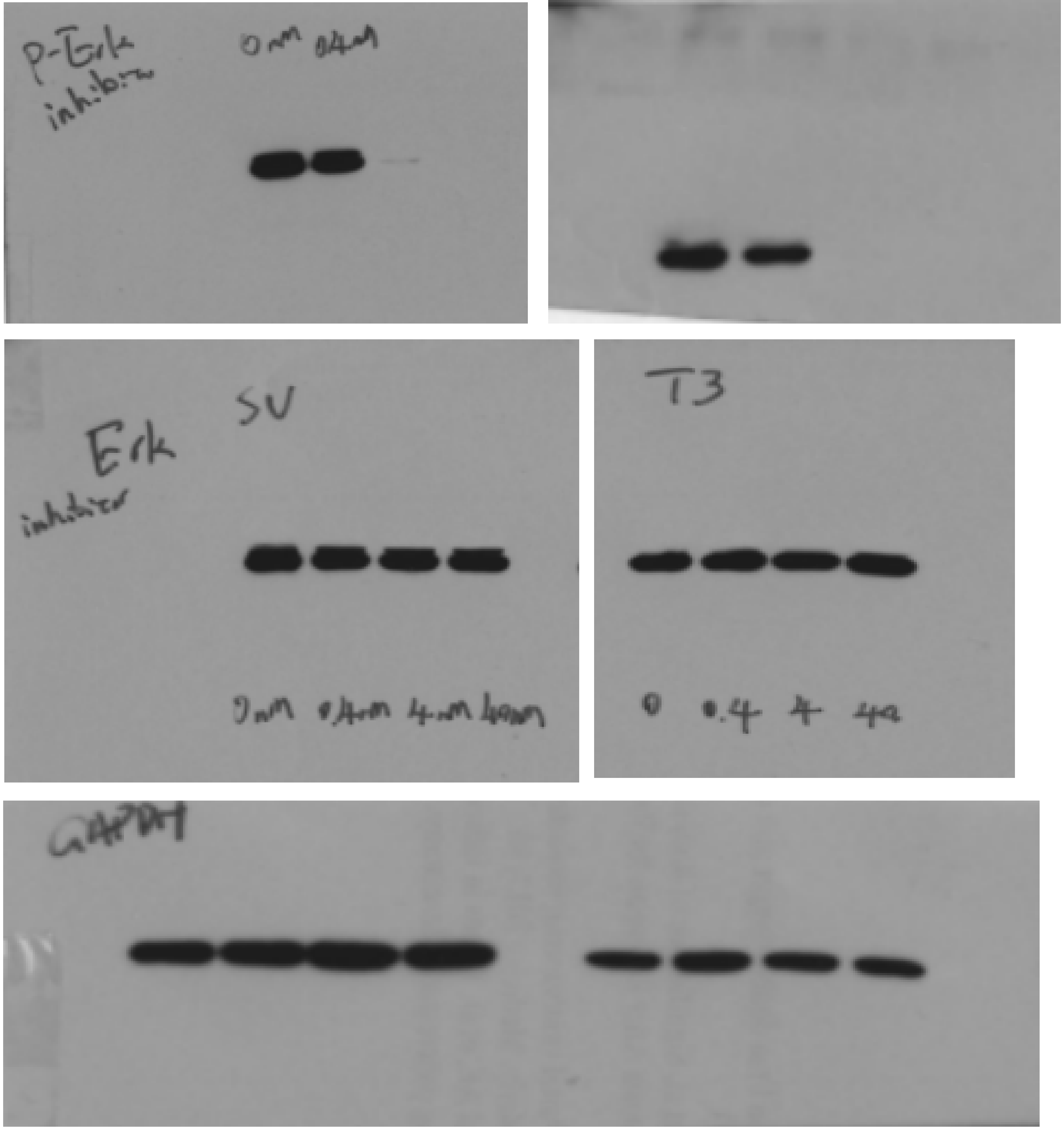

Unedited gel for Figure S6C
